# High density methylation QTL analysis in human blood via next-generation sequencing of the methylated genomic DNA fraction

**DOI:** 10.1186/s13059-015-0842-7

**Published:** 2015-12-23

**Authors:** Joseph L. McClay, Andrey A. Shabalin, Mikhail G. Dozmorov, Daniel E. Adkins, Gaurav Kumar, Srilaxmi Nerella, Shaunna L. Clark, Sarah E. Bergen, Swedish Schizophrenia Consortium, Christina M. Hultman, Patrik K. E. Magnusson, Patrick F. Sullivan, Karolina A. Aberg, Edwin J. C. G. van den Oord

**Affiliations:** Department of Pharmacotherapy and Outcomes Science, Virginia Commonwealth University, 410 North 12th Street, Richmond, VA 23298 USA; Center for Biomarker Research and Precision Medicine, Virginia Commonwealth University, 1112 East Clay Street, Richmond, VA 23298 USA; Department of Biostatistics, Virginia Commonwealth University, Richmond, Virginia USA; Department of Medical Epidemiology and Biostatistics, Karolinska Institute, Stockholm, Sweden; Psychiatric and Neurodevelopmental Genetics Unit, Massachusetts General Hospital, Boston, Massachusetts USA; Stanley Center for Psychiatric Research, Broad Institute of MIT and Harvard, Cambridge, Massachusetts USA; Department of Genetics, University of North Carolina School of Medicine, Chapel Hill, North Carolina USA

**Keywords:** DNA methylation, next-generation sequencing, GWAS, single nucleotide polymorphism, quantitative trait loci, chromatin states

## Abstract

**Background:**

Genetic influence on DNA methylation is potentially an important mechanism affecting individual differences in humans. We use next-generation sequencing to assay blood DNA methylation at approximately 4.5 million loci, each comprising 2.9 CpGs on average, in 697 normal subjects. Methylation measures at each locus are tested for association with approximately 4.5 million single nucleotide polymorphisms (SNPs) to exhaustively screen for methylation quantitative trait loci (meQTLs).

**Results:**

Using stringent false discovery rate control, 15 % of methylation sites show genetic influence. Most meQTLs are local, where the associated SNP and methylation site are in close genomic proximity. Distant meQTLs and those spanning different chromosomes are less common. Most local meQTLs encompass common SNPs that alter CpG sites (CpG-SNPs). Local meQTLs encompassing CpG-SNPs are enriched in regions of inactive chromatin in blood cells. In contrast, local meQTLs lacking CpG-SNPs are enriched in regions of active chromatin and transcription factor binding sites. Of 393 local meQTLs that overlap disease-associated regions from genome-wide studies, a high percentage encompass common CpG-SNPs. These meQTLs overlap active enhancers, differentiating them from CpG-SNP meQTLs in inactive chromatin.

**Conclusions:**

Genetic influence on the human blood methylome is common, involves several heterogeneous processes and is predominantly dependent on local sequence context at the meQTL site. Most meQTLs involve CpG-SNPs, while sequence-dependent effects on chromatin binding are also important in regions of active chromatin. An abundance of local meQTLs resulting from methylation of CpG-SNPs in inactive chromatin suggests that many meQTLs lack functional consequence. Integrating meQTL and Roadmap Epigenomics data could assist fine-mapping efforts.

**Electronic supplementary material:**

The online version of this article (doi:10.1186/s13059-015-0842-7) contains supplementary material, which is available to authorized users.

## Background

Methylation of DNA cytosine residues is an important mechanism in the control of gene expression and the determination of cell fate in development [1–3]. DNA methylation is known to vary with sex, age and exposure to environmental factors [4] and changes to methylation patterns have been associated with many common diseases [5]. Methylation is also under genetic influence and locus-specific methylation levels are often correlated in related individuals [6, 7]. This observation has motivated the mapping of loci where DNA methylation is under genetic control, also known as methylation quantitative trait loci (meQTLs).

Several early reports focusing on candidate loci found instances of DNA methylation levels correlated with sequence variants [8]. However, the advent of genome-wide association studies (GWAS) and methods for interrogating methylation at multiple loci has enabled the mapping of meQTLs on a larger scale [7, 9, 10]. Genotype array marker densities have increased dramatically over time and, when coupled with imputation, now enable comprehensive surveys of most common SNPs in the human genome [11]. Our ability to interrogate genome-wide DNA methylation (the “methylome”) has developed in parallel with these technologies [12, 13]. Most published genome-wide meQTL studies [14–16] measured methylation via the Illumina Infinium 27 K array, capable of interrogating ~27,000 methylated sites, while the most comprehensive study to date used the latest Infinium chip to analyze ~450,000 sites in lung tissue [17]. However, there are approximately 27 million autosomal CpGs in the human genome, of which a substantial portion is methylated in most tissues surveyed [18]. It is therefore apparent that only a small fraction of possible meQTLs has been surveyed to date. In addition, DNA methylation outside CpG islands, traditionally the focus of methylation research, plays a role in the regulation of transcription [19]. This suggests that more comprehensive meQTL surveys could be invaluable in understanding genetic regulatory processes.

In this context, next-generation sequencing (NGS) methods offer a significant advance over array-based methylation detection [12]. Whole genome shotgun bisulfite sequencing (WGBS) yields single base resolution methylation data for every cytosine in the genome, but it is not yet economically feasible in the large sample numbers required for genetic epidemiology [13]. This factor is particularly relevant for meQTL studies, where our ability to detect effects depends not only on the proportion of the genome covered by methylation and single nucleotide polymorphism (SNP) data, but also adequate statistical power derived from large sample numbers. As an alternative to WGBS, enrichment for the methylated genomic fraction followed by NGS can yield information on many millions of methylation sites [20]. In this study, therefore, we use methyl-CpG binding domain (MBD) protein-based enrichment coupled to NGS (MBD-seq) to assay DNA methylation in human blood [21]. MBD-seq has been demonstrated to be sensitive and capable of identifying differentially methylated regions [20–25], to detect previously reported robust associations [26], and to produce findings that replicate using more sensitive, targeted technologies [27]. Although MBD-seq cannot pinpoint the specific CpG that caused an association in regions with multiple CpGs, its resolution is approximately the size of the sequenced fragment (150–250 bp). All these properties make MBD-seq a very efficient tool for high-density methylome-wide studies.

We used MBD-seq to measure methylation at over 4.5 million unique loci in DNA from peripheral blood from normal subjects. We then tested each locus-specific DNA methylation measure for association with a high density SNP genotype panel, augmented with imputed genotypes from 1000 Genomes [28], to exhaustively identify common variant meQTLs. To control for unmeasured confounders in the methylation data, such as could be caused by heterogeneous cell types in the source tissue (i.e., blood), we included the top principal components from the methylation data as covariates in the association testing. Our study provides a comprehensive up-to-date overview of genetic influence on the methylome in human blood DNA, outside of a specific disease context, and provides insights into the processes that generate meQTLs.

## Results

### Data summary

Our study population comprised 697 subjects from Sweden (see sample description in Table S1 in Additional file 1) who were controls from a larger genetic study on the etiology of schizophrenia [27]. Due to the specific nature of sex chromosome methylation patterns, we focus on the autosomes. SNPs were genotyped as described previously [29, 30]. Imputation was carried out with Minimac [28] using 1000 Genomes reference panels v3 using minor allele frequency (MAF) > 0.05 and *r*^*2*^ > 0.5 as thresholds. Our MBD-seq data consisted of 31.6 million methylation-enriched reads per subject (standard deviation (SD) = 13.4 million) after alignment and quality control (QC). We used these reads to estimate fragment coverage at each of the ~27 million autosomal CpG sites in the human reference genome (hg19), where higher coverage indicates higher levels of methylation [31]. Our methylation data preprocessing involved exclusion of sites showing poor mappability, data reduction by combining highly correlated coverage estimates at neighboring CpGs [31–33], and discarding of unmethylated sites (<97.5 percentile of background coverage levels). Based on preliminary results, we also excluded 5 Mb of pericentromeric or 1 Mb of subtelomeric regions because polymorphic tandem repeats in these regions [34] were likely causing spurious inflation of significant associations. The final dataset comprised 4,532,060 SNPs and 4,544,738 methylation sites. Each methylation site, on average, comprised 2.91 CpGs and spanned 71.1 bp.

### Numbers and genomic distribution of meQTL effects

We used Matrix eQTL [35] to test all SNPs for association with methylation levels at every site. Our testing procedure accounted for covariates such as ancestry (four multi-dimensional scaling (MDS) dimensions), sex, sample batch and other laboratory assay variables. To prevent potential confounding effects from unmeasured sources of methylation variation, such as arises from cell type heterogeneity in blood, we also included the top seven principal components (PCs) from the methylation data (Fig. S1 in Additional file 1) as covariates in the association testing. To confirm that these PCs were not associated with genetic variation, we ran GWAS on the PC scores. Quantile-quantile (QQ) plots (Fig. S2 in Additional file 1) indicated that methylation variation captured by the PCs was not under detectable genetic control because no SNPs were significantly correlated with any PC.

**Fig. 1:**
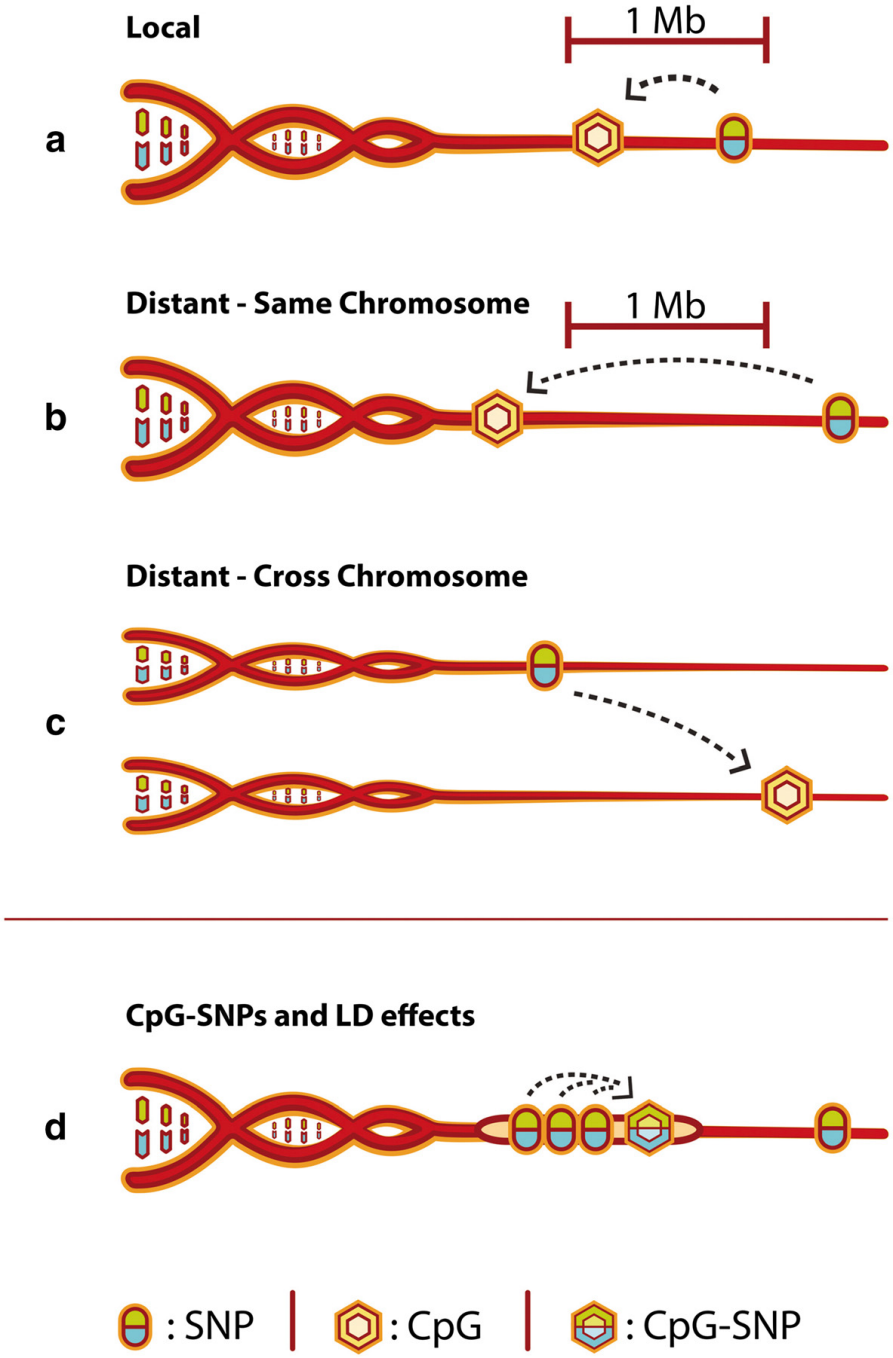
Schematic of possible meQTL effects. **a** Where the SNP and methylation site are within 1 Mb of each other, this is a “local” effect. All other effects are therefore “distant”, which is further sub-classified into same chromosome (**b**) and cross-chromosome (**c**) distant effects. As there are many millions of methylated sites in the genome, local SNPs with respect to one methylation site would be distant SNPs with respect to the vast majority of others. **d** The situation where a CpG-SNP affects methylation at a locus and thus causes a meQTL. Other SNPs in linkage disequilibrium (*LD*) with the CpG-SNP will also be associated with the CpG-SNP effect on methylation, and thus will appear to tag the meQTL

**Fig. 2:**
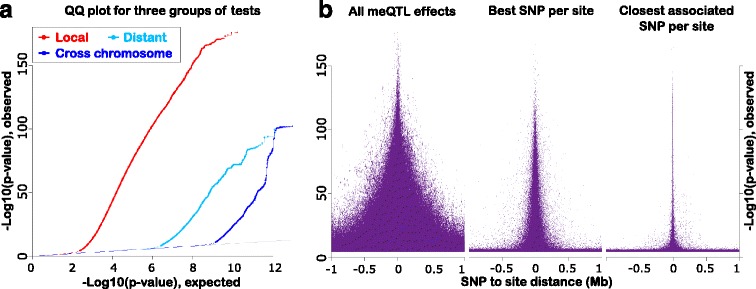
Quantile-quantile (QQ) and distance plots. **a** QQ plots for three different analysis categories. The *red line* indicates local meQTL effects (SNP and methylation site < 1 Mb apart), the *light blue line* indicates distant meQTL effects where SNP and site are on the same chromosome (SNP and methylation site >1 Mb apart), while the *dark blue line* indicates cross-chromosome meQTL effects. The *thin gray diagonal line* indicates the expectation under the null hypothesis. **b** Distance plots for meQTL effects, where the x-axis shows the distance between SNP and methylation site and the y-axis shows the negative logarithm (base 10) of the *p* values. Note the y-axis scale of the plots is identical to the QQ plots, allowing direct comparison. In the *left distance plot*, the distribution of all meQTL effects around methylation sites indicates that most effects occurred within a distance of 500 kb. The *central distance plot* shows just the most significant SNP per methylation site, indicating that this is typically close. The *right distance plot* displays only the closest SNP with FDR < 0.01 to each site. The narrow spike indicates that the phenomenon is highly localized. The difference between the left and right distance plots is primarily due to linkage disequilibrium between SNPs

Results from our primary analysis are summarized in Table 1. Following previous meQTL studies [15], tests were divided into “local” (SNP ≤ 1 Mb from methylation site) and “distant” (SNP > 1 Mb from site). Distant effects were further subdivided into same chromosome and cross-chromosome findings (Fig. 1). Associations were considered significant if they passed a stringent false discovery rate (FDR) threshold of 0.01, with the FDR calculated separately for each group of tests. Although the number of distant tests was much greater than the number of local tests (20,168 billion distant, versus 16 billion local), significant findings among local tests were 189,000-fold more common compared with all distant tests, or 5.8 million-fold more common compared with cross-chromosome tests (QQ plots in Fig. 2a). The preponderance of local effects is further explored in Fig. 2b–d. Here we show that the proportion of significant findings increased as distance between SNPs and their associated meQTLs diminished, indicating that genetically driven methylation is typically co-localized with the variation affecting it.

**Table 1:** Summary statistics for number of findings by analysis category

	Local (≤1 Mb)	Distant same chromosome (>1 Mb)	Cross-chromosome	Total
Part I: overall findings				
Number of tests (billions)	16.7	1140.2	19,440.1	20,597.0
Fraction of tests significant at FDR = 1 %	4 × 10^−3^	3.7 × 10^−7^	7 × 10^−10^	–
*P* value threshold for FDR = 1 %	4.05 × 10^−5^	3.74 × 10^−9^	7.03 × 10^−12^	–
Number of tests significant at FDR = 1 %	67,752,610	426,958	13,672	–
Number of unique SNPs with meQTLs	4,426,992 (97.68 %)	36,916 (0.81 %)	11,204 (0.25 %)	4,532,060
Unique methylation sites with meQTLs	683,152 (15.03 %)	3819 (0.08 %)	286 (0.01 %)	4,544,738
Part II: by methylation site features				
At MAF ≥ 0.05				
Sites (with meQTLs)	683,152	3,819	286	4,544,738
With CpG-SNPs	75 %	45 %	35 %	33 %
With other SNPs	12 %	18 %	20 %	30 %
Without SNPs	13 %	37 %	45 %	37 %

**Fig. 3:**
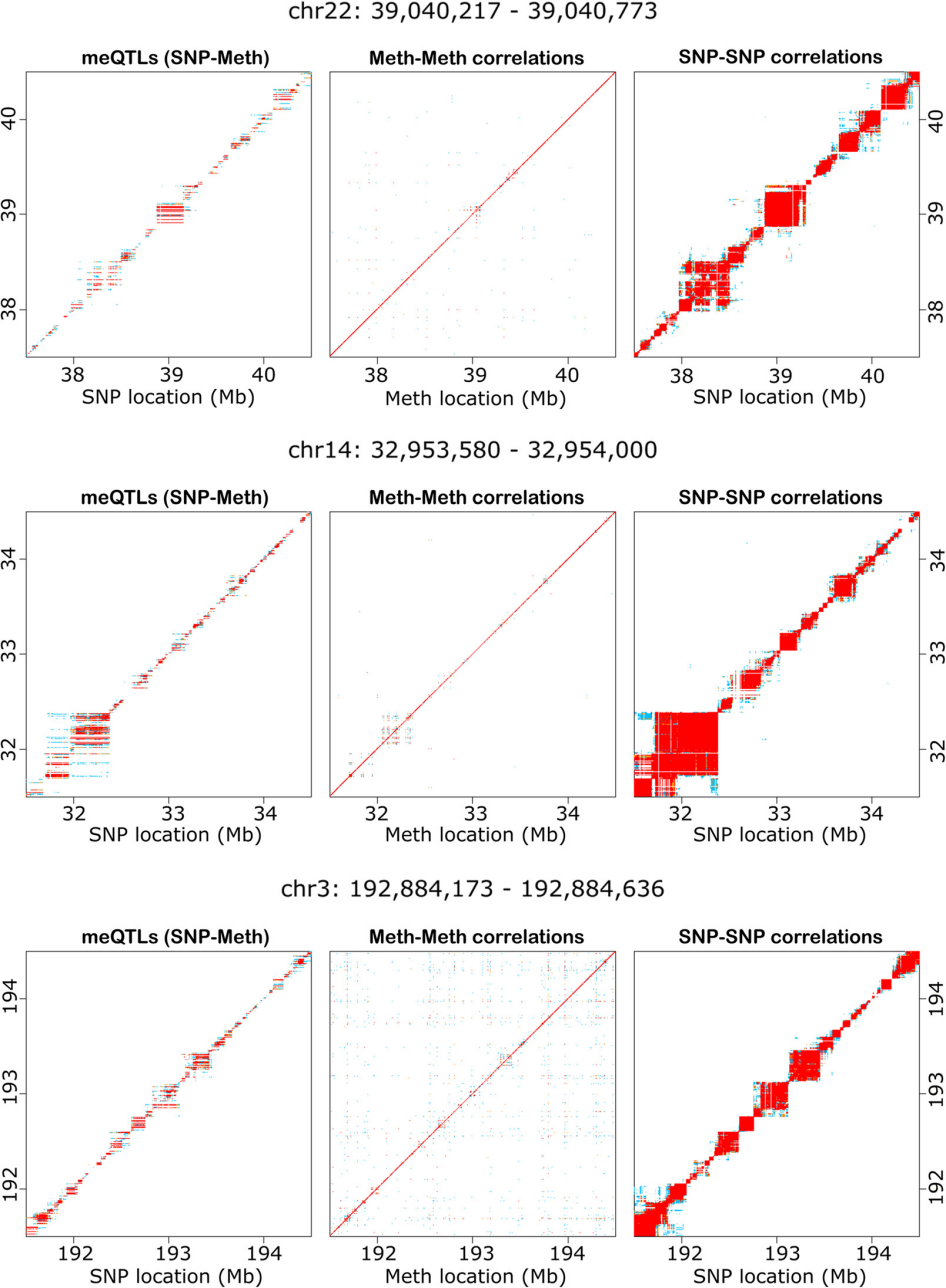
Methylation and genotype correlations by distance. We took the top three meQTL effects by *p* value in the study overall and plotted the distribution of methylation–methylation (*Meth–Meth*) correlation, meQTL effects (*SNP–Meth*) and SNP–SNP correlation around these findings. The *top panels* show several megabases around the top methylation site overall, chr22:39,040,217–39,040,773 (*p* = 8.08 × 10^−177^); the *middle panels* show the region around site chr14:32,953,580–32,954,000 (*p* = 1.10 × 10^−167^), while the *bottom panels* show the region around site chr3:192,884,173–192,884,636 (*p* = 5.71 × 10^−165^). Correlated blocks of SNPs in close proximity show association with the same methylation site(s). This leads to the horizontal “stripes” of significant meQTL associations. However, SNPs also tend to be associated with methylation levels at several sites on the same haplotype, leading to the two-dimensional patchwork of “striped squares” along the diagonal (see detail in *SNP-Meth* panels). This trend was observed universally in the genome, from small LD blocks measuring a few kilobases to very large regions, such as exists around the MHC on chromosome 6 (Supplementary Material in Additional file 1, p. 11). This serves to illustrate that while SNPs and their associated methylation sites tend to be co-localized, significant LD extending for many megabases can generate apparently long-range effects

To illustrate the genomic distribution of effects, plots of meQTLs by chromosome position are provided in the Supplementary Material in Additional file 1 (p. 6–27), with an interactive browser available at http://www.pharmacy.vcu.edu/biomarker/resources/supplementary. In Fig. 3, we show the pattern of results around the three most significant meQTLs by *p* value in our analysis. Fig. 3 also shows SNP–SNP and methylation–methylation correlations in the same distance windows. SNP–SNP correlations were much more extensive than those between methylation sites at similar physical distances. Blocks of SNPs in close proximity and in linkage disequilibrium (LD) showed association with the same methylation site(s), as expected. However, SNPs also tend to be associated with several methylation sites within the same LD block. These trends are observed throughout the genome, from LD blocks of a few kilobases to the largest block we observed, spanning over 20 Mb on chromosome 8 (Supplementary Material in Additional file 1, p. 13). These observations illustrate that LD extending for many megabases can spuriously suggest the presence of long-range effects. Furthermore, the overall number of significant tests is influenced by LD, whereby many highly correlated SNPs in close proximity tag each meQTL. This, coupled with the broad distribution of meQTLs throughout the genome and the relatively good statistical power provided by our study sample, explains our observation that 97.7 % of all SNPs tested were associated with methylation at one or more loci within 1 Mb. As such, the number of unique methylation sites under genetic control, rather than the number of significant SNP–methylation site associations, is arguably a better representation of the extent of genetic influence on the methylome. In our analysis, methylation levels at 15 % of sites were associated with one or more local SNPs. That is, 15 % of methylation sites were local meQTLs. These were 166-fold more common than all distant meQTLs and 2389-fold more common than cross-chromosome meQTLs.

### Replication of findings

**Fig. 4:**
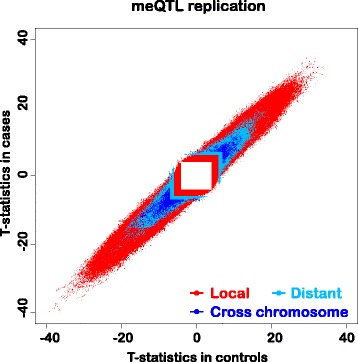
Replication plot showing agreement in test statistics for our primary sample of normal individuals (controls) versus a comparable sample, in terms of age, sex and number of subjects (N = 711; Table S1 in Additional file 1), of patients with a diagnosis of the mental disorder schizophrenia (cases). The distribution of effects along the diagonal indicates broad agreement for all three classes of effect

Our methylation data were obtained from control subjects who formed part of a larger study of the psychiatric disorder schizophrenia [27]. While methylation differences exist between cases and controls, we considered that the case sample would allow us to obtain a lower bound estimate of the replication rate of our findings. The case sample comprised 711 patients of similar age and sex distribution to the controls (Table S1 in Additional file 1). We calculated *p* values in the replication sample for methylation site–SNP pairs that passed 1 % FDR control in the primary analysis. We then computed the π1 statistic (estimate of the proportion of true positives in a *p* value distribution) [36] for these *p* values. The π1 statistic was 95 %, 98.7 %, and 99.3 % for local, distant same-chromosome, and cross-chromosome tests, respectively, indicating very high replication rates. Fig. 4 illustrates replication agreement.

### Genetically variable CpGs as a mechanism driving local meQTLs

SNPs that create or abolish CpGs (CpG-SNPs) have been suggested as genetic drivers of individual differences in methylation [37]. We hypothesized that this mechanism produced local meQTL effects in our study. Such meQTLs would result from CpG-SNPs at the methylation site of interest, with other local SNPs in LD with the CpG-SNP behaving as proxies of this phenomenon (Fig. 1d). We identified all CpG-SNPs in dbSNP 135 (30.1 % of SNPs) and then quantified how many methylation sites in our analysis encompassed a CpG-SNP, including a flanking region covering ±250 bp of the site boundary. This 250-bp window size represents the approximate maximum length of a sequenced fragment in our study, and therefore is the maximum distance from a locus at which a CpG-SNP could directly affect our methylation measure. In part II of Table 1, we show that 75 % of methylation sites under local genetic influence contain a CpG-SNP with MAF > 0.05, compared with 33 % of all sites genome-wide. This enrichment is also observed, albeit to a lesser extent, at lower CpG-SNP MAF thresholds (Table S2 in Additional file 1). Thus, most, but not all, local meQTLs could be explained by CpG-SNPs. By comparison, cross-chromosome meQTLs showed no enrichment for CpG-SNPs.

### Bioinformatics analysis of meQTL findings

We tested if our meQTLs (i.e., those methylation sites with significant SNP associations) were enriched in several categories of genomic annotations. We performed our bioinformatics analyses in two phases. In the first phase, we looked at aggregate annotation categories (e.g., genes, transcription factor binding sites), with *p* values generated via up to four million permutations. We initially focused on local findings, first examining enrichment among all local meQTLs and then split our data to characterize enrichment patterns for meQTLs with CpG-SNPs and those without. In the second bioinformatics analysis phase, we looked at specific annotations in more detail, reducing the number of permutations to one-tenth of those carried out in the first phase and including FDR control due to the larger number of tests. Initial phase 1 analysis found some enrichment of genomic duplications, copy number variants, and pseudogenes among meQTL findings. This was modest for local effects but substantially greater for cross-chromosome compared with local meQTLs (Table S3 in Additional file 1). We eliminated methylation sites overlapping these features and re-analyzed the remainder.

#### Initial genomic annotation analysis of all local meQTLs

**Fig. 5:**
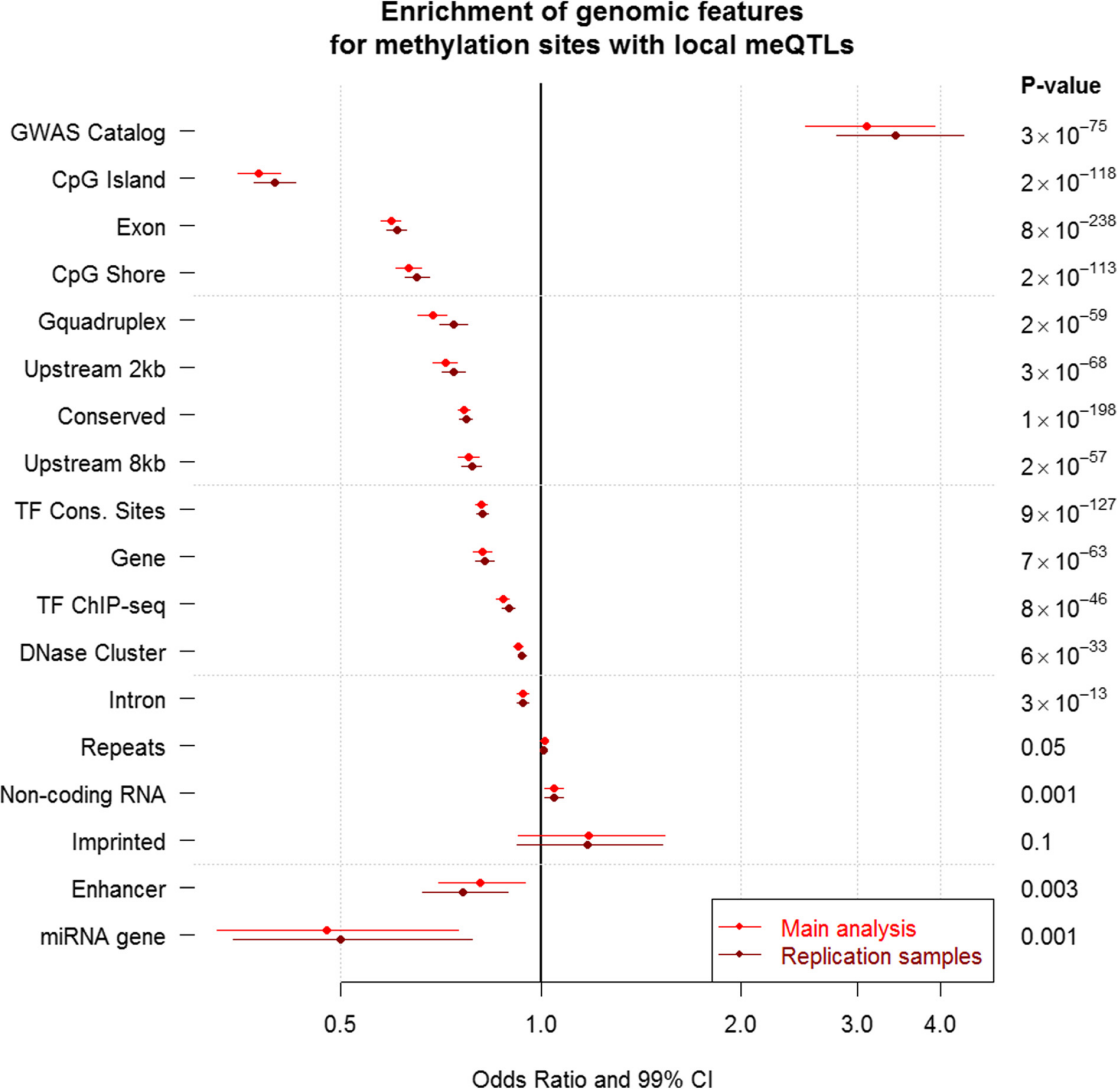
Enrichment of all local meQTLs in broad genomic annotation categories. The x-axis is the odds ratio for enrichment and 99 % confidence intervals (*CI*) are provided for all data points. We show enrichment in both our primary sample (main analysis) and in the replication sample. In all instances, significance was calculated based on more than four million permutations. Annotation categories are as follows: (1) GWAS catalog represents hits from the National Human Genome Research Institute (NHGRI) GWAS catalog; (2) CpG islands; (3) exons; (4) CpG shores, defined as 2 kb flanking a CpG island; (5) G-quadruplexes; (6) narrow promoter region 2 kb upstream from transcription start; (7) conserved across 29 eutherian mammals; (8) broad promoter region 8 kb upstream from transcription start; (9) conserved transcription factor (*TF*) recognition sequences; (10) RefSeq genes; (11) ENCODE transcription factor binding data from chromatin immunoprecipitation sequencing (ChIP-seq) experiments; (12) DNaseI hypersensitive regions; (13) introns; (14) repetitive elements; (15) long non-coding RNAs; (16) known imprinted genes; (17) VISTA enhancers; and (18) microRNA genes

The results for the phase 1 analysis of all local meQTLs are summarized in Fig. 5 (details are provided in Table S3 in Additional file 1). To estimate enrichments that could be observed by chance, we used the annotations for all 4.5 million methylation sites that we assayed as our background set in the permutation analysis. We found that local meQTLs were significantly less likely to overlap with almost all functional features tested, including CpG islands, genes, promoter regions, DNaseI hypersensitive regions, etc., compared with sites that were not meQTLs. However, local meQTLs were significantly more likely to overlap with GWAS hits from the National Human Genome Research Institute (NHGRI) GWAS catalog (odds ratio = 3.09, *p* = 2.6 × 10^−75^). It should be noted that in this instance we consider a very specific overlap, i.e., the GWAS catalog SNP is within the boundary of the methylation site. This finding was also observed in our replication sample (odds ratio (OR) = 3.4, *p* = 4.8 × 10^−79^). Of the 393 methylation sites under local genetic control overlapping the GWAS catalog in our main analysis (Table S4 in Additional file 1), 366 encompassed a CpG-SNP with MAF > 0.05 while 387 encompassed a CpG-SNP with MAF > 0.01. In our phase 2 analysis, we examined the specific phenotypes contributing to the NHGRI GWAS catalog overlap with all local meQTLs. We show results for the 21 phenotypes passing FDR < 0.01 in Table 2. A common theme was not apparent, with genetic effects on methylation appearing to influence traits such as body morphology and cardiovascular, autoimmune and psychiatric disorders, amongst others.

**Table 2:** Traits and diseases from the NHGRI GWAS catalog with associated loci enriched for local meQTLs

Phenotype	N annot	Overlap	Fisher PV	OR	Perm Z	Perm PV	Perm QV
Bone mineral density	25	15	1.58E-07	9.03	6.530	4.40E-10	1.07E-07
Breast cancer	34	17	8.28E-07	6.02	5.878	2.51E-08	3.05E-06
Blood pressure	9	7	3.28E-05	21.06	5.551	1.63E-07	1.31E-05
Height	90	31	1.25E-06	3.16	5.499	2.16E-07	1.31E-05
Major depressive disorder	14	9	2.44E-05	10.84	5.452	2.80E-07	1.36E-05
Non-alcoholic fatty liver disease	7	6	5.13E-05	36.10	5.413	3.47E-07	1.41E-05
Alzheimer’s disease	17	10	2.54E-05	8.60	5.203	1.06E-06	3.67E-05
Rheumatoid arthritis	37	16	1.84E-05	4.59	5.167	1.27E-06	3.86E-05
Hypertension	10	7	9.59E-05	14.05	5.010	2.82E-06	7.62E-05
QRS duration	8	6	1.80E-04	18.06	4.911	4.62E-06	1.08E-04
Crohn’s disease	42	17	2.89E-05	4.09	4.900	4.89E-06	1.08E-04
Pulmonary function decline	6	5	3.10E-04	30.10	4.803	7.79E-06	1.58E-04
IgG glycosylation	70	23	8.67E-05	2.95	4.409	4.80E-05	8.98E-04
Systemic lupus erythematosus	15	8	4.25E-04	6.88	4.199	1.18E-04	1.93E-03
Bone mineral density — spine	5	4	1.82E-03	24.08	4.198	1.19E-04	1.93E-03
Hip geometry	5	4	1.82E-03	24.08	4.173	1.32E-04	2.01E-03
Autism spectrum, attention deficit-hyperactivity, bipolar and major depressive disorders and schizophrenia combined	19	9	5.72E-04	5.42	4.103	1.77E-04	2.52E-03
Mean corpuscular hemoglobin	5	4	1.82E-03	24.08	4.084	1.90E-04	2.57E-03
Tuberculosis	8	5	2.25E-03	10.03	4.031	2.36E-04	3.02E-03
Bipolar disorder and schizophrenia	24	10	9.86E-04	4.30	3.799	5.85E-04	7.11E-03
Obesity-related traits	165	40	7.21E-04	1.93	3.709	8.22E-04	9.52E-03

We examined 494,420 meQTLs out of 3,470,923 methylation sites, after removal of sites in regions flagged as copy number variants, genomic duplications or pseudogenes. We consider local meQTLs and do not stratify by presence or absence of CpG-SNPs at the meQTL site. “N annot” is the number of loci in the NHGRI GWAS catalog for that trait or disease, “Overlap” is the number of GWAS loci that overlap with a local meQTL, “Fisher PV” is the *p* value from the Fisher exact test of enrichment, “OR” is the odds ratio, “Perm Z” is the Z-statistic of the permutation test, “Perm PV” is the *p* value from >300,000 permutations, “Perm QV” is the *q* value of the permutation test. Only findings passing FDR control with *q* value < 0.01 are shown

#### Genomic annotation analysis of local meQTLs with and without CpG-SNPs

Common (MAF > 0.05) CpG-SNPs were present at most (75 %), but not all, local meQTLs. This implies that different mechanisms are operating to influence methylation at meQTLs without CpG-SNPs. To compare patterns of genomic features for local meQTLs with CpG-SNPs and those without, we reran our phase 1 analysis stratifying by common CpG-SNP (MAF > 0.05) presence or absence. Thus, in this analysis, local meQTLs with CpG-SNPs were compared with a background set of all methylation sites with CpG-SNPs (out of the 4.5 million assayed in our study). Similarly, local meQTLs without CpG-SNPs were compared with all methylation sites lacking CpG-SNPs.

We found that local meQTLs with common CpG-SNPs showed similar patterns to our observations for all local meQTLs, except that they were much less enriched for G-quadruplexes (Table S3 in Additional file 1). For meQTLs lacking common CpG-SNPs, these were significantly enriched for transcription factor (TF) binding sites (OR = 1.11, *p* = 7.24 × 10^−18^) and DNase clusters (OR = 1.12, *p* = 1.11 × 10^−37^). Notably, these findings became more pronounced when we excluded sites with any CpG-SNP, regardless of MAF (TF binding sites OR = 1.25, *p* = 3.56 × 10^−39^; DNase clusters OR = 1.24, *p* = 1.59 × 10^−59^). These findings were also observed in the replication sample, with similar ORs and significance levels (Table S3 in Additional file 1). To identify the specific TFs accounting for the observed aggregate enrichment at local meQTLs, we obtained the individual genome-wide binding profiles for more than 200 TFs from ENCODE [38]. We then analyzed each TF binding profile separately, rather than in aggregate as above, and results are shown in Table S5 in Additional file 1. The individual TFs that displayed the greatest enrichment and passed FDR < 0.01 were ZBTB33 (OR = 3.85, *p* = 9.67 × 10^−18^), p300 (OR = 3.06, *p* = 9.67 × 10^−18^) and TR4 (OR = 2.84, *p* = 9.67 × 10^−18^). As shown in Table S5 in Additional file 1, binding sites for 66 unique TFs showed enrichment for local meQTLs. This large number may be partly explained by the tendency of TF binding profiles to overlap in regions critical for transcriptional regulation [38]. Nevertheless, our finding suggests that genetic influence on TF binding can have substantial influence on local methylation levels and that this mechanism is potentially applicable to a large number TFs.

#### Enrichment analysis of local meQTLs in Roadmap Epigenomics chromatin states

**Fig. 6:**
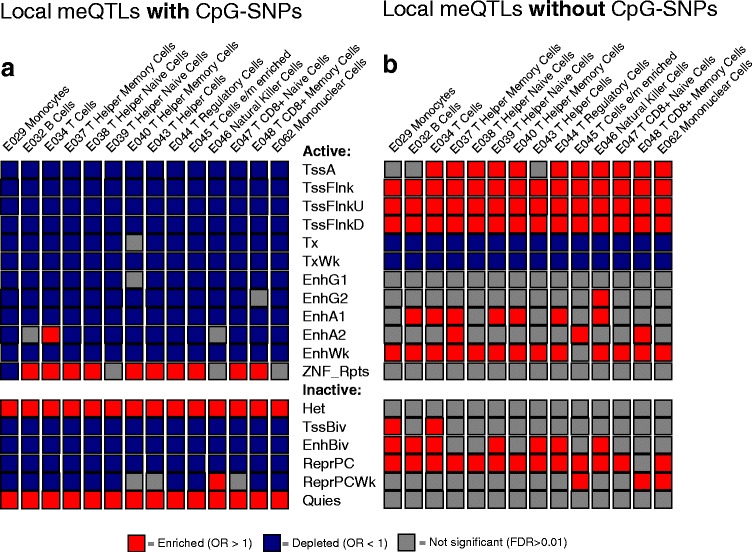
Enrichment/depletion of local meQTLs in different chromatin states. Chromatin state classification used an 18 state model from the Roadmap Epigenomics Consortium [18] (http://egg2.wustl.edu/roadmap/web_portal/chr_state_learning.html#exp_18state) for peripheral blood cells. Testing was conducted via permutation and details (*p* values, test statistics, etc.) are provided in Table S6 in Additional file 1. **a**, **b** Enrichment for local meQTLs with and without CpG-SNPs, respectively. Effect direction is color-coded, with significant (*q* value < 0.01) enrichment (OR > 1) shown in *red* and depletion (OR < 1) shown in *blue*. Roadmap chromatin states are broadly categorized as active and inactive [18]. Active chromatin states are as follows: active transcription start site (*TssA*), flanking transcription start site (*TssFlnk*), upstream flanking transcription start site (*TssFlnkU*), downstream flanking transcription start site (*TssFlnkD*), strong transcription (*Tx*), weak transcription (*TxWk*), genic enhancer (*EnhG1* and *EnhG2*), active enhancer (*EnhA1* and *EnhA2*), weak enhancer (*EnhWk*), zinc finger genes and repeats (*ZNF_Rpts*). Inactive chromatin classes are as follows: heterochromatin (*Het*), bivalent/poised transcription start site (*TssBiv*), bivalent enhancer (*EnhBiv*), repressed polycomb (*RepPC*), weak repressed polycomb (*RepPCWk*), and quiescent (*Quies*)

In our phase 1 analysis, the presence or absence of CpG-SNPs differentiated local meQTLs with respect to enrichment in TF binding sites and DNase clusters. This suggested that meQTLs without CpG-SNPs were more likely to occur in regions of active chromatin. To study this in more detail, we looked for local meQTL enrichment in genomic regions classified into specific chromatin states. Local meQTLs were divided according to presence or absence of CpG-SNPs (any MAF). Chromatin state classification was according to an 18 state model from the Roadmap Epigenomics Consortium [18] for peripheral blood cells, of which there was information on 14 distinct cell types. Results are summarized in Fig. 6 and full details are provided in Table S6 in Additional file 1). A very distinct pattern of results was obtained for local meQTLs with CpG-SNPs, which were strongly enriched in heterochromatin and quiescent regions, in addition to regions harboring zinc finger protein genes and repeats, while being significantly depleted in other chromatin states. In contrast, meQTLs without CpG-SNPs were enriched around transcription start sites and enhancers. These were also enriched in some inactive chromatin regions, notably in repressed polycomb regions. There was strong agreement in the pattern of enrichment across cell types, indicating a broad consistency in chromatin activity patterns among the major classes of peripheral blood cells studied.

#### Secondary analysis of local meQTLs overlapping the NHGRI GWAS catalog

Of the 393 local meQTLs that overlapped the NHGRI GWAS catalog, the vast majority (>93 %) encompassed a common CpG-SNP (MAF > 0.05), while practically all (>98 %) encompassed a CpG-SNP with MAF > 0.01. However, our chromatin state analysis showed that meQTLs with CpG-SNPs were much more likely to occur in quiescent or heterochromatin regions. Given that most local meQTLs with CpG-SNPs are in regions unlikely to affect phenotype, we speculated that those overlapping the NHGRI GWAS catalog were in some way distinct. We therefore compared the local meQTLs with CpG-SNPs overlapping the NHGRI GWAS catalog with all local meQTLs with CpG-SNPs. Due to the smaller numbers of meQTLs being tested, this analysis had lower power relative to those above and none of the findings passed our stringent threshold of FDR < 0.01. However, five tests did pass a more typical FDR < 0.05 threshold (Table S7 in Additional file 1). Specifically, those local meQTLs with CpG-SNPs overlapping the GWAS catalog were less likely to occur in quiescent and heterochromain regions and more likely to occur in regions of active chromatin, specifically active enhancers (maximum OR = 2.2, *p* = 1.9 × 10^−4^) than all local meQTLs with CpG-SNPs. These results suggest that meQTLs with CpG-SNPs in regions of active chromatin associated with disease could be high priority targets for further characterization in disease studies.

#### Genomic annotation analysis of SNPs and cross-chromosome meQTLs

For distant meQTLs, to prevent possible contamination from local effects in regions where LD exceeds 1 Mb, we focused on cross-chromosome findings. Cross-chromosome meQTLs (i.e., methylation sites associated with SNPs on other chromosomes) were significantly more likely to overlap with exons (OR = 5.24, *p* = 3.39 × 10^−18^). This association was also apparent in the replication sample (OR = 4.91, *p* = 7.78 × 10^−19^), suggesting that some cross-chromosome findings may have functional relevance (Table S3 in Additional file 1). Unlike local meQTLs, however, there was no enrichment for cross-chromosome meQTLs in the NHGRI GWAS catalog, suggesting any functional effects they may have are benign.

Phase 1 annotation analysis for local and distant SNP effects are also included in Table S3 in Additional file 1 for completeness. In these SNP analyses, only common SNPs (MAF > 0.05) were included as the background set because only common SNPs were included in our study. The local SNP findings showed no enrichment for most features tested. However, the vast majority of SNPs were associated with one or more local meQTLs so the discriminatory power of this local analysis is limited. Finally, cross-chromosome SNP findings were not significantly enriched for any phase 1 annotation category.

## Discussion

All previous studies of meQTLs found abundant local effects in all tissues analyzed [9, 15, 17]. Our findings suggest that (1) any site under local genetic influence was typically associated with a SNP in very close proximity, (2) local effects tracked LD, and (3) the majority of sites under local genetic control included a CpG-SNP within their boundary. Distant effects showed no such enrichment. Taken together, these observations suggest that most meQTLs involve processes that are entirely dependent on local sequence context, or are LD proxies of such processes, consistent with previous studies of allele-specific methylation [39, 40]. Previous studies have noted the importance of CpG-SNPs as mediating genome–epigenome interaction [37, 40] and our study provides extensive quantitative evidence to support this.

Several different processes appear to be working to cause meQTLs. For those meQTLs overlapping CpG-SNPs, absence of the CpG sequence in some individuals will clearly prevent methylation. However, in others the presence of a CpG means only that it *can* be methylated, not necessarily that it *will* be. Methylation of the CpG in the cell type of interest is necessary for the meQTL effect to be observed. In our data, we observed enrichment of meQTLs with CpG-SNPs in quiescent and facultative heterochromatin regions, both of which are heavily methylated (>75 %) [18]. This suggests that, for this class of meQTLs, we are simply picking up the sequence differences at polymorphic CpGs between individuals in heavily methylated regions. Quiescent and heterochromatin regions are also transcriptionally inactive. A small number of previous studies have examined meQTLs and expression QTLs (eQTLs) in the same tissue and observed very low concordance. For example, Gibbs et al*.* [15] found that only 4.8 % of significant meQTLs were also an eQTL. The fact that most of our meQTLs included CpG-SNPs and these were in transcriptionally inactive chromatin regions could be one factor in explaining the low concordance between meQTLs and eQTLs.

One expectation based on these observations could be that meQTLs with CpG-SNPs typically have no effect on phenotype. However, this is somewhat incongruent with our finding that local meQTLs were enriched in loci from the NHGRI GWAS catalog and almost all sites overlapping the catalog encompassed CpG-SNPs. For example, the most significant site among the overlap spanned 157 bp at the *TCF7L2* locus on chromosome 10 (114,753,967–114,754,123) and the associated SNP was rs34872471 (*p* = 1.4 × 10^−126^). The CpG-SNP within the boundary of this site, rs7901695 (chr10:114,754,088), was associated at a similar level of significance (*p* = 2.1 × 10^−114^). It is considered to be a confirmed susceptibility variant in diabetes [41, 42], with over 30 publications at time of writing linking it to the disease. Mutation of CpGs at certain critical loci is considered to be an important etiological mechanism for complex diseases [37]. We observed almost 400 findings that overlapped with the GWAS catalog, including associations with many different disease genes and disorders, suggesting that meQTLs with CpG-SNPs overlapping the GWAS catalog were distinct in some way. Our analysis showed that these meQTLs were more likely to be in active chromatin, with our most significant enrichment in active enhancers. This echoes previous studies showing enrichment of disease- or trait-associated variants in specific chromatin states [43] or accessible regions [44]. Recent findings from the Roadmap Epigenomics Consortium also indicated enrichment of disease-associated variants in active enhancers, most notably those associated with H3K4me1 and H3K27ac histone marks [18]. Based on these findings, we suggest that local meQTLs with CpG-SNPs in active chromatin regions in the relevant tissue should be priority targets for functional follow-up in disease mapping studies. Our meQTLs from NGS are typically small, spanning only 2.9 CpGs and approximately 70 bp on average. Thus, they could enable fine-scale prioritization of specific variants from the large regions implicated in GWAS.

Where local meQTLs lack CpG-SNPs, alternative mechanistic explanations must be considered. It is known that non-CpG variants influence the binding of *cis*-acting factors that, in turn, affect methylation levels. For example, binding of Sp1 serves to prevent methylation at some CpGs in promoter regions [45]. We observed that local meQTLs lacking CpG-SNPs were more likely to overlap TF binding sites, in agreement with this mechanistic explanation. However, the extent of this phenomenon is greater than perhaps previously understood, with a broad range of TF binding profiles showing significant enrichment for meQTLs without CpG-SNPs. We observed that meQTLs without CpG-SNPs were enriched in active regions of chromatin, particularly at the transcription start site and flanking regions. This is congruent with binding of TFs being the underlying mechanism. However, we also observed meQTLs without CpG-SNPs to be enriched in some inactive regions, particularly repressed polycomb (RepPC) regions. Therefore, our results suggest that genetic differences that affect binding of several different classes of chromatin binding factors are an important influence on the methylome.

## Conclusions

Our use of high-density genome-wide SNP genotyping and imputation based on 1000 Genomes data enabled us to capture much of the common SNP variants in this sample. Our use of NGS to assay DNA methylation enabled us to assess most methylated CpGs in the non-repetitive portion of the genome. We confirmed that genetic influence on methylation is a pervasive phenomenon throughout the genome and, for the most part, highly localized in its effect. Our findings suggest that several mechanisms can generate meQTLs. These include CpG-SNPs and variants that interfere with chromatin binding for several classes of proteins. The very large number of local meQTL effects attributable to CpG-SNPs, coupled with the fact that they typically occur in non-functional regions of the genome, suggests that most have little phenotypic consequence. However, the observed enrichment of meQTLs in disease or trait-associated regions from the NHGRI GWAS catalog indicated that a small portion of CpG-SNPs have arisen in regions of the genome where they may exert significant influence on phenotype. Integration of meQTLs with other data, such as RoadMap Epigenomics, could aid in functional interpretation of SNPs identified in disease GWAS. We therefore provide all our meQTL findings, including positional information and association statistics, in Table S8 in Additional file 1.

## Materials and methods

### Ethics

All procedures were approved by the institutional review board at the Karolinska Institutet, Stockholm, Sweden (IRB/KI 04/-499/4) and further locally approved by the Virginia Commonwealth University institutional review board (IRB#. HM12499). Subjects provided written informed consent (or legal guardian consent and subject assent). All experimental methods comply with the Helsinki Declaration.

### Subjects and biological sampling

Subjects were controls collected as part of a larger project entitled “*A Large-Scale Schizophrenia Association Study in Sweden*”. This overarching project [30, 46, 47] aims at understanding the etiology of schizophrenia and bipolar disorder plus their clinical and epidemiological correlates. Peripheral blood was donated at the local medical facilities of the participants. DNA was extracted from EDTA blood using the Gentra Puregene kit for automated extraction with the Autopure LS robot (Qiagen).

### Genome-wide SNP genotyping, quality control, and imputation

Genotyping was carried out as described previously [29, 30]. Briefly, subjects were genotyped with Affymetrix genome-wide SNP Arrays 5.0 or 6.0, or Illumina OmniExpress. All genotyping was conducted at the Broad Institute of Harvard and the Massachusetts Institute of Technology. Genotypes were called using the Birdsuite (Affymetrix) or BeadStudio (IIllumina). QC exclusionary measures for subjects were: genotype call rates <95 %; ancestry outliers via multidimensional scaling; a randomly selected member of any pair of subjects with high relatedness (*π* > 0.20); and suspected sample error or contamination indicated by high heterozygosity or indeterminate genetic sex. SNPs were excluded for marked departure from Hardy–Weinberg equilibrium (*p* < 1 × 10^−6^), low minor allele frequencies (<1 %), and non-random genotyping failure, inferred from the flanking haplotype background using the PLINK ‘mishap’ test (*p* < 1 × 10^−10^). Plate-based associations of *p* < 1 × 10^−6^ were taken as evidence of non-random genotyping failure, based on a comparison of allele frequency of each plate to all others and were removed on a plate-by-plate basis. To enhance coverage, we imputed SNPs from 1000 Genomes data (phase I version 3) using Minimac, after phasing genotypes with MACH 1.0 [28]. After selecting on MAF > 0.05 and imputation quality measure *r*^2^ > 0.5, a total of 4,761,800 imputed and genotyped SNPs were available for meQTL association testing.

### MBD-seq

Our methods and analysis pipeline make use of MBD protein-based enrichment and sequencing (MBD-seq) as described previously [26, 27, 31]. Briefly, genomic DNA was sheared to median fragment size = 125 bp (Covaris E210) and the methylated portion captured using MethylMiner (Invitrogen), followed by elution in 500 mM NaCl. Methylated DNA fragments were sequenced (SOLiD, Life Technologies) using standard multiplexed single end (50 bp) methods. The SOLiD system aligns in color space and uses two-base encoding [48], producing two ‘color calls’ for each base. After deleting poor quality reads (>2 missing color calls), we obtained an average of 67.3 million (SD = 26.9 million) total reads per sample. This exceeds the recommended 30–60 million reads required for genome-wide methylation analysis via enrichment-based methods [49, 50]. The mean quality value (QV = −10log_10_(*p*) with *p* being the probability of an error) per color call was 21.4 (SD = 1.1). Reads were aligned to the human reference genome (hg19/GRCh37) using BioScope 1.2 (Life Technologies). The percentage of mapped reads was 69.2 % (SD = 6.2). We deleted reads with multiple poor quality alignments and high copy number duplicate reads were collapsed to single reads. This led to the elimination of 32.1 % of the mapped reads. After all QC, we obtained, on average, 31.6 million reads per subject (SD = 13.4 million).

### MBD-seq methylation measures

Locus-specific methylation measures were obtained by summing the number of fragments expected to cover each CpG. Note that methylation of any CpG in a DNA fragment, not just the sequenced 50 bp, could lead to its capture by MBD protein. Hence we define locus-specific methylation measures as the expected number of DNA fragments covering each CpG [33]. Specifically, fragments whose sequenced part is covering a CpG contribute a unit to its methylation measure. For some fragments, it is not known whether they cover a given CpG, so their contribution to the methylation measure is set to the estimated probability that the fragment covers the CpG. This probability is a function of fragment size distribution. We estimated the fragment size distribution empirically from the distribution of reads around isolated CpGs [33]. The calculation of methylation measures can be schematically expressed as:

$$ Methylation\operatorname{} Measur{e}_{CpG}={\displaystyle \sum_{fragments\  near\ CpG}}\hat{P}\left( Fragment\  covers\  the\ CpG\right). $$

The average number of fragments covering a particular CpG depends not only on the methylation status of that site but also on the number of methylated CpGs in the region [20]. To make coverage estimates more comparable across sites and improve the correlation with actual methylation levels, coverage estimates can be further normalized using the local CpG density as a proxy for the number of methylated CpGs in the region [50, 51]. However, our meQTL analyses essentially involve the calculation of correlations between SNPs and quantile normalized methylation levels. As these correlations are not affected by monotone transformations of methylation levels, for sake of simplicity we did not use such a normalization step.

Thirty-six percent (10.5 million) of all ~27 million autosomal CpGs in the reference genome (hg19/GRCh37) were eliminated because of predicted alignment problems, as observed in an in silico analysis [31]. The majority (71.8 %) of these were in regions flagged as repetitive elements by RepeatMasker. To reduce the size of the data set, the remaining ~16 million CpGs were adaptively combined by collapsing highly inter-correlated coverage estimates at adjacent CpG sites into a single mean coverage estimate [31, 52]. Prior to association testing, we dropped sites with very low levels of coverage as these were likely unmethylated (<97.5 % of background coverage at non-CpG sites, where the latter are defined as loci with no CpGs within 400 bp).

We previously reported in-depth quality metrics for this methylation dataset [31]. Briefly, the ratio between the median coverage at a CpG, i.e., the methylation signal, versus coverage at a non-CpG, i.e., the background noise, is >40. This indicates the signal to noise ratio is high. Second, in an analysis of 73 technical replicates, we observed a median correlation of 0.92 for genome-wide methylation measures between replicates. This indicates that our MDB-seq measures are robust and reproducible. Several studies have compared MBD-seq quality and genome-wide coverage with other methods [20, 22, 25]. Enrichment-based sequencing methods, such as MBD-seq and MeDIP, are cheaper than WGBS and provide better genome-wide coverage than microarrays [13]. By measuring the relative enrichment of methylated DNA rather than absolute levels, enrichment-based methods are somewhat less accurate than Infinium arrays or bisulfite sequencing for quantifying DNA methylation levels in partially methylated regions. Enrichment-based methods can, however, distinguish between methylated and unmethylated regions almost as precisely as bisulfite sequencing [49]. Compared with MeDIP, MBD-seq is less noisy (picks up fewer sporadically methylated sequences) but only assays methylation at CpGs [21]. Furthermore, standard MBD-seq preferentially assays CpG-dense regions [25]. To improve methylome-wide coverage, we used an existing protocol variant that increases the relative number of fragments from CpG-poor regions by eluting the captured methylated fraction with 0.5 M NaCl [31].

### Association analyses and FDR control

To test for association between genotype and methylation measurements (each SNP versus each methylation site) we used Matrix eQTL [35], a computationally efficient analysis tool implemented in R (http://www.r-project.org/). Methylation values were first corrected using the inverse quantile normal transformation of the ranked values. This robust approach greatly reduces the effect of outliers, while retaining more power than rank-based procedures [53]. To eliminate possible technical artifacts, lab variables and sample batch were included as covariates in the linear regression model. We also performed a principal components analysis of the methylation data to eliminate unmeasured confounders [32]. As is true for most tissues, blood consists of a variety of cell types. By using whole blood we study an “average” methylation pattern. This can produce false positives if two conditions hold simultaneously: (1) the relative abundance of common cell types is correlated with the outcome variable of interest, and (2) methylation patterns of these cell types differ. Ideally, we would have methylation data obtained from separated blood cells [54] to identify sites that are at risk for being false positives. However, principal components analysis provides an alternative solution [54–56]. Subjects with similar cell type compositions will have more similar multi-locus methylation patterns and these patterns will be captured and regressed out through the PCs. Based on a scree plot (Fig. S1 in Additional file 1), the first seven PCs were selected for inclusion as covariates in our meQTL analysis.

For the SNP data, we used four MDS dimensions to control for ancestry, as used in the original report of GWAS in this sample [29]. Due to the large size of the data sets, methylation data and SNP genotypes were split by chromosome and Matrix eQTL was applied separately for each pair of chromosomes. We controlled the FDR [57] at 1 %. Separate FDR calculations for local, distant same chromosome and cross-chromosome tests were performed to account for variations in the proportion of null tests across these scenarios and ensure the FDR was efficiently controlled at the desired 1 % level in all cases. Namely, different *p* value thresholds were used for these three groups of test to ensure at most 1 % of discoveries were false in each group. Within each group FDR was calculated using the standard Benjamini–Hochberg procedure, which is known to be more conservative than other common FDR procedures (as it assumes that the fraction of non-null tests is small or zero).

The Benjamini–Hochberg FDR control procedure works as follows. First, the *p* values in each group were ordered in increasing order: *P*_(1)_, *P*_(2)_, …, *P*_(*m*)_. Next, the maximum *k* is selected such that ${P}_{(k)}\le \frac{k}{m}\alpha$. The tests with smallest *k p* values are then declared to have FDR below *α*. In our case *α* = 0.01 (i.e., 1 %).

### Bioinformatics analyses

Annotation tracks for the first analysis phase were obtained via UCSC genome browser download for human genome build hg19 and dbSNP version 135. We selected the following tracks for testing: (1) RefSeq genes and used gene positional information to calculate (2) exon, (3) intron, (4) promoter region 2 kb upstream and (5) promoter region 8 kb from transcription start site annotations. We also selected (6) conservation based on similarity between 29 eutherian mammals, (7) CpG islands (defined as GC content of ≥50 % or greater, length 200 bp, CpG ratio 0.6), (8) CpG shores (2 kb regions flanking a CpG island [19]), (9) repetitive elements from RepeatMasker (http://www.repeatmasker.org/), (10) conserved TF binding sequences between humans and rodents as provided in TransFac version 7.0, (11) clustered ENCODE TF binding sites mapped via chromatin immunoprecipitation sequencing (ChIP-seq) [58, 59], (12) DNase clusters from the University of Washington DNaseI hypersensitivity submission to ENCODE, (13) long non-coding RNAs from Gencode version 18, (14) VISTA enhancers [60], (15) microRNA genes, (16) G-quadruplexes [61], (17) known imprinted genes (http://www.geneimprint.com), and (18) NHGRI GWAS catalog hits (http://www.genome.gov/gwastudies) [62].

For each methylation site or SNP, we determined overlap with each annotation category and then compared these with the set of sites/SNPs with detected meQTLs. The significance of the enrichment of sites/SNPs in each category with sites/SNPs with meQTLs was initially assessed using Fisher’s exact test (R function “fisher.test”). However, this test requires independence of observations and the neighboring sites/SNPs are likely to be correlated and thus violate this assumption. For proper assessment of statistical significance we performed permutation analysis based on circular shifts as they preserve local dependence of sites/SNPs. A circular shift permutation analysis was conducted in the following way. First, the methylation sites are ordered by genomic location. Then, the annotation tracks for the sites are shifted by B positions forward, with annotations for the last B sites assigned to the first B sites. Then, overlap of meQTLs with annotation tracks for the shifted annotation is calculated for all values of the offset B, except those shifting the original annotation by less than 1 % of the total number of sites in either direction. The overlap of meQTLs with annotation tracks under no permutation is then compared with those under circular shift permutations.

We observed that the permutation distribution of the overlap counts was very close to Gaussian in each of our tests (data not shown). For each test we fitted the normal distribution to the set of overlap counts and calculated the z-score for the overlap count observed for the original non-permuted data. The permutation *p* values were then calculated from the corresponding z-scores.

In the phase 2 bioinformatics analyses, we used a database of genomic annotations assembled in the GenomeRunner project [63] to examine enrichment of meQTLs in selected annotation classes. These included (1) individual disease-associated SNP sets from the manually curated NHGRI Catalog of Published Genome-Wide Association Studies [62] (accessed on 16 March 2015), (2) cell type-specific binding sites of individual TFs from the ENCODE [64] (accessed on 1 December 2014) and (3) cell type-specific chromatin states according to the 18-state model from the Roadmap Epigenomics Consortium [18] (accessed on 18 March 2015). We further tested the enrichment of 393 meQTLs overlapping disease/trait-associated SNPs cataloged by NHGRI in these cell type-specific 18 chromatin states. We used only data for primary cell lines from peripheral blood (E029 monocytes; E032 B cells; E034 T cells; E037 T helper memory cells; E038 T helper naive cells; E039 T helper naive cells; E040 T helper memory cells; E043 T helper cells; E044 T regulatory cells; E045 Primary T cells effector/memory enriched; E046 natural killer cells; E047 T CD8+ naive cells; E048 T CD8+ memory cells; E062 mononuclear cells). Permutation testing was carried out as before, except that only one-tenth of the total number of possible permutations was used. FDR control was carried out as described above.

### Data access

As a resource for other researchers, all of our meQTL findings, including positional information, best association statistics and basic annotation data, are provided in Table S8 in Additional file 1. Methylome data have been deposited in dbGAP (http://www.ncbi.nlm.nih.gov/gap/) with the accession number phs000608.

## Additional file

Additional file 1: **Supplementary Materials.** (ZIP 58629 kb)

## Abbreviations

bp: Base pair
eQTL: Expression quantitative trait locus
FDR: False discovery rate
GWAS: Genome-wide association study
LD: Linkage disequilibrium
MAF: Minor allele frequency
Mb: Megabase
MBD: Methyl-CpG binding domain
MDS: Multi-dimensional scaling
meQTL: Methylation quantitative trait locus
NGS: Next-generation sequencing
NHGRI: National Human Genome Research Institute
OR: Odds ratio
PC: Principal component
QC: Quality control
QQ: Quantile-quantile
QTL: Quantitative trait locus
SD: Standard deviation
SNP: Single nucleotide polymorphism
TF: transcription factor
WGBS: Whole genome shotgun bisulfite sequencing
