# Supplementary material for: High density methylation QTL analysis in human blood via next-generation sequencing of the methylated genomic DNA fraction
Source: Genome Biol. 2015 Dec 23;16:291. doi: 10.1186/s13059-015-0842-7 (PMC4699364; doi:10.1186/s13059-015-0842-7)
Supplement: Supplementary file 1 — Supplementary Materials. (ZIP 58629 kb) [file 13059_2015_842_MOESM1_ESM.zip › McClay-Shabalin meQTL Sept 2015 second revision.docx]

**High density methylation QTL analysis in human blood via next-generation sequencing of the methylated genomic DNA fraction**

Joseph L. McClay^1,2*^, Andrey A. Shabalin^2*^, Mikhail G. Dozmorov^3^, Daniel E. Adkins^2^, Gaurav Kumar^2^, Srilaxmi Nerella^2^, Shaunna L. Clark^2^, Sarah E. Bergen^4,5.6^, Swedish Schizophrenia Consortium, Christina M. Hultman^4^, Patrik K.E. Magnusson^4^, Patrick F. Sullivan^4,7^, Karolina A. Aberg^2^, Edwin J.C.G. van den Oord^2^.

^*^ Equal contribution (joint first authorship)

^1^ Department of Pharmacotherapy and Outcomes Science, Virginia Commonwealth University, Richmond, Virginia, USA;

^2^ Center for Biomarker Research and Precision Medicine, Virginia Commonwealth University, Richmond, Virginia, USA;

^3^ Department of Biostatistics, Virginia Commonwealth University, Richmond, Virginia, USA;

^4^ Department of Medical Epidemiology and Biostatistics, Karolinska Institute, Stockholm, Sweden;

^5^ Psychiatric and Neurodevelopmental Genetics Unit, Massachusetts General Hospital, Boston, Massachusetts, USA;

^6^ Stanley Center for Psychiatric Research, Broad Institute of MIT and Harvard, Cambridge, Massachusetts, USA;

^7^ Department of Genetics, University of North Carolina School of Medicine, Chapel Hill, North Carolina, USA;

Correspondence to:

Dr. Joseph L. McClay, Department of Pharmacotherapy and Outcomes Science, Smith Building, Medical College of Virginia campus, Virginia Commonwealth University, 410 North 12^th^ Street, Richmond, VA 23298, USA. Email: jlmcclay@vcu.edu

Dr. Andrey A. Shabalin, Center for Biomarker Research and Precision Medicine, McGuire Hall, Medical College of Virginia campus, Virginia Commonwealth University, 1112 East Clay Street, Richmond, VA 23298, USA. Email: ashabalin@vcu.edu

**Key words**: DNA methylation, next-generation sequencing, GWAS, single nucleotide polymorphism, quantitative trait loci, chromatin states

**ABSTRACT**

**Background**: Genetic influence on DNA methylation is potentially an important mechanism affecting individual differences in humans. We use next-generation sequencing to assay blood DNA methylation at approximately 4,5 million loci, each comprising 2,9 CpGs on average, in 697 normal subjects. Methylation measures at each locus are tested for association with approximately 4,5 million SNPs to exhaustively screen for methylation quantitative trait loci (meQTLs).

**Results**: Using stringent false discovery rate control, 15% of methylation sites show genetic influence. Most meQTLs are local, where the associated SNP and methylation site are less than 1 Mb apart. Distant meQTLs and those spanning different chromosomes are less common. Most local meQTLs encompass common SNPs that alter CpG sites (CpG-SNPs). Local meQTLs encompassing CpG-SNPs are enriched in regions of inactive chromatin in blood cells. In contrast, local meQTLs lacking CpG-SNPs are enriched in regions of active chromatin and transcription factor binding sites. Of 393 local meQTLs that overlap disease-associated regions from genome-wide studies, a high percentage encompass common CpG-SNPs. These meQTLs overlap active enhancers, differentiating them from CpG-SNP meQTLs in inactive chromatin.

**Conclusions**: Genetic influence on the human blood methylome is common, involves several heterogeneous processes and is predominantly dependent on local sequence context at the meQTL site. Most meQTLs involve CpG-SNPs, while sequence-dependent effects on chromatin binding are also important in regions of active chromatin. Abundant local meQTLs resulting from methylation of CpG-SNPs in inactive chromatin suggests that many meQTLs lack functional consequence. Integrating meQTL and Roadmap Epigenomics data could assist fine mapping efforts.

**BACKGROUND**

Methylation of DNA cytosine residues is an important mechanism in the control of gene expression and the determination of cell fate in development [1–3]. DNA methylation is known to vary with sex, age and exposure to environmental factors [4] and changes to methylation patterns have been associated with many common diseases [5]. Methylation is also under genetic influence and locus-specific methylation levels are often correlated in related individuals [6, 7]. This observation has motivated the mapping of loci where DNA methylation is under genetic control, also known as methylation quantitative trait loci (meQTLs).

Several early reports focusing on candidate loci found instances of DNA methylation levels correlated with sequence variants [8]. However, the advent of genome-wide association studies (GWAS) and methods for interrogating methylation at multiple loci has enabled the mapping of meQTLs on a larger scale [7, 9, 10]. Genotype array marker densities have increased dramatically over time and, when coupled with imputation, now enable comprehensive surveys of most common SNPs in the human genome [11]. Our ability to interrogate genome-wide DNA methylation (the “methylome”) has developed in parallel with these technologies [12, 13]. Most published genome-wide meQTL studies [14–16] measured methylation via the Illumina Infinium 27K array, capable of interrogating ~27,000 methylated sites, while the most comprehensive study to date used the latest Infinium chip to analyze ~450,000 sites in lung tissue [17]. However, there are approximately 27 million autosomal CpGs in the human genome, of which a substantial portion is methylated in most tissues surveyed [18]. It is therefore apparent that only a small fraction of possible meQTLs has been surveyed to date. In addition, DNA methylation outside CpG islands, traditionally the focus of methylation research, plays a role in the regulation of transcription [19]. This suggests that more comprehensive meQTL surveys could be invaluable in understanding genetic regulatory processes.

In this context, next-generation sequencing (NGS) methods offer a significant advance over array-based methylation detection [12]. Whole genome shotgun bisulfite sequencing (WGBS) yields single base resolution methylation data for every cytosine in the genome, but it is not yet economically feasible in the large sample numbers required for genetic epidemiology [13]. This factor is particularly relevant for meQTL studies, where our ability to detect effects depends not only on the proportion of the genome covered by methylation and SNP data, but also adequate statistical power derived from large sample numbers. As an alternative to WGBS, enrichment for the methylated genomic fraction followed by NGS can yield information on many millions of methylation sites [20]. In this study, therefore, we use methyl-CpG-binding domain protein-based enrichment coupled to NGS (MBD-seq) to assay DNA methylation in human blood [21]. MBD-seq has been demonstrated to be sensitive and capable of identifying differentially methylated regions [20–25], to detect previously reported robust associations [26], and to produce findings that replicate using more sensitive, targeted technologies [27]. Although MBD-seq cannot pinpoint the specific CpG that caused an association in regions with multiple CpGs, its resolution is approximately the size of the sequenced fragment (150-250 bp). All these properties make MBD-seq a very efficient tool for high-density methylome-wide studies.

We used MBD-seq to measure methylation at over 4.5 million unique loci in DNA from peripheral blood from normal subjects. We then tested each locus-specific DNA methylation measure for association with a high density SNP genotype panel, augmented with imputed genotypes from 1000 Genomes [28], to exhaustively identify common variant meQTLs. To control for unmeasured confounders in the methylation data, such as could be caused by heterogeneous cell types in the source tissue (i.e. blood), we included the top principal components from the methylation data as covariates in the association testing. Our study provides a comprehensive up-to-date overview of genetic influence on the methylome in human blood DNA, outside of a specific disease context, and provides insights into the processes that generate meQTLs.

**RESULTS**

***Data summary***

Our study population comprised 697 subjects from Sweden (see sample description in Supplementary Table S1) who were controls from a larger genetic study on the etiology of schizophrenia [27]. Due to the specific nature of sex chromosome methylation patterns, we focus on the autosomes. SNPs were genotyped as described previously [29, 30]. Imputation was carried out with Minimac [28] using 1000 Genomes reference panels v3 using MAF>0.05 and *r^2^*>0.5 as thresholds. Our MBD-seq data consisted of 31.6 million methylation-enriched reads per subject (SD = 13.4 million) after alignment and quality control (QC). We used these reads to estimate fragment coverage at each of the ~27 million autosomal CpG sites in the human reference genome (hg19), where higher coverage indicates higher levels of methylation [31]. Our methylation data preprocessing involved exclusion of sites showing poor mappability, data reduction by combining highly correlated coverage estimates at neighboring CpGs [31–33], and discarding of unmethylated sites (< 97.5 percentile of background coverage levels). Based on preliminary results, we also excluded 5 Mb of pericentromeric or 1 Mb of subtelomeric regions because polymorphic tandem repeats in these regions [34] were likely causing spurious inflation of significant associations. The final dataset comprised 4,532,060 SNPs and 4,544,738 methylation sites. Each methylation site, on average, comprised 2.91 CpGs and spanned 71.1 bp.

***Numbers and genomic distribution of meQTL effects***

We used Matrix eQTL [35] to test all SNPs for association with methylation levels at every site. Our testing procedure accounted for covariates such as ancestry (four MDS dimensions), sex, sample batch and other laboratory assay variables. To prevent potential confounding effects from unmeasured sources of methylation variation, such as arises from cell type heterogeneity in blood, we also included the top seven principal components (PCs) from the methylation data (Supplementary Fig. S1) as covariates in the association testing. To confirm that these PCs were not associated with genetic variation, we ran GWAS on the PC scores. Quantile-quantile (QQ) plots (Supplementary Fig. S2) indicated that methylation variation captured by the PCs was not under detectable genetic control because no SNPs were significantly correlated with any PC.

Results from our primary analysis are summarized in Table 1. Following previous meQTL studies [15], tests were divided into “local” (SNP ≤ 1 Mb from methylation site) and “distant” (SNP > 1 Mb from site). Distant effects were further subdivided into same chromosome and cross chromosome findings (see Fig. 1). Associations were considered significant if they passed a stringent false discovery rate (FDR) threshold of 0.01, with the FDR calculated separately for each group of tests. Although the number of distant tests was much greater than the number of local tests (20,168 billion distant, versus 16 billion local), significant findings among local tests were 189,000-fold more common compared to all distant tests, or 5.8 million-fold more common compared to cross-chromosome tests (see QQ plots in Fig. 2A). The preponderance of local effects is further explored in Fig. 2B-D. Here we show that the proportion of significant findings increased as distance between SNPs and their associated meQTLs diminished, indicating that genetically-driven methylation is typically co-localized with the variation affecting it.

To illustrate the genomic distribution of effects, plots of meQTLs by chromosome position are provided in the Supplementary Material (pp6-27), with an interactive browser available at http://www.pharmacy.vcu.edu/biomarker/resources/supplementary. In Figure 3, we show the pattern of results around the three most significant meQTLs by *p*-value in our analysis. Figure 3 also shows SNP-SNP and methylation-methylation correlations in the same distance windows. SNP-SNP correlations were much more extensive than those between methylation sites at similar physical distances. Blocks of SNPs in close proximity and in linkage disequilibrium (LD) showed association with the same methylation site(s), as expected. However, SNPs also tend to be associated with several methylation sites within the same LD block. These trends are observed throughout the genome, from LD blocks of a few kilobases to the largest block we observed, spanning over 20 Mb on chromosome 8 (see Supplementary Material p13). These observations illustrate that LD extending for many Mb can spuriously suggest the presence of long-range effects. Furthermore, the overall number of significant tests is influenced by LD, whereby many highly correlated SNPs in close proximity tag each meQTL. This, coupled with the broad distribution of meQTLs throughout the genome and the relatively good statistical power provided by our study sample, explains our observation that 97.7% of all SNPs tested were associated with methylation at one or more loci within 1 Mb. As such, the number of unique methylation sites under genetic control, rather than the number of significant SNP-methylation site associations, is arguably a better representation of the extent of genetic influence on the methylome. In our analysis, methylation levels at 15% of sites were associated with one or more local SNPs. That is, 15% of methylation sites were local meQTLs. These were 166-fold more common than all distant meQTLs and 2389-fold more common than cross-chromosome meQTLs.

***Replication of findings***

Our methylation data were obtained from control subjects who formed part of a larger study of the psychiatric disorder schizophrenia [27]. While methylation differences exist between cases and controls, we considered that the case sample would allow us to obtain a lower bound estimate of the replication rate of our findings. The case sample comprised 711 patients of similar age and sex distribution to the controls (see Supplementary Material Table S1). We calculated *p*-values in the replication sample for methylation site-SNP pairs that passed 1% FDR control in the primary analysis. We then computed the π1 statistic (estimate of the proportion of true positives in a *p*-value distribution) [36] for these *p*-values. The π1 statistic was 95%, 98.7%, and 99.3% for local, distant same-chromosome, and cross-chromosome tests respectively, indicating very high replication rates. Figure 4 illustrates replication agreement.

***Genetically variable CpGs as a mechanism driving local meQTLs***

SNPs that create or abolish CpGs (CpG-SNPs) have been suggested as genetic drivers of individual differences in methylation [37]. We hypothesized that this mechanism produced local meQTL effects in our study. Such meQTLs would result from CpG-SNPs at the methylation site of interest, with other local SNPs in LD with the CpG-SNP behaving as proxies of this phenomenon (Fig. 1D). We identified all CpG-SNPs in dbSNP 135 (30.1% of SNPs) and then quantified how many methylation sites in our analysis encompassed a CpG-SNP, including a flanking region ± 250 bp of the site boundary. This 250 bp window size represents the approximate maximum length of a sequenced fragment in our study, and therefore is the maximum distance from a locus at which a CpG-SNP could directly affect our methylation measure. In part II of Table 1, we show that 75% of methylation sites under local genetic influence contain a CpG-SNP with MAF>0.05, compared to 33% of all sites genome-wide. This enrichment is also observed, albeit to a lesser extent, at lower CpG-SNP MAF thresholds (Supplementary Table S2). Thus most, but not all, local meQTLs could be explained by CpG-SNPs. By comparison, cross-chromosome meQTLs showed no enrichment for CpG-SNPs.

***Bioinformatics analysis of meQTL findings***

We tested if our meQTLs (i.e. those methylation sites with significant SNP associations) were enriched in several categories of genomic annotations. We performed our bioinformatics analyses in two phases. In the first phase, we looked at aggregate annotation categories (e.g. genes, transcription factor binding sites), with *p*-values generated via up to 4 million permutations. We initially focused on local findings, first examining enrichment among all local meQTLs and then split our data to characterize enrichment patterns for meQTLs with CpG-SNPs and those without. In the second bioinformatics analysis phase, we looked at specific annotations in more detail, reducing the number of permutations to one tenth of those carried out in the first phase and including false discovery rate control due to the larger number of tests. Initial phase 1 analysis found some enrichment of genomic duplications, copy number variants, and pseudogenes among meQTL findings. This was modest for local effects but substantially greater for cross-chromosome as compared to local meQTLs (see Supplementary Table S3). We eliminated methylation sites overlapping these features and re-analyzed the remainder.

*Initial genomic annotation analysis of all local meQTLs*

The results for the phase 1 analysis of all local meQTLs are summarized in Figure 5 (details are provided in Supplementary Table S3). To estimate enrichments that could be observed by chance, we used the annotations for all 4.5 million methylation sites that we assayed as our background set in the permutation analysis. We found that local meQTLs were significantly less likely to overlap with almost all functional features tested, including CpG islands, genes, promoter regions, DNaseI hypersensitive regions, etc, compared to sites that were not meQTLs. However, local meQTLs were significantly more likely to overlap with GWAS hits from the National Human Genome Research Institute (NHGRI) GWAS catalog (odds ratio=3.09, *p*=2.6x10^-75^). It should be noted that in this instance we consider a very specific overlap, i.e. the GWAS catalog SNP is within the boundary of the methylation site. This finding was also observed in our replication sample (OR=3.4, *p*=4.8x10^-79^). Of the 393 methylation sites under local genetic control overlapping the GWAS catalog in our main analysis (Supplementary Table S4), 366 encompassed a CpG-SNP with MAF>0.05 while 387 encompassed a CpG-SNP with MAF>0.01. In our phase 2 analysis, we examined the specific phenotypes contributing to the NHGRI GWAS catalog overlap with all local meQTLs. We show results for the 21 phenotypes passing FDR<0.01 in Table 2. A common theme was not apparent, with genetic effects on methylation appearing to influence traits such as body morphology and cardiovascular, autoimmune and psychiatric disorders, amongst others.

*Genomic annotation analysis of local meQTLs with and without CpG-SNPs*

Common (MAF>0.05) CpG-SNPs were present at most (75%), but not all, local meQTLs. This implies that different mechanisms are operating to influence methylation at meQTLs without CpG-SNPs. To compare patterns of genomic features for local meQTLs with CpG-SNPs and those without, we reran our phase 1 analysis stratifying by common CpG-SNP (MAF>0.05) presence or absence. Thus, in this analysis, local meQTLs with CpG-SNPs were compared to a background set of all methylation sites with CpG-SNPs (out of the 4.5 million assayed in our study). Similarly, local meQTLs without CpG-SNPs were compared to all methylation sites lacking CpG-SNPs.

We found that local meQTLs with common CpG-SNPs showed similar patterns to our observations for all local meQTLs, except that they were much less enriched for G-quadruplexes (see Supplementary Table S3). For meQTLs lacking common CpG-SNPs, these were significantly more likely to overlap with transcription factor (TF) binding sites (OR=1.11, *p*=7.24 × 10^-18^) and DNase clusters (OR=1.12, *p*=1.11×10^-37^) than those without. Notably, these findings became more pronounced when we excluded sites with any CpG-SNP, regardless of MAF (TF binding sites OR=1.25, *p*=3.56×10^-39^; DNase clusters OR=1.24, *p*=1.59×10^-59^). These findings were also observed in the replication sample, with similar ORs and significance levels (Supplementary Table S3). To identify the specific TFs accounting for the observed aggregate enrichment at local meQTLs, we obtained the individual genome-wide binding profiles for more than 200 TFs from ENCODE [38]. We then analyzed each TF binding profile separately, rather than in aggregate as above, and results are shown in Supplementary Table S5. The individual TFs that displayed the greatest enrichment and passed FDR<0.01 were ZBTB33 (OR = 3.85, *p* = 9.67 × 10^-18^), p300 (OR = 3.06, *p* = 9.67 × 10^-18^) and TR4 (OR = 2.84, *p* = 9.67 × 10^-18^). As shown in Supplementary Table S5, binding sites for 66 unique TFs showed enrichment for local meQTLs. This large number may be partly explained by the tendency of TF binding profiles to overlap in regions critical for transcriptional regulation [38]. Nevertheless, our finding suggests that genetic influence on TF binding can have substantial influence on local methylation levels and that this mechanism is potentially applicable to a large number TFs.

*Enrichment analysis of local meQTLs in Roadmap Epigenomics chromatin states*

In our phase 1 analysis, the presence or absence of CpG-SNPs differentiated local meQTLs with respect to enrichment in TF binding sites and DNase clusters. This suggested that meQTLs without CpG-SNPs were more likely to occur in regions of active chromatin. To study this in more detail, we looked for local meQTL enrichment in genomic regions classified into specific chromatin states. Local meQTLs were divided according to presence or absence of CpG-SNPs (any MAF). Chromatin state classification was according to an 18 state model from the Roadmap Epigenomics Consortium [18] for peripheral blood cells, of which there was information on 14 distinct cell types. Results are summarized in Figure 6 and full details are provided in Supplementary Table S6). A very distinct pattern of results was obtained for local meQTLs with CpG-SNPs, which were strongly enriched in heterochromatin and quiescent regions, in addition to regions harboring zinc finger protein genes and repeats, while being significantly depleted in other chromatin states. In contrast, meQTLs without CpG-SNPs were enriched around transcription start sites and enhancers. These were also enriched in some inactive chromatin regions, notably in repressed polycomb regions. There was strong agreement in the pattern of enrichment across cell types, indicating a broad consistency in chromatin activity patterns among the major classes of peripheral blood cells studied.

*Secondary analysis of local meQTLs overlapping the NHGRI GWAS catalog*

Of the 393 local meQTLs that overlapped the NHGRI GWAS catalog, the vast majority (>93%) encompassed a common CpG-SNP (MAF>0.05), while practically all (>98%) encompassed a CpG-SNP with MAF>0.01. However, our chromatin state analysis showed that meQTLs with CpG-SNPs were much more likely to occur in quiescent or heterochromatin regions. Given that most local meQTLs with CpG-SNPs are in regions unlikely to affect phenotype, we speculated that those overlapping the NHGRI GWAS catalog were in some way distinct. We therefore compared the local meQTLs with CpG-SNPs overlapping the NHGRI GWAS catalog to all local meQTLs with CpG-SNPs. Due to the smaller numbers of meQTLs being tested, this analysis had lower power relative to those above and none of the findings passed our stringent threshold of FDR<0.01. However, five tests did pass a more typical FDR<0.05 threshold (Supplementary Table S7). Specifically, those local meQTLs with CpG-SNPs overlapping the GWAS catalog were less likely to occur in quiescent and heterochromain regions and more likely to occur in regions of active chromatin, specifically active enhancers (max OR=2.2, *p*= 1.9x10^-4^) than all local meQTLs with CpG-SNPs. These results suggest that meQTLs with CpG-SNPs in regions of active chromatin associated with disease could be high priority targets for further characterization in disease studies.

*Genomic annotation analysis of SNPs and cross-chromosome meQTLs*

For distant meQTLs, to prevent possible contamination from local effects in regions where LD exceeds 1 Mb, we focused on cross-chromosome findings. Cross-chromosome meQTLs (i.e. methylation sites associated with SNPs on other chromosomes) were significantly more likely to overlap with exons (OR = 5.24, *p* = 3.39 × 10^-18^). This association was also apparent in the replication sample (OR = 4.91, *p* = 7.78 × 10^-19^), suggesting that some cross-chromosome findings may have functional relevance (see Supplementary Table 3). However, unlike local meQTLs, there was no enrichment for cross-chromosome meQTLs in the NHGRI GWAS catalog, suggesting any functional effects they may have are benign.

Phase 1 annotation analysis for local and distant SNP effects are also included in Supplementary Table 3 for completeness. In these SNP analyses, only common SNPs (MAF > 0.05) were included as the background set because only common SNPs were included in our study. The local SNP findings showed no enrichment for most features tested. However, the vast majority of SNPs were associated with one or more local meQTLs so the discriminatory power of this local analysis is limited. Finally, cross-chromosome SNP findings were not significantly enriched for any phase 1 annotation category.

**DISCUSSION**

All previous studies of meQTLs found abundant local effects in all tissues analyzed [9, 15, 17]. Our findings suggest that 1) any site under local genetic influence was typically associated with a SNP in very close proximity, 2) local effects tracked LD, and 3) the majority of sites under local genetic control included a CpG-SNP within their boundary. Distant effects showed no such enrichment. Taken together, these observations suggest that most meQTLs involve processes that are entirely dependent on local sequence context, or are LD proxies of such processes, consistent with previous studies of allele-specific methylation [39, 40]. Previous studies have noted the importance of CpG-SNPs as mediating genome-epigenome interaction [37, 40] and our study provides extensive quantitative evidence to support this.

Several different processes appear to be working to cause meQTLs. For those meQTLs overlapping CpG-SNPs, absence of the CpG sequence in some individuals will clearly prevent methylation. However, in others the presence of a CpG means only that it *can* be methylated, not necessarily that it *will* be. Methylation of the CpG in the cell type of interest is necessary for the meQTL effect to be observed. In our data, we observed enrichment of meQTLs with CpG-SNPs in quiescent and facultative heterochromatin regions, both of which are heavily methylated (>75%) [18]. This suggests that, for this class of meQTLs, we are simply picking up the sequence differences at polymorphic CpGs between individuals in heavily methylated regions. Quiescent and heterochromatin regions are also transcriptionally inactive. A small number of previous studies have examined meQTLs and expression QTLs (eQTLs) in the same tissue and observed very low concordance. For example, Gibbs *et al*. [15] found that only 4.8% of significant meQTLs were also an eQTL. The fact that most of our meQTLs included CpG-SNPs and these were in transcriptionally inactive chromatin regions could be one factor in explaining the low concordance between meQTLs and eQTLs.

One expectation based on these observations could be that meQTLs with CpG-SNPs typically have no effect on phenotype. However, this is somewhat incongruent with our finding that local meQTLs were enriched in loci from the NHGRI GWAS catalog and almost all sites overlapping the catalog encompassed CpG-SNPs. For example, the most significant site among the overlap spanned 157bp at the *TCF7L2* locus on chromosome 10 (114,753,967-114,754,123) and the associated SNP was rs34872471 (*p*=1.4×10^-126^). The CpG-SNP within the boundary of this site, rs7901695 (chr10:114,754,088), was associated at a similar level of significance (*p*=2.1×10^-114^). It is considered to be a confirmed susceptibility variant in diabetes [41, 42], with over 30 publications at time of writing linking it to the disease. Mutation of CpGs at certain critical loci is considered to be an important etiological mechanism for complex diseases [37]. We observed almost 400 findings that overlapped with the GWAS catalog, including associations with many different disease genes and disorders, suggesting that meQTLs with CpG-SNPs overlapping the GWAS catalog were distinct in some way. Our analysis showed that these meQTLs were more likely to be in active chromatin, with our most significant enrichment in active enhancers. This echoes previous studies showing enrichment of disease or trait-associated variants in specific chromatin states [43] or accessible regions [44]. Recent findings from the Roadmap Epigenomics Consortium also indicated enrichment of disease-associated variants in active enhancers, most notably those associated with H3K4me1 and H3K27ac histone marks [18]. Based on these findings, we suggest that local meQTLs with CpG-SNPs in active chromatin regions in the relevant tissue should be priority targets for functional follow-up in disease mapping studies. Our meQTLs from NGS are typically small, spanning only 2.9 CpGs and approximately 70 bp on average. Thus, they could enable fine-scale prioritization of specific variants from the large regions implicated in GWAS.

Where local meQTLs lack CpG-SNPs, alternative mechanistic explanations must be considered. It is known that non-CpG variants influence the binding of *cis*-acting factors that, in turn, affect methylation levels. For example, binding of Sp1 serves to prevent methylation at some CpGs in promoter regions [45]. We observed that sites lacking CpG-SNPs, but with local meQTLs, were more likely to overlap TF binding sites, in agreement with this mechanistic explanation. However, the extent of this phenomenon is greater than perhaps previously understood, with a broad range of TF binding profiles showing significant enrichment for meQTLs without CpG-SNPs. We observed that meQTLs without CpG-SNPs were enriched in active regions of chromatin, particularly at the transcription start site and flanking regions. This is congruent with binding of TFs being the underlying mechanism. However, we also observed meQTLs without CpG-SNPs to be enriched in some inactive regions, particularly repressed polycomb (RepPC) regions. Therefore, our results suggest that genetic differences that affect binding of several different classes of chromatin binding factors are an important influence on the methylome.

**CONCLUSIONS**

Our use of high-density genome-wide SNP genotyping and imputation based on 1000 Genomes data enabled us to capture much of the common SNP variants in this sample. Our use of NGS to assay DNA methylation enabled us to assess most methylated CpGs in the non-repetitive portion of the genome. We confirmed that genetic influence on methylation is a pervasive phenomenon throughout the genome and, for the most part, highly localized in its effect. Our findings suggest that several mechanisms can generate meQTLs. These include CpG-SNPs and variants that interfere with chromatin binding for several classes of proteins. The very large number of local meQTL effects attributable to CpG-SNPs, coupled with the fact that they typically occur in non-functional regions of the genome, suggests that most have little phenotypic consequence. However, the observed enrichment of meQTLs in disease or trait-associated regions from the NHGRI GWAS catalog indicated that a small portion of CpG-SNPs have arisen in regions of the genome where they may exert significant influence on phenotype. Integration of meQTLs with other data, such as RoadMap Epigenomics, could aid in functional interpretation of SNPs identified in disease GWAS. We therefore provide all our meQTL findings, including positional information and association statistics, in Supplementary Table S8.

### METHODS

### *Ethics.* All procedures were approved by the institutional review board at the Karolinska Institutet, Stockholm, Sweden (IRB/KI 04/-499/4) and further locally approved by the Virginia Commonwealth University institutional review board (IRB#. HM12499). Subjects provided written informed consent (or legal guardian consent and subject assent). All experimental methods comply with the Helsinki Declaration.

### *Subjects and biological sampling**.* Subjects were controls collected as part of a larger project entitled “A Large-Scale Schizophrenia Association Study in Sweden”. This overarching project [30, 46, 47] aims at understanding the etiology of schizophrenia and bipolar disorder plus their clinical and epidemiological correlates. Peripheral blood was donated at the local medical facilities of the participants. DNA was extracted from EDTA blood using the Gentra Puregene kit for automated extraction with the Autopure LS robot (Qiagen).

*Genome-wide SNP genotyping, quality control, and imputation.* Genotyping was carried out as described previously [29, 30]. Briefly, subjects were genotyped with Affymetrix genome-wide SNP Arrays 5.0 or 6.0, or Illumina OmniExpress. All genotyping was conducted at the Broad Institute of Harvard and the Massachusetts Institute of Technology. Genotypes were called using the Birdsuite (Affymetrix) or BeadStudio (IIllumina). Quality control (QC) exclusionary measures for subjects were: genotype call rates <95%; ancestry outliers via multidimensional scaling; a randomly selected member of any pair of subjects with high relatedness (*π*>0.20); and suspected sample error or contamination indicated by high heterozygosity or indeterminate genetic sex. SNPs were excluded for marked departure from Hardy–Weinberg equilibrium (*p*<1×10^−6^), low minor allele frequencies (<1%), and non-random genotyping failure, inferred from the flanking haplotype background using the PLINK ‘mishap’ test (*p*<1×10^−10^). Plate-based associations of *p*<1×10^−6^ were taken as evidence of non-random genotyping failure, based on a comparison of allele frequency of each plate to all others and were removed on a plate-by-plate basis. To enhance coverage, we imputed SNPs from 1,000 Genomes data (phase I version 3) using Minimac, after phasing genotypes with MACH 1.0 [28]. After selecting on MAF>0.05 and imputation quality measure *r*^2^>0.5, a total of 4,761,800 imputed and genotyped SNPs were available for meQTL association testing.

*MBD-seq.* Our methods and analysis pipeline make use of methyl-CpG-binding domain (MBD) protein-based enrichment and sequencing (MBD-seq) as described previously [26, 27, 31]. Briefly, genomic DNA was sheared to median fragment size=125 bp (Covaris E210) and the methylated portion captured using MethylMiner (Invitrogen), followed by elution in 500mM NaCl. Methylated DNA fragments were sequenced (SOLiD, Life Technologies) using standard multiplexed single end (50 bp) methods. The SOLiD system aligns in color space and uses 2-base encoding [48], producing two ‘color calls’ for each base. After deleting poor quality reads (>2 missing color calls), we obtained an average of 67.3 million (SD = 26.9 million) total reads per sample. This exceeds the recommended 30-60 million reads required for genome-wide methylation analysis via enrichment-based methods [49, 50]. The mean quality value (QV = -10log_10_(*p*) with *p* being the probability of an error) per color call was 21.4 (SD = 1.1). Reads were aligned to the human reference genome (hg19/GRCh37) using BioScope 1.2 (Life Technologies). The percentage of mapped reads was 69.2% (SD=6.2). We deleted reads with multiple poor quality alignments and high copy number duplicate reads were collapsed to single reads. This led to the elimination of 32.1% of the mapped reads. After all QC, we obtained on average 31.6 million reads per subject (SD=13.4 million).

MBD-seq methylation measures. Locus-specific methylation measures were obtained by summing the number of fragments expected to cover each CpG. Note that methylation of any CpG in a DNA fragment, not just the sequenced 50bp, could lead to its capture by MBD protein. Hence we define locus-specific methylation measures as the expected number of DNA fragments covering each CpG [33]. Specifically, fragments whose sequenced part is covering a CpG contribute a unit to its methylation measure. For some fragments, it is not known whether they cover a given CpG, so their contribution to the methylation measure is set to the estimated probability that the fragment covers the CpG. This probability is a function of fragment size distribution. We estimated the fragment size distribution empirically from the distribution of reads around isolated CpGs [33]. The calculation of methylation measures can be schematically expressed as

$${Methylation\_Measure}_{CpG}=\sum_{fragments near CpG} \hat{P}\left( Fragment covers the CpG \right).$$

The average number of fragments covering a particular CpG depends not only on the methylation status of that site but also on the number of methylated CpGs in the region [20]. To make coverage estimates more comparable across sites and improve the correlation with actual methylation levels, coverage estimates can be further normalized using the local CpG density as a proxy for the number of methylated CpGs in the region [50, 51]. However, our meQTL analyses essentially involve the calculation of correlations between SNPs and quantile normalized methylation levels. As these correlations are not affected by monotone transformations of methylation levels, for sake of simplicity we did not use such a normalization step.

Thirty-six percent (10.5 million) of all ~27 million autosomal CpGs in the reference genome (hg19/GRCh37) were eliminated because of predicted alignment problems, as observed in an *in silico* analysis [31]. The majority (71.8%) of these were in regions flagged as repetitive elements by RepeatMasker. To reduce the size of the data set, the remaining ~16 million CpGs were adaptively combined by collapsing highly inter-correlated coverage estimates at adjacent CpG sites into a single mean coverage estimate [31, 52]. Prior to association testing, we dropped sites with very low levels of coverage as these were likely unmethylated (<97.5% of background coverage at non-CpG sites, where the latter are defined as loci with no CpGs within 400 bp).

We previously reported in-depth quality metrics for this methylation dataset [31]. Briefly, the ratio between the median coverage at a CpG, i.e. the methylation signal, versus coverage at a non-CpG, i.e., the background noise, is >40. This indicates the signal to noise ratio is high. Second, in an analysis of 73 technical replicates, we observed a median correlation of 0.92 for genome-wide methylation measures between replicates. This indicates that our MDB-seq measures are robust and reproducible. Several studies have compared MBD-seq quality and genome-wide coverage to other methods [20, 22, 25]. Enrichment-based sequencing methods, such as MBD-seq and MeDIP, are cheaper than WGBS and provide better genome-wide coverage than microarrays [13]. By measuring the relative enrichment of methylated DNA rather than absolute levels, enrichment-based methods are somewhat less accurate than Infinium arrays or bisulfite sequencing for quantifying DNA methylation levels in partially methylated regions. Enrichment-based methods can, however, distinguish between methylated and unmethylated regions almost as precisely as bisulfite sequencing [49]. Compared to MeDIP, MBD-seq is less noisy (picks up fewer sporadically methylated sequences) but only assays methylation at CpGs [21]. Furthermore, standard MBD-seq preferentially assays CpG-dense regions [25]. To improve methylome-wide coverage, we used an existing protocol variant that increases the relative number of fragments from CpG-poor regions by eluting the captured methylated fraction with 0.5 M NaCl [31].

Association analyses and FDR control. To test for association between genotype and methylation measurements (each SNP vs. each methylation site) we used Matrix eQTL [35], a computationally efficient analysis tool implemented in R (http://www.r-project.org/). Methylation values were first corrected using the inverse quantile normal transformation of the ranked values. This robust approach greatly reduces the effect of outliers, while retaining more power than rank-based procedures [53]. To eliminate possible technical artifacts, lab variables and sample batch were included as covariates in the linear regression model. We also performed a principal components analysis (PCA) of the methylation data to eliminate unmeasured confounders [32]. As is true for most tissues, blood consists of a variety of cell types. By using whole blood we study an “average” methylation pattern. This can produce false positives if two conditions hold simultaneously 1) the relative abundance of common cell types is correlated with the outcome variable of interest, and 2) methylation patterns of these cell types differ. Ideally, we would have methylation data obtained from separated blood cells [54] to identify sites that are at risk for being false positives. However, PCA provides an alternative solution [54–56]. Subjects with similar cell type compositions will have more similar multi-locus methylation patterns and these patterns will be captured and regressed out through the PCs. Based on a scree plot (see Supplementary Fig. S1), the first seven PCs were selected for inclusion as covariates in the MWAS.

For the SNP data, we used 4 MDS dimensions to control for ancestry, as used in the original report of GWAS in this sample [29]. Due to the large size of the data sets, methylation data and SNP genotypes were split by chromosome and Matrix eQTL was applied separately for each pair of chromosomes. We controlled the false discovery rate (FDR) [57] at 1%. Separate FDR calculations for local, distant same chromosome and cross-chromosome tests were performed to account for variations in the proportion of null tests across these scenarios and ensure the FDR was efficiently controlled at the desired 1% level in all cases. Namely, different *p*-value thresholds were used for these three groups of test to ensure at most 1% of discoveries were false in each group. Within each group FDR was calculated using standard Benjamini–Hochberg procedure, which is known to be more conservative than other common FDR procedures (as it assumes that the fraction of non-null tests is small or zero).

The Benjamini–Hochberg FDR control procedure works as follows. First, the p-values in each group were ordered in increasing order: $P_{\left( 1 \right)},P_{(2)},\ldots,P_{(m)}$. Next, the maximum $k$ is selected such that $P_{(k)}\leq\frac{k}{m}\alpha$. The tests with smallest $k$ p-values are then declared to have FDR below $\alpha$. In our case $\alpha=0.01$ (i.e. 1%).

Bioinformatics analyses. Annotation tracks for the first analysis phase were obtained via UCSC genome browser download for human genome build hg19 and dbSNP version 135. We selected the following tracks for testing: 1) RefSeq genes and used gene positional information to calculate 2) exon, 3) intron, 4) promoter region 2 kb upstream and 5) promoter region 8 kb from transcription start site annotations. We also selected 6) conservation based on similarity between 29 eutherian mammals, 7) CpG islands (defined as GC content of ≥50% or greater, length 200 bp, CpG ratio 0.6), 8) CpG shores (2kb regions flanking a CpG island [19]), 9) repetitive elements from RepeatMasker (www.repeatmasker.org), 10) conserved transcription factor binding sequences between humans and rodents as provided in TransFac version 7.0, 11) clustered ENCODE transcription factor binding sites mapped via chromatin immunoprecipitation sequencing (ChIP-seq) [58, 59], 12) DNase clusters from the University of Washington DNaseI hypersensitivity submission to ENCODE, 13) long non-coding RNAs from Gencode version 18, 14) VISTA enhancers [60], 15) microRNA genes, 16) G-quadruplexes [61], 17) known imprinted genes (http://www.geneimprint.com), and 18) NHGRI GWAS catalog hits (www.genome.gov/gwastudies) [62].

For each methylation site or SNP, we determined overlap with each annotation category and then compared these with the set of sites/SNPs with detected meQTLs. The significance of the enrichment of sites/SNPs in each category with sites/SNPs with meQTLs was initially assessed using Fisher’s exact test (R function “fisher.test”). However, this test requires independence of observations and the neighboring sites/SNPs are likely to be correlated and thus violate this assumption. For proper assessment of statistical significance we performed permutation analysis based on circular shifts as they preserve local dependence of sites/SNPs. A circular shift permutation analysis was conducted in the following way. First, the methylation sites are ordered by genomic location. Then, the annotation tracks for the sites are shifted by B positions forward, with annotations for the last B sites assigned to the first B sites. Then, overlap of meQTLs with annotation tracks for the shifted annotation is calculated for all values of the offset B, except those shifting the original annotation by less than 1% of the total number of sites in either direction. The overlap of meQTLs with annotation tracks under no permutation is then compared to those under circular shift permutations.

We observed that the permutation distribution of the overlap counts was very close to Gaussian in each of our tests (data not shown). For each test we fitted the normal distribution to the set of overlap counts and calculated the z-score for the overlap count observed for the original non-permuted data. The permutation *p*-values were then calculated from the corresponding z-scores.

In the phase 2 bioinformatics analyses, we used a database of genomic annotations assembled in the GenomeRunner project [63] to examine enrichment of meQTLs in selected annotation classes. These included 1) individual disease-associated SNP sets from the manually curated National Human GenomeResearch Institute (NHGRI) Catalog of Published Genome-Wide Association Studies [62] (accessed on 03/16/2015), 2) cell type-specific binding sites of individual transcription factors from the ENCODE [64] (accessed on 12/01/2014) and 3) cell type-specific chromatin states according to the 18-state model from the Roadmap Epigenomics Consortium [18] (accessed on 03/18/2015). We further tested the enrichment of 393 meQTLs overlapping disease/trait-associated SNPs cataloged by NHGRI in these cell type-specific 18 chromatin states. We used only data for primary cell lines from peripheral blood (E029 monocytes; E032 B cells; E034 T cells; E037 T helper memory cells; E038 T helper naive cells; E039 T helper naive cells; E040 T helper memory cells; E043 T helper cells; E044 T regulatory cells; E045 Primary T cells effector/memory enriched; E046 natural killer cells; E047 T CD8+ naive cells; E048 T CD8+ memory cells; E062 mononuclear cells). Permutation testing was carried out as before, except that only one tenth of the total number of possible permutations was used. False discovery rate control was carried out as described above.

**DATA ACCESS**

As a resource for other researchers, all of our meQTL findings, including positional information, best association statistics and basic annotation data, are provided in Supplementary Table S8. Methylome data has been deposited in dbGAP (<http://www.ncbi.nlm.nih.gov/gap/>) with the accession number phs000608.

**ADDITIONAL FILE DESCRIPTIONS**

The following seven additional data files are available with the online version of this paper. Additional data file 1 is the main Supplementary Material document. This includes 1) Table S1 showing descriptive statistics of the sample, 2) Figure S1 showing a scree plot of the methylation data principal components, 3) Figure S2 showing the null results of genome-wide association analysis of the principal component loadings, 4) Table S2 showing proportion of methylation sites encompassing SNPs, and 5) chromosome plots of meQTL distributions.

Additional data file 2 is a Microsoft Excel file (Table S3) showing the enrichment permutation test results for our phase 1 bioinformatics analysis, split out by different categories of findings (local meQTLs, cross-chromosome meQTLs, local SNPs and cross-chromosome SNPs). Additional data file 3 is a Table (S4) describing meQTLs that overlap with the National Human Genome Research Institute (NHGRI) genome-wide association study (GWAS) catalog. Additional data file 4 is a Table (S5) showing the specific transcription factor binding profiles enriched with meQTLs lacking CpG-SNPs. Additional data file 5 is a Microsoft Excel file (Table S6) showing enrichment by chromatin state for meQTLs with and without CpG-SNPs. Additional data file 6 is a Table (S7) that lists enrichment by chromatin state for local meQTLs with CpG-SNPs overlapping the NHGRI GWAS catalog. Additional data file 7 is a Microsoft Excel file (Table S8) showing summary and positional information of all meQTLs detected in our study, split into separate categories (local, distant same chromosome and cross-chromosome meQTLs).

**COMPETING INTERESTS**

The authors declare no conflict of interest.

**AUTHORS' CONTRIBUTIONS**

JM, AS, KA and EO conceived of and designed the study. AS ran the primary analysis. JM, AS, MD and GK conducted the bioinformatics analyses. JM and AS drafted the manuscript. CH and PM collected the DNA sample. KA and EO provided the methylation data. SB, PS, CM and PM provided the GWAS data. SN participated in the sequence alignment. DA and SC participated in the design of the study. All authors read and approved the manuscript. JM and AS contributed equally to this article.

**ACKNOWLEDGEMENTS:**

This study was supported by the National Institute of Mental Health (grant R01 MH097283). The present study is part of a larger project entitled '*A Large-Scale Schizophrenia Association Study in Sweden’* that is supported by grants from NIMH (R01 MH077139) and the Stanley Medical Research Institute. Institutions involved in this project are: University of North Carolina at Chapel Hill, Karolinska Institute, Icahn School of Medicine at Mount Sinai, Virginia Commonwealth University and Broad Institute of MIT and Harvard. Dr. Adkins was supported by grant K01 MH093731. Dr. Clark was supported by grant K01 AA021266. Library construction and next generation sequencing was performed by EdgeBio Gaithersburg, MD. Thanks to Lyon van den Oord for assistance with artwork.

### FIGURE LEGENDS

**Figure 1**. *Schematic of possible meQTL effects*. Where the SNP and methylation site are within 1 megabase (Mb) of each other, this is a "local" effect (panel A). All other effects are therefore "distant', which is further sub-classified into same chromosome (panel B) and cross-chromosome (panel C) distant effects. As there are many millions of methylated sites in the genome, local SNPs with respect to one methylation site would be distant SNPs with respect to the vast majority of others. Panel D depicts the situation where a CpG-SNP affects methylation at a locus and is thus a "causative" meQTL. Other SNPs in linkage disequilibrium (LD) with the CpG-SNP will also be associated with the CpG-SNP effect on methylation, and thus will appear as meQTLs.

**Figure 2**. *Quantile-quantile* *(QQ) and Distance plots*. QQ plots are shown in the left panel (A) for three different analysis categories. The red line indicates local meQTL effects (SNP and methylation site < 1 Mb apart), the light blue line indicates distant meQTL effects where SNP and site are on the same chromosome (SNP and methylation site >1 Mb apart), while dark blue indicates cross-chromosome meQTL effects. The thin gray diagonal line indicates the expectation under the null hypothesis. The three plots on the right (B) show distance plots for meQTL effects, where the x-axis shows the distance between SNP and methylation site, while the y-axis shows the negative logarithm (base 10) of the *p*-values. Note the y-axis scale of the plots is identical to the QQ plots, allowing direct comparison. In the leftmost distance plot, the distribution of all meQTLs around methylation sites indicates that most effects occurred within a distance of 500 Kb. The central distance plot shows just the most significant SNP per methylation site, indicating that this is typically close. The rightmost distance plot displays only the closest meQTL (i.e. closest SNP with FDR<0.01) to each site. The narrow spike indicates that the phenomenon is highly localized. The difference between the left and right distance plots is primarily due to linkage disequilibrium (LD) between SNPs.

**Figure 3**. *Methylation and genotype correlations by distance*. We took the top three meQTL effects by *p*-value in the study overall and plotted the distribution of methylation-methylation (Meth-Meth) correlation, meQTL effects (SNP-Meth) and SNP-SNP correlation around these findings. Upper three panels show several Mb around the top methylation site overall, chr22:39,034,867–39,040,773 (*p*=8.08×10^-177^); middle panels show the region around site chr14:32,953,580–32,959,242 (*p*=1.10×10^-167^), while lower panels show the region around site chr3:192,884,173 – 192,886,747 (*p*=5.71×10^-165^). Correlated blocks of SNPs in close proximity show association with the same methylation site(s). This leads to the horizontal "stripes" of significant meQTL associations. However, SNPs also tend to be associated with methylation levels at several sites on the same haplotype, leading to the two-dimensional patchwork of "striped squares" along the diagonal (see detail in SNP-Meth panels). This trend was observed universally in the genome, from small LD blocks measuring a few kilobases to very large regions, such as exists around the MHC on chromosome 6 (see Supplementary Material p11). This serves to illustrate that while meQTLs tend to be co-localized with their associated methylation sites, significant LD extending for many Mb can generate apparently long-range effects.

**Figure 4**. *Replication plot* showing agreement in test statistics for our primary sample of normal individuals (controls) versus a comparable sample, in terms of age, sex and number of subjects (N = 711 - see Supplementary Table S1), of patients with a diagnosis of the mental disorder schizophrenia (cases). The distribution of effects along the diagonal indicates broad agreement for all three classes of effect.

**Figure 5**. *Enrichment of all local meQTLs in broad genomic annotation categories*. The x-axis is the odds ratio for enrichment and 99% confidence intervals are provided for all data points. We show enrichment in both our primary sample (main analysis) and in the replication sample. In all instances, significance was calculated based on >4 million permutations. Annotation categories are as follows: 1) GWAS catalog represents hits from the NHGRI GWAS catalog, 2) CpG islands, 3) exons, 4) CpG shores, defined as 2 kb flanking a CpG island, 5) G-quadruplexes, 6) narrow promoter region upstream 2 kb upstream from transcription start, 7) conserved across 29 eutherian mammals, 8) broad promoter region 8 kb upstream from transcription start, 9) conserved transcription factor recognition sequences, 10) RefSeq genes, 11) ENCODE transcription factor binding data from chromatin immunoprecipitation sequencing (ChIP-seq) experiments, 12) DNaseI hypersensitive regions, 13) introns, 14) repetitive elements, 15) long non-coding RNAs, 16) known imprinted genes, 17) VISTA enhancers and 18) microRNA genes.

**Figure 6**. *Enrichment/depletion of local meQTLs in different chromatin states*. Chromatin state classification used an 18 state model from the Roadmap Epigenomics Consortium [18] for peripheral blood cells. Testing was conducted via permutation and details (*p*-values, test statistics, etc.) are provided in Supplementary Table S6. Panels A and B show enrichment for local meQTLs with and without CpG-SNPs respectively. Effect direction is color coded, with significant (*q*-value<0.01) enrichment (OR>1) shown in red and depletion (OR<1) shown in blue. Roadmap chromatin states are broadly categorized as active and inactive [18]. Active chromatin states are as follows: Active transcription start site (TssA), Flanking transcription start site (TssFlnk), Upstream flanking transcription start site (TssFlnkU), Downstream flanking transcription start site (TssFlnkD), Strong transcription (Tx), Weak transcription (TxWk), Genic enhancer (EnhG1 and EnhG2), Active enhancer (EnhA1 and EnhA2), Weak enhancer (EnhWk), Zinc finger genes and repeats (ZNF_Rpts). Inactive chromatin classes are as follows: Heterochromatin (Het), Bivalent/poised transcription start site (TssBiv), Bivalent enhancer (EnhBiv), Repressed polycomb (RepPC), Weak repressed polycomb (RepPCWk), and Quiescent (Quies). See http://egg2.wustl.edu/roadmap/web_portal/chr_state_learning.html#exp_18state.

### TABLES

Table 1. Summary statistics for number of findings by analysis category

| **Part I - Overall findings** | Local (≤1Mb) | Distant same chromosome (>1Mb) | Cross chromosome | Total |
| --- | --- | --- | --- | --- |
| # tests (billions) | 16.7 | 1,140.2 | 19,440.1 | 20,597.0 |
| Fraction of tests significant at FDR=1% | 4x10^-3^ | 3.7x10^-7^ | 7x10^-10^ | - |
| P-value threshold for FDR = 1% | 4.05x10^-5^ | 3.74x10^-9^ | 7.03x10^-12^ | - |
| Number of tests significant at FDR=1% | 67,752,610 | 426,958 | 13,672 | - |
|  |  |  |  |  |
| Number of unique SNPs with meQTLs | 4,426,992 (97.68%) | 36,916  (0.81%) | 11,204  (0.25%) | 4,532,060 |
| Unique methylation sites with meQTLs | 683,152 (15.03%) | 3,819  (0.08%) | 286  (0.01%) | 4,544,738 |
|  |  |  |  |  |
| **Part II - By methylation site features** |  |  |  |  |
| At MAF >= 0.05 |  |  |  |  |
| Sites (with meQTLs) | 683,152 | 3,819 | 286 | 4,544,738 |
| ... with CpG-SNPs | 75% | 45% | 35% | 33% |
| ... with other SNPs | 12% | 18% | 20% | 30% |
| ... without SNPs | 13% | 37% | 45% | 37% |

Table 2. Traits and diseases from the NHGRI GWAS catalog with associated loci enriched for local meQTLs

| **Phenotype** | N annot | Overlap | Fisher PV | OR | Perm Z | Perm PV | Perm QV |
| --- | --- | --- | --- | --- | --- | --- | --- |
| bone mineral density | 25 | 15 | 1.58E-07 | 9.03 | 6.530 | 4.40E-10 | 1.07E-07 |
| breast cancer | 34 | 17 | 8.28E-07 | 6.02 | 5.878 | 2.51E-08 | 3.05E-06 |
| blood pressure | 9 | 7 | 3.28E-05 | 21.06 | 5.551 | 1.63E-07 | 1.31E-05 |
| height | 90 | 31 | 1.25E-06 | 3.16 | 5.499 | 2.16E-07 | 1.31E-05 |
| major depressive disorder | 14 | 9 | 2.44E-05 | 10.84 | 5.452 | 2.80E-07 | 1.36E-05 |
| non-alcoholic fatty liver disease | 7 | 6 | 5.13E-05 | 36.10 | 5.413 | 3.47E-07 | 1.41E-05 |
| Alzheimer's disease | 17 | 10 | 2.54E-05 | 8.60 | 5.203 | 1.06E-06 | 3.67E-05 |
| rheumatoid arthritis | 37 | 16 | 1.84E-05 | 4.59 | 5.167 | 1.27E-06 | 3.86E-05 |
| hypertension | 10 | 7 | 9.59E-05 | 14.05 | 5.010 | 2.82E-06 | 7.62E-05 |
| QRS duration | 8 | 6 | 1.80E-04 | 18.06 | 4.911 | 4.62E-06 | 1.08E-04 |
| Crohn's disease | 42 | 17 | 2.89E-05 | 4.09 | 4.900 | 4.89E-06 | 1.08E-04 |
| pulmonary function decline | 6 | 5 | 3.10E-04 | 30.10 | 4.803 | 7.79E-06 | 1.58E-04 |
| IgG glycosylation | 70 | 23 | 8.67E-05 | 2.95 | 4.409 | 4.80E-05 | 8.98E-04 |
| systemic lupus erythematosus | 15 | 8 | 4.25E-04 | 6.88 | 4.199 | 1.18E-04 | 1.93E-03 |
| bone mineral density - spine | 5 | 4 | 1.82E-03 | 24.08 | 4.198 | 1.19E-04 | 1.93E-03 |
| hip geometry | 5 | 4 | 1.82E-03 | 24.08 | 4.173 | 1.32E-04 | 2.01E-03 |
| autism spectrum, attention deficit-hyperactivity, bipolar and major depressive disorders and schizophrenia combined | 19 | 9 | 5.72E-04 | 5.42 | 4.103 | 1.77E-04 | 2.52E-03 |
| mean corpuscular hemoglobin | 5 | 4 | 1.82E-03 | 24.08 | 4.084 | 1.90E-04 | 2.57E-03 |
| tuberculosis | 8 | 5 | 2.25E-03 | 10.03 | 4.031 | 2.36E-04 | 3.02E-03 |
| bipolar disorder and schizophrenia | 24 | 10 | 9.86E-04 | 4.30 | 3.799 | 5.85E-04 | 7.11E-03 |
| obesity-related traits | 165 | 40 | 7.21E-04 | 1.93 | 3.709 | 8.22E-04 | 9.52E-03 |

**Table 2**. We examined 494,420 meQTLs out of 3,470,923 methylation sites, after removal of sites in regions flagged as CNVs, genomic duplications or pseudogenes. We consider local meQTLs and do not stratify by presence or absence of CpG-SNPs at the meQTL site. "N annot" is the number of loci in the NHGRI GWAS catalog for that trait or disease, "Overlap" is the number of GWAS loci that overlap with a local meQTL, "Fisher PV" is the *p*-value from the Fisher exact test of enrichment, "OR" is the odds ratio, "Perm Z" is the Z-statistic of the permutation test, "Perm PV" is the *p*-value from >300K permutations, "Perm QV" is the *q*-value of the permutation test. Only findings passing FDR control with *q*-value<0.01 are shown.

**References**

1. Reik W, Dean W, Walter J: **Epigenetic reprogramming in mammalian development**. *Science* 2001, **293**:1089–1093.

2. Henikoff S, Matzke MA: **Exploring and explaining epigenetic effects**. *Trends Genet* 1997, **13**:293–295.

3. Berger SL: **The complex language of chromatin regulation during transcription**. *Nature* 2007, **447**:407–412.

4. Feil R, Fraga MF: **Epigenetics and the environment: emerging patterns and implications**. *Nat Rev Genet* 2011, **13**:97–109.

5. Petronis A: **Epigenetics as a unifying principle in the aetiology of complex traits and diseases**. *Nature* 2010, **465**:721–727.

6. Kaminsky ZA, Tang T, Wang S-C, Ptak C, Oh GHT, Wong AHC, Feldcamp LA, Virtanen C, Halfvarson J, Tysk C, McRae AF, Visscher PM, Montgomery GW, Gottesman II, Martin NG, Petronis A: **DNA methylation profiles in monozygotic and dizygotic twins**. *Nat Genet* 2009, **41**:240–245.

7. Bell JT, Spector TD: **DNA methylation studies using twins: what are they telling us?** *Genome Biol* 2012, **13**:172.

8. Meaburn EL, Schalkwyk LC, Mill J: **Allele-specific methylation in the human genome: implications for genetic studies of complex disease**. *Epigenetics* 2010, **5**:578–582.

9. Kerkel K, Spadola A, Yuan E, Kosek J, Jiang L, Hod E, Li K, Murty VV, Schupf N, Vilain E, Morris M, Haghighi F, Tycko B: **Genomic surveys by methylation-sensitive SNP analysis identify sequence-dependent allele-specific DNA methylation**. *Nat Genet* 2008, **40**:904–908.

10. Drong AW, Nicholson G, Hedman AK, Meduri E, Grundberg E, Small KS, Shin S-Y, Bell JT, Karpe F, Soranzo N, Spector TD, McCarthy MI, Deloukas P, Rantalainen M, Lindgren CM, MolPAGE Consortia: **The presence of methylation quantitative trait loci indicates a direct genetic influence on the level of DNA methylation in adipose tissue**. *PLoS ONE* 2013, **8**:e55923.

11. Nelson SC, Doheny KF, Pugh EW, Romm JM, Ling H, Laurie CA, Browning SR, Weir BS, Laurie CC: **Imputation-based genomic coverage assessments of current human genotyping arrays**. *G3 (Bethesda)* 2013, **3**:1795–1807.

12. Laird PW: **Principles and challenges of genomewide DNA methylation analysis**. *Nat Rev Genet* 2010, **11**:191–203.

13. Rakyan VK, Down TA, Balding DJ, Beck S: **Epigenome-wide association studies for common human diseases**. *Nat Rev Genet* 2011, **12**:529–541.

14. Zhang D, Cheng L, Badner JA, Chen C, Chen Q, Luo W, Craig DW, Redman M, Gershon ES, Liu C: **Genetic control of individual differences in gene-specific methylation in human brain**. *Am J Hum Genet* 2010, **86**:411–419.

15. Gibbs JR, van der Brug MP, Hernandez DG, Traynor BJ, Nalls MA, Lai S-L, Arepalli S, Dillman A, Rafferty IP, Troncoso J, Johnson R, Zielke HR, Ferrucci L, Longo DL, Cookson MR, Singleton AB: **Abundant quantitative trait loci exist for DNA methylation and gene expression in human brain**. *PLoS Genet* 2010, **6**:e1000952.

16. Bell JT, Pai AA, Pickrell JK, Gaffney DJ, Pique-Regi R, Degner JF, Gilad Y, Pritchard JK: **DNA methylation patterns associate with genetic and gene expression variation in HapMap cell lines**. *Genome Biol* 2011, **12**:R10.

17. Shi J, Marconett CN, Duan J, Hyland PL, Li P, Wang Z, Wheeler W, Zhou B, Campan M, Lee DS, Huang J, Zhou W, Triche T, Amundadottir L, Warner A, Hutchinson A, Chen P-H, Chung BSI, Pesatori AC, Consonni D, Bertazzi PA, Bergen AW, Freedman M, Siegmund KD, Berman BP, Borok Z, Chatterjee N, Tucker MA, Caporaso NE, Chanock SJ, et al.: **Characterizing the genetic basis of methylome diversity in histologically normal human lung tissue**. *Nat Commun* 2014, **5**:3365.

18. Roadmap Epigenomics Consortium, Kundaje A, Meuleman W, Ernst J, Bilenky M, Yen A, Heravi-Moussavi A, Kheradpour P, Zhang Z, Wang J, Ziller MJ, Amin V, Whitaker JW, Schultz MD, Ward LD, Sarkar A, Quon G, Sandstrom RS, Eaton ML, Wu Y-C, Pfenning AR, Wang X, Claussnitzer M, Liu Y, Coarfa C, Harris RA, Shoresh N, Epstein CB, Gjoneska E, Leung D, et al.: **Integrative analysis of 111 reference human epigenomes**. *Nature* 2015, **518**:317–330.

19. Irizarry RA, Ladd-Acosta C, Wen B, Wu Z, Montano C, Onyango P, Cui H, Gabo K, Rongione M, Webster M, Ji H, Potash JB, Sabunciyan S, Feinberg AP: **The human colon cancer methylome shows similar hypo- and hypermethylation at conserved tissue-specific CpG island shores**. *Nat Genet* 2009, **41**:178–186.

20. Harris RA, Wang T, Coarfa C, Nagarajan RP, Hong C, Downey SL, Johnson BE, Fouse SD, Delaney A, Zhao Y, Olshen A, Ballinger T, Zhou X, Forsberg KJ, Gu J, Echipare L, O’Geen H, Lister R, Pelizzola M, Xi Y, Epstein CB, Bernstein BE, Hawkins RD, Ren B, Chung W-Y, Gu H, Bock C, Gnirke A, Zhang MQ, Haussler D, et al.: **Comparison of sequencing-based methods to profile DNA methylation and identification of monoallelic epigenetic modifications**. *Nat Biotechnol* 2010, **28**:1097–1105.

21. Serre D, Lee BH, Ting AH: **MBD-isolated Genome Sequencing provides a high-throughput and comprehensive survey of DNA methylation in the human genome**. *Nucleic Acids Res* 2010, **38**:391–399.

22. Li N, Ye M, Li Y, Yan Z, Butcher LM, Sun J, Han X, Chen Q, Zhang X, Wang J: **Whole genome DNA methylation analysis based on high throughput sequencing technology**. *Methods* 2010, **52**:203–212.

23. Hogart A, Lichtenberg J, Ajay SS, Anderson S, NIH Intramural Sequencing Center, Margulies EH, Bodine DM: **Genome-wide DNA methylation profiles in hematopoietic stem and progenitor cells reveal overrepresentation of ETS transcription factor binding sites**. *Genome Res* 2012, **22**:1407–1418.

24. Lan X, Adams C, Landers M, Dudas M, Krissinger D, Marnellos G, Bonneville R, Xu M, Wang J, Huang TH-M, Meredith G, Jin VX: **High resolution detection and analysis of CpG dinucleotides methylation using MBD-Seq technology**. *PLoS ONE* 2011, **6**:e22226.

25. Nair SS, Coolen MW, Stirzaker C, Song JZ, Statham AL, Strbenac D, Robinson MD, Clark SJ: **Comparison of methyl-DNA immunoprecipitation (MeDIP) and methyl-CpG binding domain (MBD) protein capture for genome-wide DNA methylation analysis reveal CpG sequence coverage bias**. *Epigenetics* 2011, **6**:34–44.

26. McClay JL, Aberg KA, Clark SL, Nerella S, Kumar G, Xie LY, Hudson AD, Harada A, Hultman CM, Magnusson PKE, Sullivan PF, Van Den Oord EJCG: **A methylome-wide study of aging using massively parallel sequencing of the methyl-CpG-enriched genomic fraction from blood in over 700 subjects**. *Hum Mol Genet* 2014, **23**:1175–1185.

27. Aberg KA, McClay JL, Nerella S, Clark S, Kumar G, Chen W, Khachane AN, Xie L, Hudson A, Gao G, Harada A, Hultman CM, Sullivan PF, Magnusson PKE, van den Oord EJCG: **Methylome-wide association study of schizophrenia: identifying blood biomarker signatures of environmental insults**. *JAMA Psychiatry* 2014, **71**:255–264.

28. Howie B, Fuchsberger C, Stephens M, Marchini J, Abecasis GR: **Fast and accurate genotype imputation in genome-wide association studies through pre-phasing**. *Nat Genet* 2012, **44**:955–959.

29. Bergen SE, O’Dushlaine CT, Ripke S, Lee PH, Ruderfer DM, Akterin S, Moran JL, Chambert KD, Handsaker RE, Backlund L, Ösby U, McCarroll S, Landen M, Scolnick EM, Magnusson PKE, Lichtenstein P, Hultman CM, Purcell SM, Sklar P, Sullivan PF: **Genome-wide association study in a Swedish population yields support for greater CNV and MHC involvement in schizophrenia compared with bipolar disorder**. *Mol Psychiatry* 2012, **17**:880–886.

30. Ripke S, O’Dushlaine C, Chambert K, Moran JL, Kähler AK, Akterin S, Bergen SE, Collins AL, Crowley JJ, Fromer M, Kim Y, Lee SH, Magnusson PKE, Sanchez N, Stahl EA, Williams S, Wray NR, Xia K, Bettella F, Borglum AD, Bulik-Sullivan BK, Cormican P, Craddock N, de Leeuw C, Durmishi N, Gill M, Golimbet V, Hamshere ML, Holmans P, Hougaard DM, et al.: **Genome-wide association analysis identifies 13 new risk loci for schizophrenia**. *Nat Genet* 2013, **45**:1150–1159.

31. Aberg KA, McClay JL, Nerella S, Xie LY, Clark SL, Hudson AD, Bukszár J, Adkins D, Consortium SS, Hultman CM, Sullivan PF, Magnusson PKE, van den Oord EJCG: **MBD-seq as a cost-effective approach for methylome-wide association studies: demonstration in 1500 case--control samples**. *Epigenomics* 2012, **4**:605–621.

32. Chen W, Gao G, Nerella S, Hultman CM, Magnusson PKE, Sullivan PF, Aberg KA, van den Oord EJCG: **MethylPCA: a toolkit to control for confounders in methylome-wide association studies**. *BMC Bioinformatics* 2013, **14**:74.

33. van den Oord EJCG, Bukszar J, Rudolf G, Nerella S, McClay JL, Xie LY, Aberg KA: **Estimation of CpG coverage in whole methylome next-generation sequencing studies**. *BMC Bioinformatics* 2013, **14**:50.

34. International Human Genome Sequencing Consortium: **Finishing the euchromatic sequence of the human genome**. *Nature* 2004, **431**:931–945.

35. Shabalin AA: **Matrix eQTL: ultra fast eQTL analysis via large matrix operations**. *Bioinformatics* 2012, **28**:1353–1358.

36. Benjamini Y, Hochberg Y: **On the Adaptive Control of the False Discovery Rate in Multiple Testing With Independent Statistics**. *JOURNAL OF EDUCATIONAL AND BEHAVIORAL STATISTICS* 2000, **25**:60–83.

37. Zhi D, Aslibekyan S, Irvin MR, Claas SA, Borecki IB, Ordovas JM, Absher DM, Arnett DK: **SNPs located at CpG sites modulate genome-epigenome interaction**. *Epigenetics* 2013, **8**:802–806.

38. ENCODE Project Consortium: **An integrated encyclopedia of DNA elements in the human genome**. *Nature* 2012, **489**:57–74.

39. Schalkwyk LC, Meaburn EL, Smith R, Dempster EL, Jeffries AR, Davies MN, Plomin R, Mill J: **Allelic skewing of DNA methylation is widespread across the genome**. *Am J Hum Genet* 2010, **86**:196–212.

40. Shoemaker R, Deng J, Wang W, Zhang K: **Allele-specific methylation is prevalent and is contributed by CpG-SNPs in the human genome**. *Genome Res* 2010, **20**:883–889.

41. Zeggini E, Weedon MN, Lindgren CM, Frayling TM, Elliott KS, Lango H, Timpson NJ, Perry JRB, Rayner NW, Freathy RM, Barrett JC, Shields B, Morris AP, Ellard S, Groves CJ, Harries LW, Marchini JL, Owen KR, Knight B, Cardon LR, Walker M, Hitman GA, Morris AD, Doney ASF, (wtccc) TWTCCC, McCarthy MI, Hattersley AT: **Replication of Genome-Wide Association Signals in UK Samples Reveals Risk Loci for Type 2 Diabetes**. *Science* 2007, **316**:1336–1341.

42. Dayeh TA, Olsson AH, Volkov P, Almgren P, Rönn T, Ling C: **Identification of CpG-SNPs associated with type 2 diabetes and differential DNA methylation in human pancreatic islets**. *Diabetologia* 2013, **56**:1036–1046.

43. Ernst J, Kheradpour P, Mikkelsen TS, Shoresh N, Ward LD, Epstein CB, Zhang X, Wang L, Issner R, Coyne M, Ku M, Durham T, Kellis M, Bernstein BE: **Mapping and analysis of chromatin state dynamics in nine human cell types**. *Nature* 2011, **473**:43–49.

44. Maurano MT, Humbert R, Rynes E, Thurman RE, Haugen E, Wang H, Reynolds AP, Sandstrom R, Qu H, Brody J, Shafer A, Neri F, Lee K, Kutyavin T, Stehling-Sun S, Johnson AK, Canfield TK, Giste E, Diegel M, Bates D, Hansen RS, Neph S, Sabo PJ, Heimfeld S, Raubitschek A, Ziegler S, Cotsapas C, Sotoodehnia N, Glass I, Sunyaev SR, et al.: **Systematic localization of common disease-associated variation in regulatory DNA**. *Science* 2012, **337**:1190–1195.

45. Brandeis M, Frank D, Keshet I, Siegfried Z, Mendelsohn M, Nemes A, Temper V, Razin A, Cedar H: **Sp1 elements protect a CpG island from de novo methylation**. *Nature* 1994, **371**:435–438.

46. Schizophrenia Psychiatric Genome-Wide Association Study (GWAS) Consortium: **Genome-wide association study identifies five new schizophrenia loci**. *Nat Genet* 2011, **43**:969–976.

47. International Schizophrenia Consortium, Purcell SM, Wray NR, Stone JL, Visscher PM, O’Donovan MC, Sullivan PF, Sklar P: **Common polygenic variation contributes to risk of schizophrenia and bipolar disorder**. *Nature* 2009, **460**:748–752.

48. McKernan KJ, Peckham HE, Costa GL, McLaughlin SF, Fu Y, Tsung EF, Clouser CR, Duncan C, Ichikawa JK, Lee CC, Zhang Z, Ranade SS, Dimalanta ET, Hyland FC, Sokolsky TD, Zhang L, Sheridan A, Fu H, Hendrickson CL, Li B, Kotler L, Stuart JR, Malek JA, Manning JM, Antipova AA, Perez DS, Moore MP, Hayashibara KC, Lyons MR, Beaudoin RE, et al.: **Sequence and structural variation in a human genome uncovered by short-read, massively parallel ligation sequencing using two-base encoding**. *Genome Res* 2009, **19**:1527–1541.

49. Bock C, Tomazou EM, Brinkman AB, Müller F, Simmer F, Gu H, Jäger N, Gnirke A, Stunnenberg HG, Meissner A: **Quantitative comparison of genome-wide DNA methylation mapping technologies**. *Nat Biotechnol* 2010, **28**:1106–1114.

50. Chavez L, Jozefczuk J, Grimm C, Dietrich J, Timmermann B, Lehrach H, Herwig R, Adjaye J: **Computational analysis of genome-wide DNA methylation during the differentiation of human embryonic stem cells along the endodermal lineage**. *Genome Res* 2010, **20**:1441–1450.

51. Down TA, Rakyan VK, Turner DJ, Flicek P, Li H, Kulesha E, Gräf S, Johnson N, Herrero J, Tomazou EM, Thorne NP, Bäckdahl L, Herberth M, Howe KL, Jackson DK, Miretti MM, Marioni JC, Birney E, Hubbard TJP, Durbin R, Tavaré S, Beck S: **A Bayesian deconvolution strategy for immunoprecipitation-based DNA methylome analysis**. *Nat Biotechnol* 2008, **26**:779–785.

52. Åberg K, Khachane AN, Rudolf G, Nerella S, Fugman DA, Tischfield JA, van den Oord EJCG: **Methylome-wide comparison of human genomic DNA extracted from whole blood and from EBV-transformed lymphocyte cell lines**. *Eur J Hum Genet* 2012, **20**:953–955.

53. Wright FA, Sullivan PF, Brooks AI, Zou F, Sun W, Xia K, Madar V, Jansen R, Chung W, Zhou Y-H, Abdellaoui A, Batista S, Butler C, Chen G, Chen T-H, D’Ambrosio D, Gallins P, Ha MJ, Hottenga JJ, Huang S, Kattenberg M, Kochar J, Middeldorp CM, Qu A, Shabalin A, Tischfield J, Todd L, Tzeng J-Y, van Grootheest G, Vink JM, et al.: **Heritability and genomics of gene expression in peripheral blood**. *Nat Genet* 2014, **46**:430–437.

54. Houseman EA, Accomando WP, Koestler DC, Christensen BC, Marsit CJ, Nelson HH, Wiencke JK, Kelsey KT: **DNA methylation arrays as surrogate measures of cell mixture distribution**. *BMC Bioinformatics* 2012, **13**:86.

55. Liu Y, Aryee MJ, Padyukov L, Fallin MD, Hesselberg E, Runarsson A, Reinius L, Acevedo N, Taub M, Ronninger M, Shchetynsky K, Scheynius A, Kere J, Alfredsson L, Klareskog L, Ekström TJ, Feinberg AP: **Epigenome-wide association data implicate DNA methylation as an intermediary of genetic risk in rheumatoid arthritis**. *Nat Biotechnol* 2013, **31**:142–147.

56. Sun YV, Turner ST, Smith JA, Hammond PI, Lazarus A, Van De Rostyne JL, Cunningham JM, Kardia SLR: **Comparison of the DNA methylation profiles of human peripheral blood cells and transformed B-lymphocytes**. *Hum Genet* 2010, **127**:651–658.

57. Benjamini Y, Hochberg Y: **Controlling the False Discovery Rate: A Practical and Powerful Approach to Multiple Testing**. *Journal of the Royal Statistical Society Series B (Methodological)* 1995, **57**:289–300.

58. Gerstein MB, Kundaje A, Hariharan M, Landt SG, Yan K-K, Cheng C, Mu XJ, Khurana E, Rozowsky J, Alexander R, Min R, Alves P, Abyzov A, Addleman N, Bhardwaj N, Boyle AP, Cayting P, Charos A, Chen DZ, Cheng Y, Clarke D, Eastman C, Euskirchen G, Frietze S, Fu Y, Gertz J, Grubert F, Harmanci A, Jain P, Kasowski M, et al.: **Architecture of the human regulatory network derived from ENCODE data**. *Nature* 2012, **489**:91–100.

59. Wang J, Zhuang J, Iyer S, Lin X, Whitfield TW, Greven MC, Pierce BG, Dong X, Kundaje A, Cheng Y, Rando OJ, Birney E, Myers RM, Noble WS, Snyder M, Weng Z: **Sequence features and chromatin structure around the genomic regions bound by 119 human transcription factors**. *Genome Res* 2012, **22**:1798–1812.

60. Pennacchio LA, Ahituv N, Moses AM, Prabhakar S, Nobrega MA, Shoukry M, Minovitsky S, Dubchak I, Holt A, Lewis KD, Plajzer-Frick I, Akiyama J, De Val S, Afzal V, Black BL, Couronne O, Eisen MB, Visel A, Rubin EM: **In vivo enhancer analysis of human conserved non-coding sequences**. *Nature* 2006, **444**:499–502.

61. Huppert JL, Balasubramanian S: **Prevalence of quadruplexes in the human genome**. *Nucleic Acids Res* 2005, **33**:2908–2916.

62. Hindorff LA, Sethupathy P, Junkins HA, Ramos EM, Mehta JP, Collins FS, Manolio TA: **Potential etiologic and functional implications of genome-wide association loci for human diseases and traits**. *Proc Natl Acad Sci USA* 2009, **106**:9362–9367.

63. Dozmorov MG, Cara LR, Giles CB, Wren JD: **GenomeRunner: automating genome exploration**. *Bioinformatics* 2012, **28**:419–420.

64. Rosenbloom KR, Sloan CA, Malladi VS, Dreszer TR, Learned K, Kirkup VM, Wong MC, Maddren M, Fang R, Heitner SG, Lee BT, Barber GP, Harte RA, Diekhans M, Long JC, Wilder SP, Zweig AS, Karolchik D, Kuhn RM, Haussler D, Kent WJ: **ENCODE data in the UCSC Genome Browser: year 5 update**. *Nucleic Acids Res* 2013, **41**(Database issue):D56–63.
