# Supplementary material for: High density methylation QTL analysis in human blood via next-generation sequencing of the methylated genomic DNA fraction
Source: Genome Biol. 2015 Dec 23;16:291. doi: 10.1186/s13059-015-0842-7 (PMC4699364; doi:10.1186/s13059-015-0842-7)
Supplement: Supplementary file 1 — Supplementary Materials. (ZIP 58629 kb) [file 13059_2015_842_MOESM1_ESM.zip › Supplementary Material meQTL.pdf]

SUPPLEMENTARY MATERIAL FOR THE PAPER:

High density methylation QTL survey in human blood via next-generation sequencing of the methylated genomic DNA fraction

Contents

SAMPLE 2

TABLE S1. DESCRIPTIVE STATISTICS OF SAMPLE

PRINCIPAL COMPONENTS (PCS): 3

FIGURE S1. SCREE PLOT.

GENOME-WIDE ASSOCIATION ANALYSIS OF METHYLATION PC LOADINGS 4

FIGURES S2: QUANTILE-QUANTILE PLOTS OF GWAS RESULTS FOR EACH PC

GENETIC VARIATION AT METHYLATION SITES 5

TABLE S2: PROPORTION OF METHYLATION SITES WITH SNPS WITHIN BOUNDARY, STRATIFIED BY MEQTL EFFECT AND SNP MINOR ALLELE FREQUENCY THRESHOLD

DISTRIBUTION OF MEQTLS BY CHROMOSOME 6

CHROMSOME PLOTS OF SNP-METHYLATION ASSOCIATIONS

REFERENCES 28

Supplementary **Tables S3** – **S8** are available as separate spreadsheets in Microsoft Excel (.xlsx) format for download

TABLE S3: Phase I enrichment permutation tests
TABLE S4: Sites overlapping NHGRI GWAS catalog
TABLE S5: Specific Transcription factors whose binding sites are enriched in local meQTLs without CpG-SNPs
TABLE S6: Enrichment by chromatin state for local meQTLs with and without CpG-SNPs
TABLE S7: Enrichment by chromatin state for local meQTLs with CpG-SNPs overlapping the NHGRI GWAS catalog
TABLE S8: All meQTLs (position, CpG-SNPs, genes, other annotations - large file ~62 Mb)

Sample

Our original DNA methylation study sample comprised 1459 subjects, including both schizophrenia case and control subjects[1], who were identified from national population registers in Sweden. They were interviewed about medical conditions and visited their family doctor or local hospital laboratory for blood donation. Those individuals with genome-wide genotype data, collected as described previously[2-3], were used for the current study on methylation quantitative trait loci (meQTLs). The control sample was used in our primary meQTL analysis, while the schizophrenia case sample was used to replicate meQTL findings. Descriptive statistics for both groups are provided in Table S1 below.

Table S1. Descriptive statistics of sample

	Primary sample (controls)			Replication sample (cases)		
	Frequency	%	Total n responding	Frequency	%	Total n responding
Males	377	54.1	697	395	55.6	711
Finnish Ancestors	43	7.1	603	58	10.1	572
Use Alcohol	562	93.8	599	352	58.3	604
Smokes	53	52.5	101	64	49.2	130
Use Narcotics	47	6.8	695	182	26.0	700
Age at Sampling (Mean, SD)	55.2	11.8	697	53.2	11.5	709

Principal Components:

Figure S1. Scree plot.

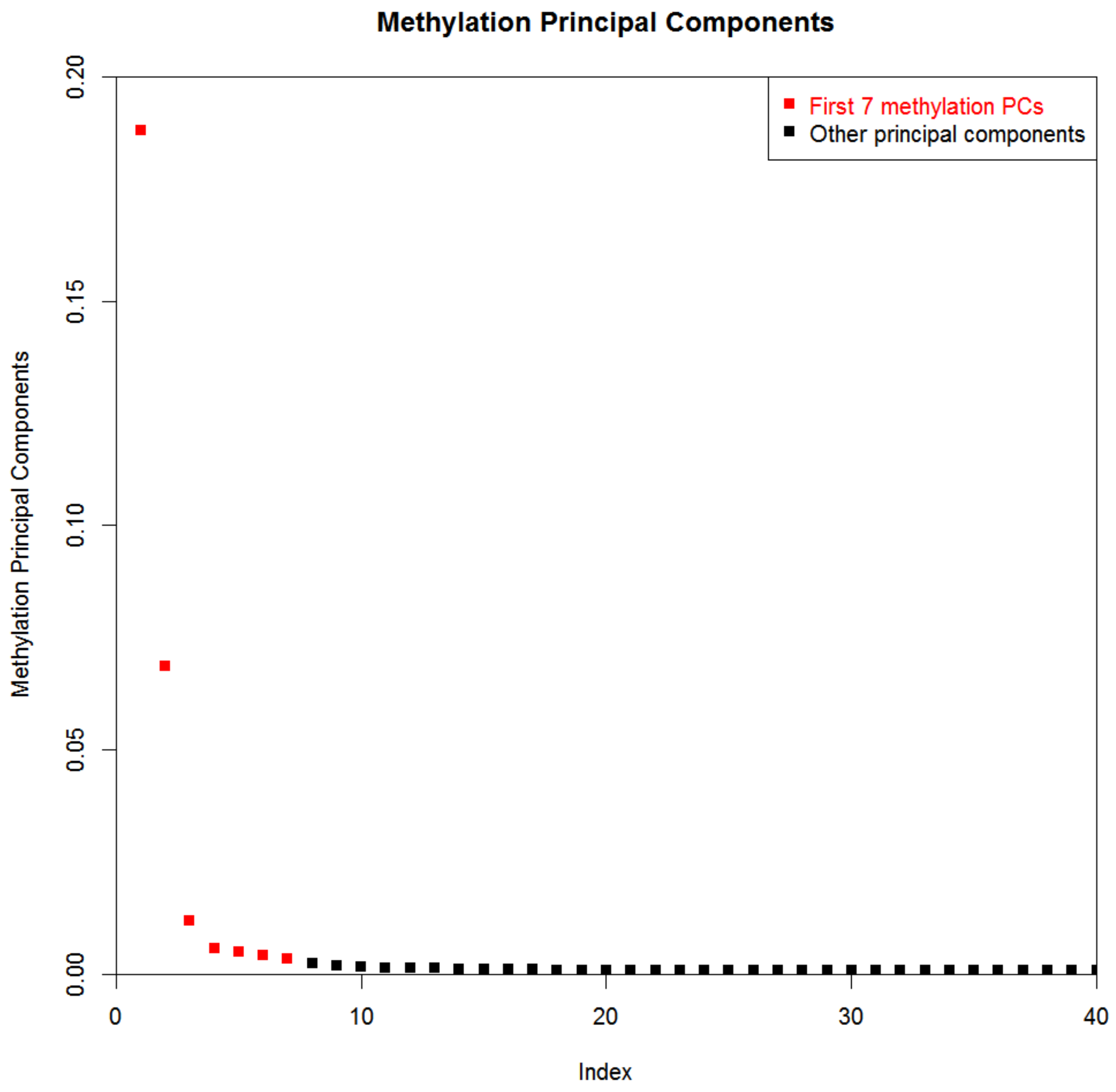

Controlling for confounders to avoid false positive findings presents a major challenge in methylome-wide studies. In addition to technical factors associated with processing samples, there are many possible differences between individuals that may affect the methylome and consequently produce significant association results. Although these effects may be real, they will not be of relevance to the outcome being studied. Statistical methods that first capture the major sources of variation in the methylome, and then regress out these components when performing the analysis may provide an effective solution for handling confounders. We chose PCA for this purpose because it is well developed and commonly used in high-dimensional biological investigations [4-5], including methylation studies[1, 6-10]. FigureS1 shows the scree plot. Based on these results, the first seven PCs were included as covariates in our study.

Genome-wide association analysis of Principal Component loadings

The following Figure shows quantile-quantile (QQ) plots for GWAS with each of the seven principal components (PCs) obtained from our methylation data (Supplementary Figure S1). *P*-values are plotted on a negative logarithmic scale, with observed values on the y-axis and expected values on the x-axis. Although some points showed modest deviation from the null, none survived correction for multiple testing.

Figure S2. QQ Plot GWAS PC1-PC7

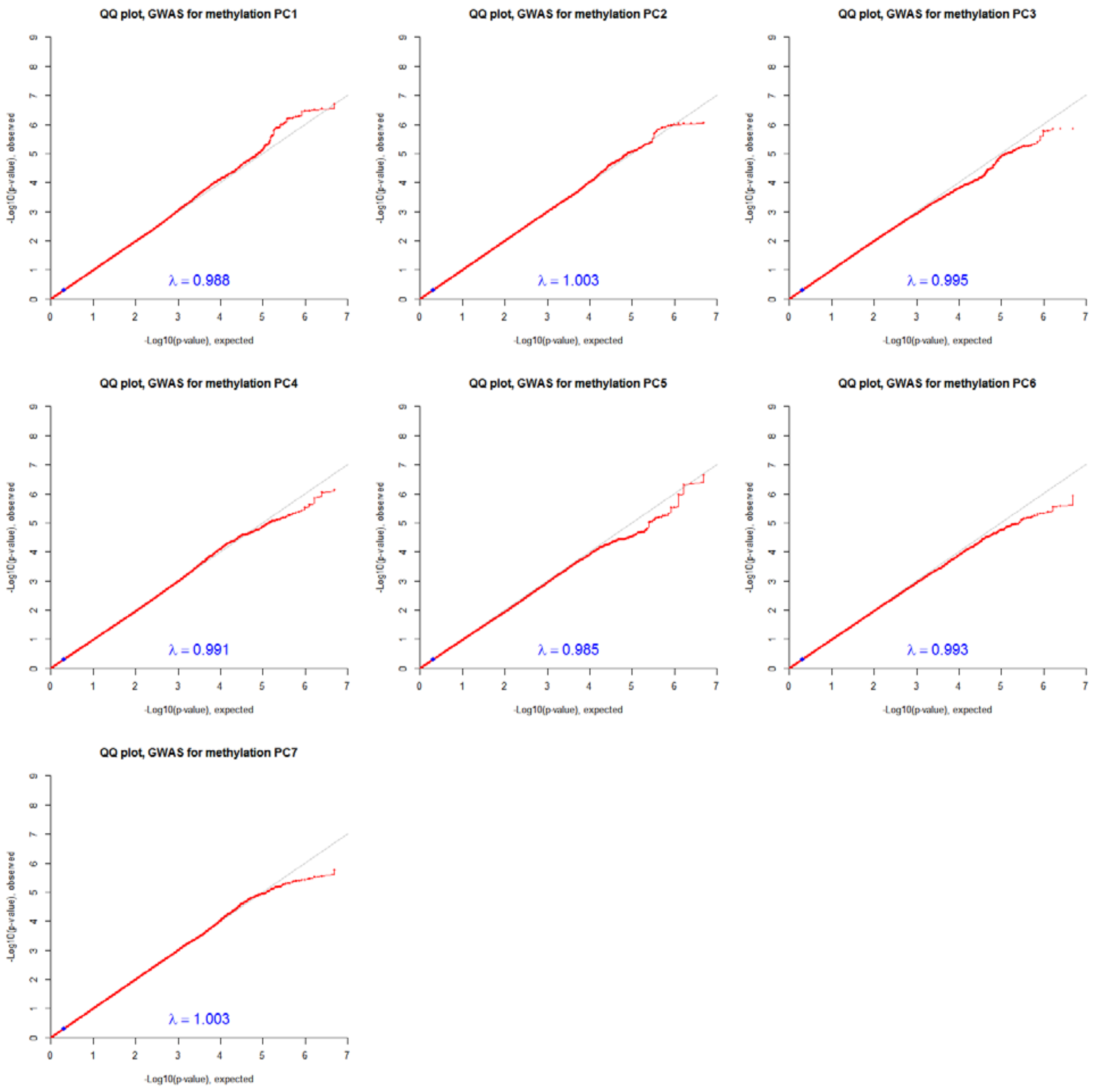

Genetic variation at methylation sites

Table S2: Proportion of methylation sites encompassing SNPs (including 250 bp flanking region), stratified by meQTL effects and minor allele frequency threshold

	Local (<1Mb)	Distant within chromosome (>1Mb)	Cross chromosome	From the total of
At **MAF >= 0**				
Sites (with meQTLs)	683,152	3,819	286	4,544,738
... with CpG-SNPs	90%	65%	63%	67%
... with other SNPs	8%	16%	27%	27%
... without SNPs	2%	19%	10%	6%
At **MAF >= 0.01**				
Sites (with meQTLs)	683,152	3,819	286	4,544,738
... with CpG-SNPs	84%	54%	44%	45%
... with other SNPs	10%	18%	25%	33%
... without SNPs	6%	28%	31%	22%
At **MAF >= 0.03**				
Sites (with meQTLs)	683,152	3,819	286	4,544,738
... with CpG-SNPs	80%	49%	38%	37%
... with other SNPs	11%	18%	22%	32%
... without SNPs	9%	33%	40%	31%
At **MAF >= 0.05**				
Sites (with meQTLs)	683,152	3,819	286	4,544,738
... with CpG-SNPs	75%	45%	35%	33%
... with other SNPs	12%	18%	20%	30%
... without SNPs	13%	37%	45%	37%

Distribution of meQTLs per chromosome

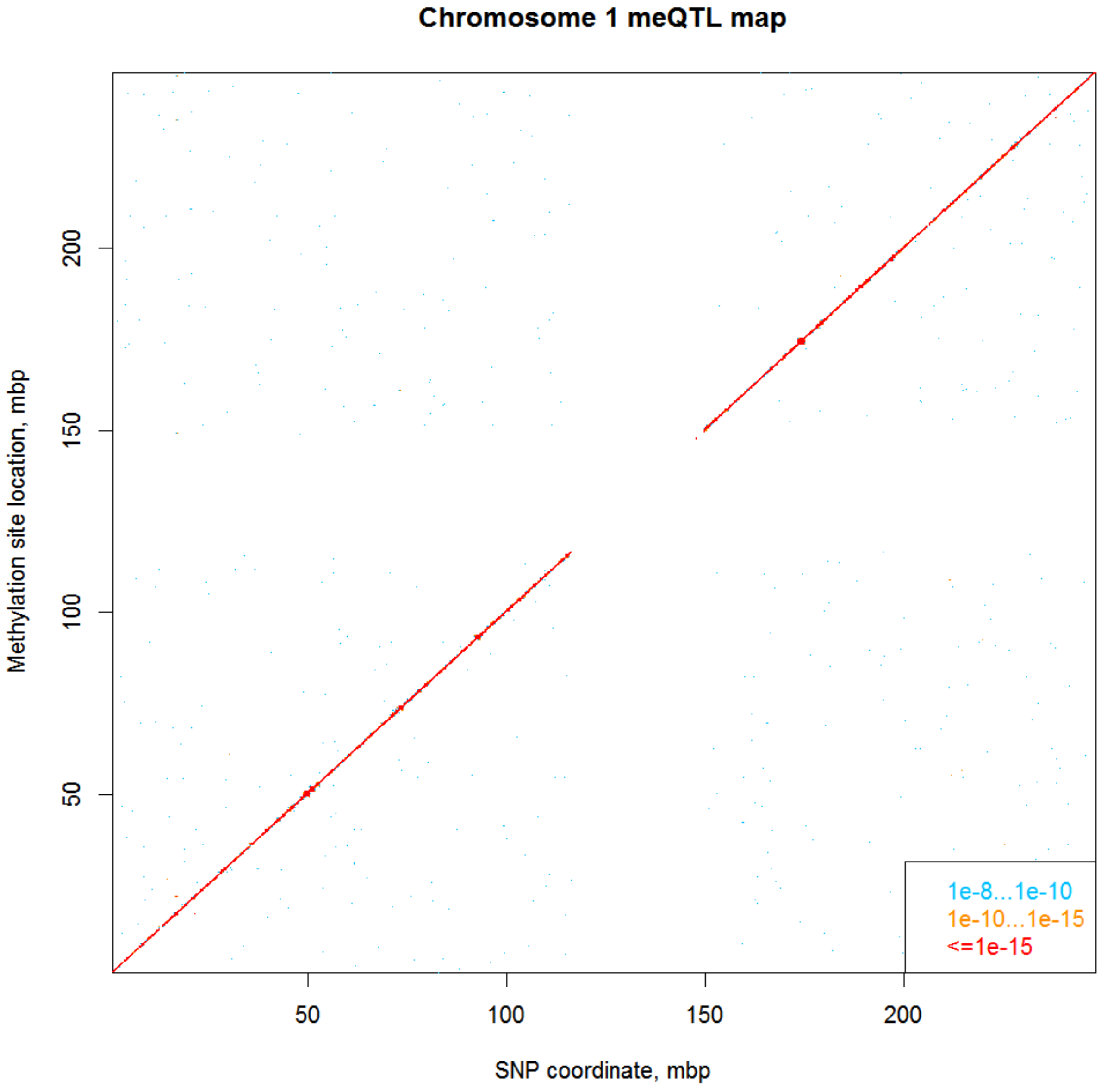

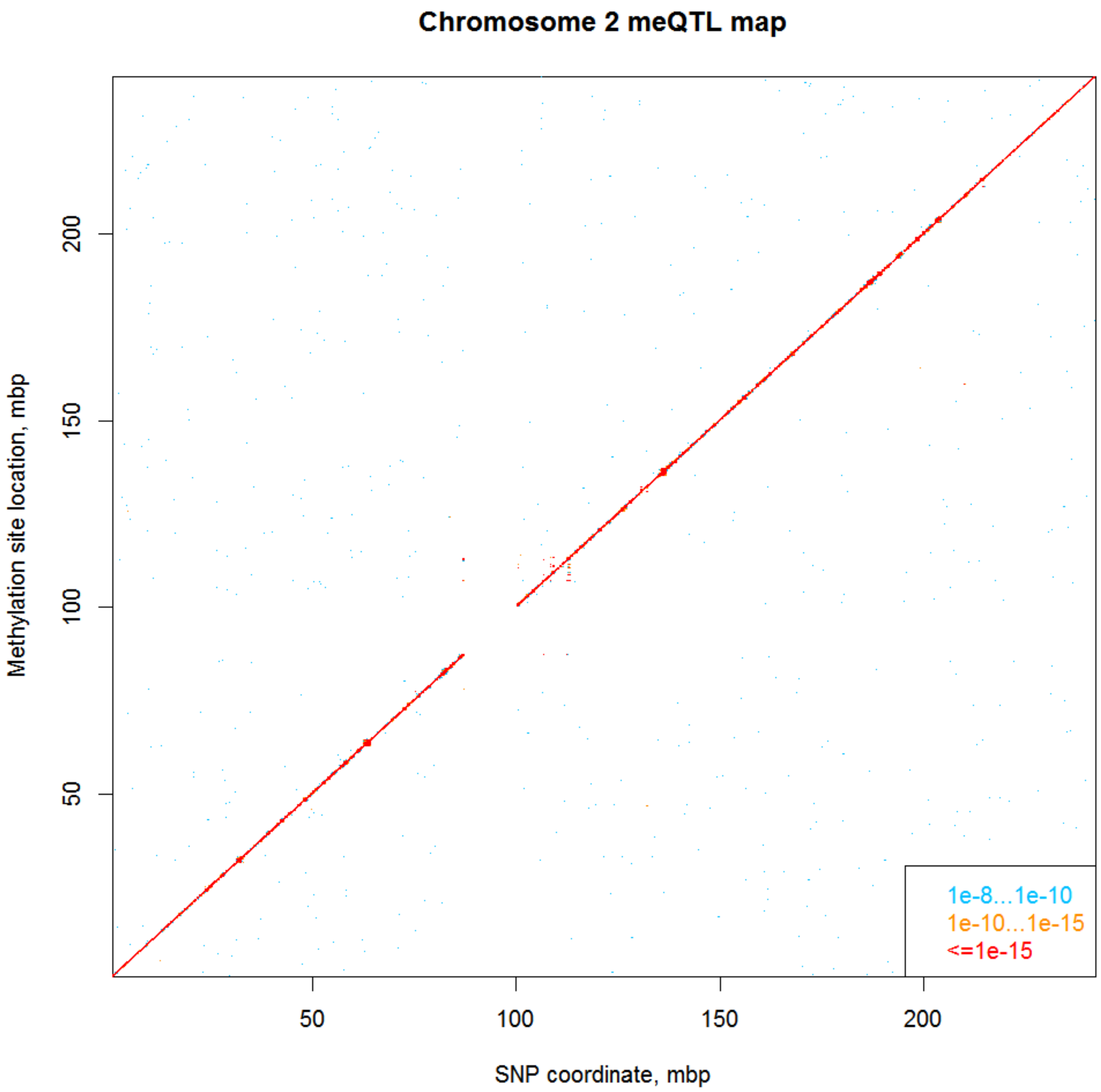
Chromosome 2 meQTL map
Methylation site location, mbp
50
100
150
200
SNP coordinate, mbp
50
100
150
200
1e-8...1e-10
1e-10...1e-15
<=1e-15

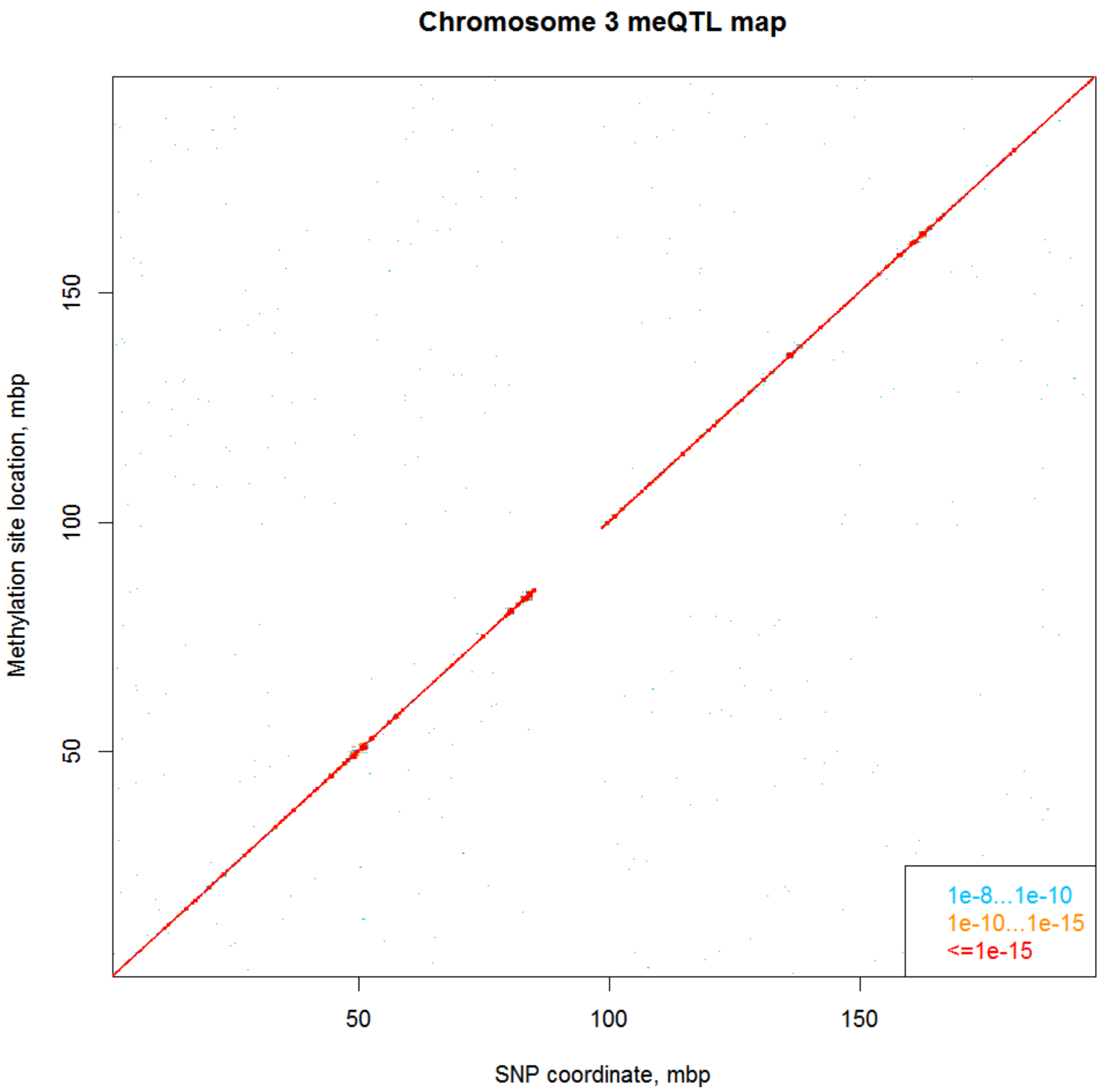
Chromosome 3 meQTL map
Methylation site location, mbp
50
100
150
SNP coordinate, mbp
50
100
150
1e-8...1e-10
1e-10...1e-15
<=1e-15

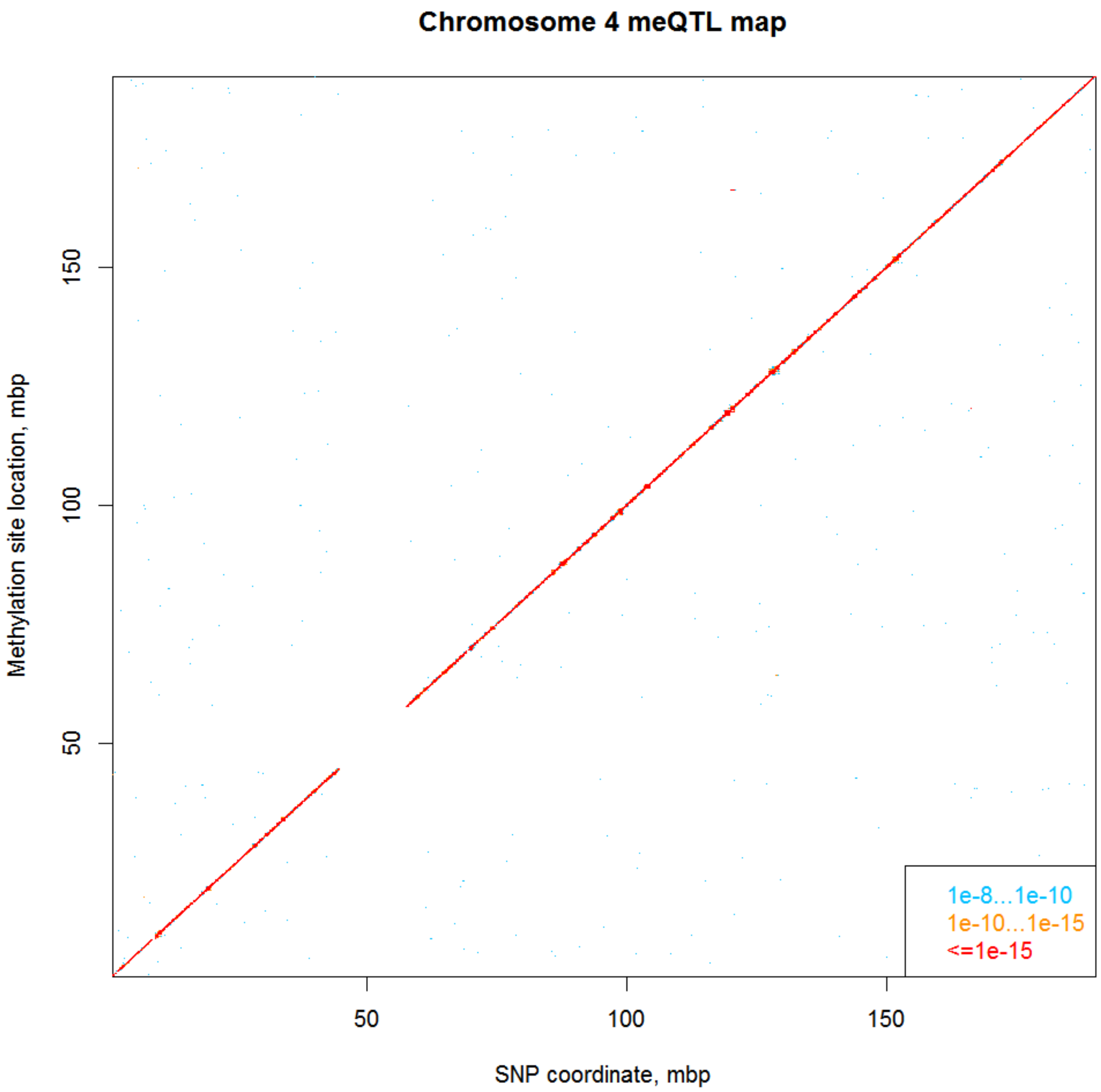
Chromosome 4 meQTL map
Methylation site location, mbp
50
100
150
SNP coordinate, mbp
50
100
150
1e-8...1e-10
1e-10...1e-15
<=1e-15

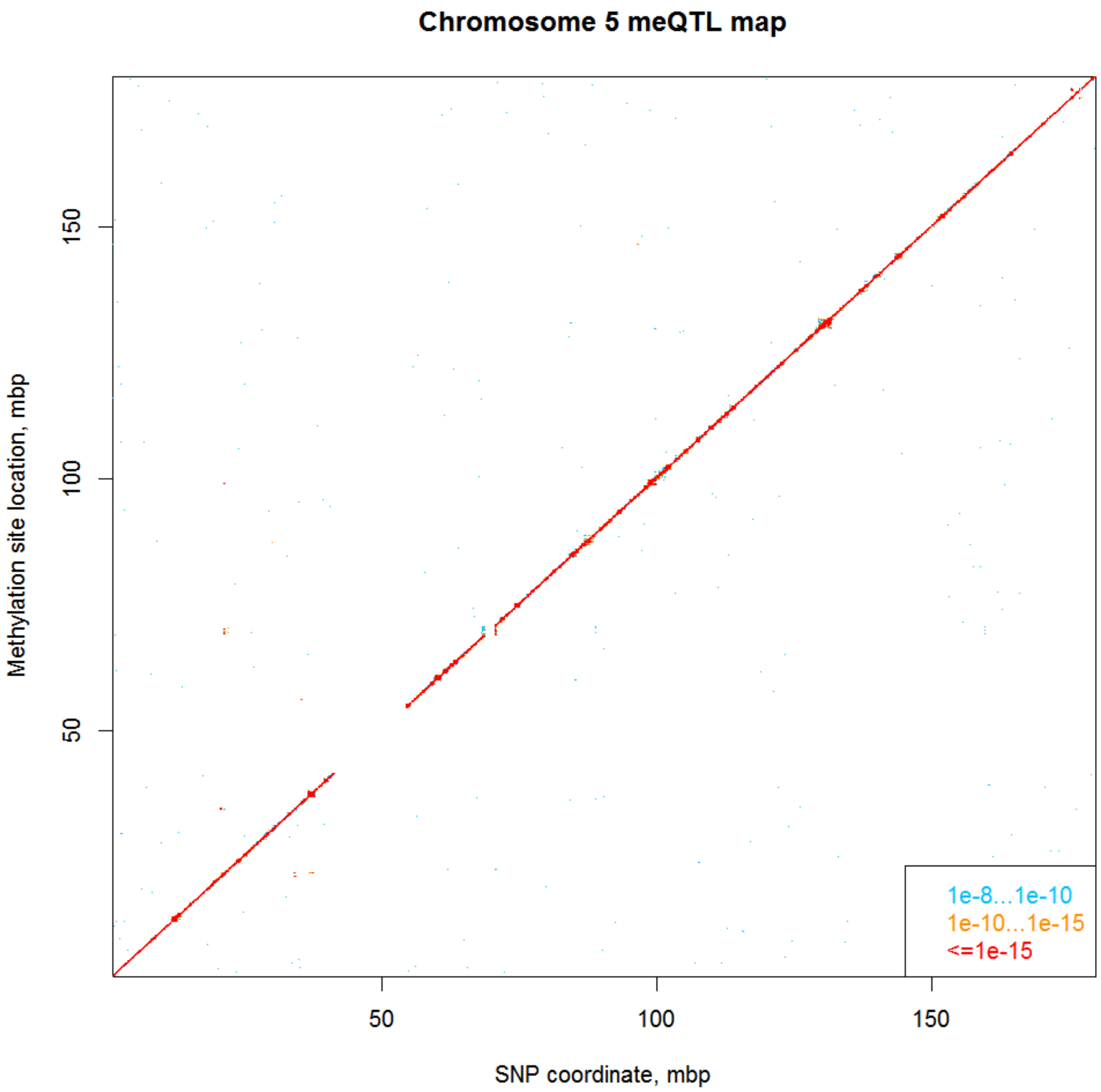

Chromosome 5 meQTL map
Methylation site location, mbp
50
100
150
SNP coordinate, mbp
50
100
150
1e-8...1e-10
1e-10...1e-15
<=1e-15

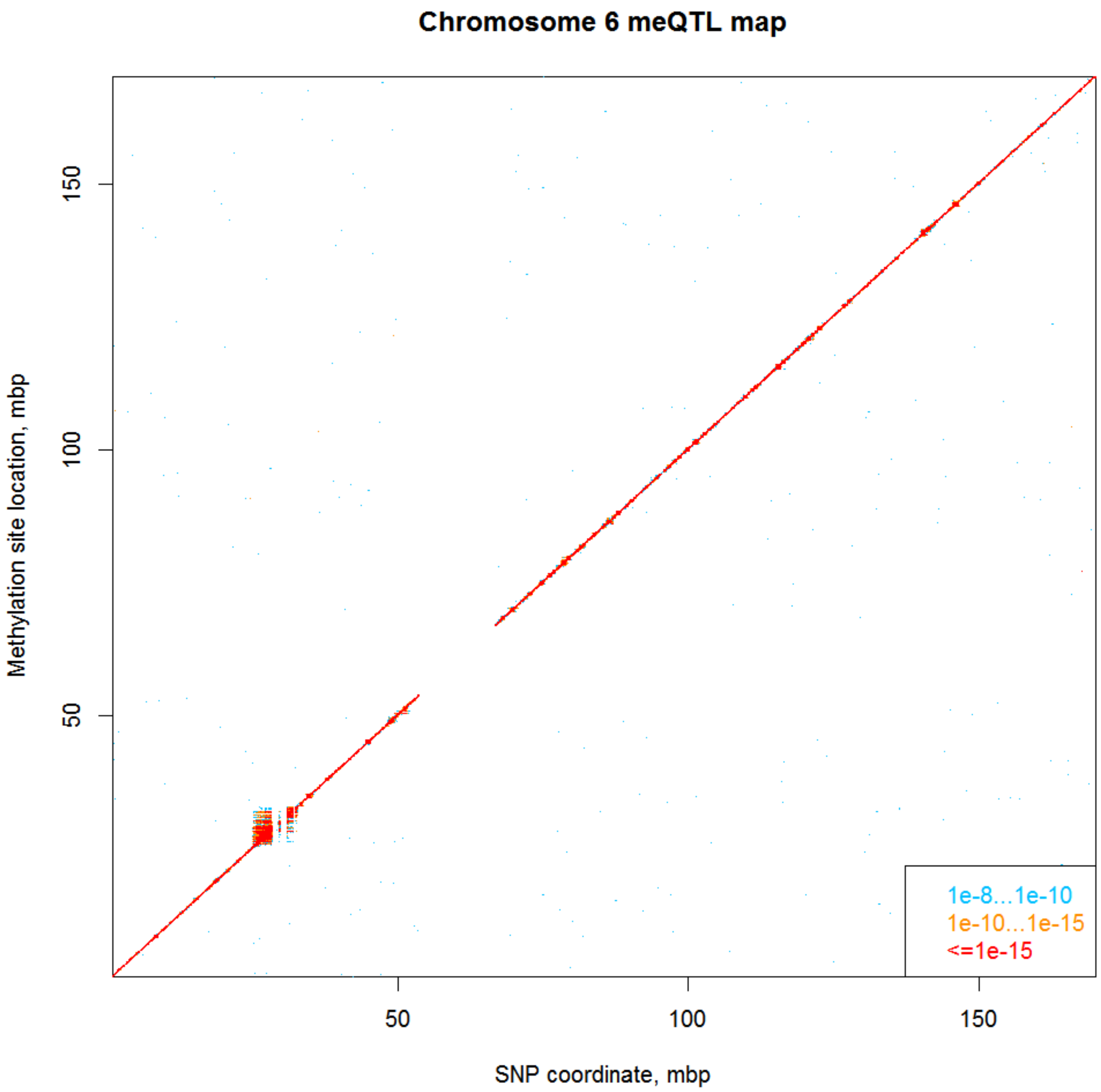
Chromosome 6 meQTL map
Methylation site location, mbp
50
100
150
SNP coordinate, mbp
50
100
150
1e-8...1e-10
1e-10...1e-15
<=1e-15

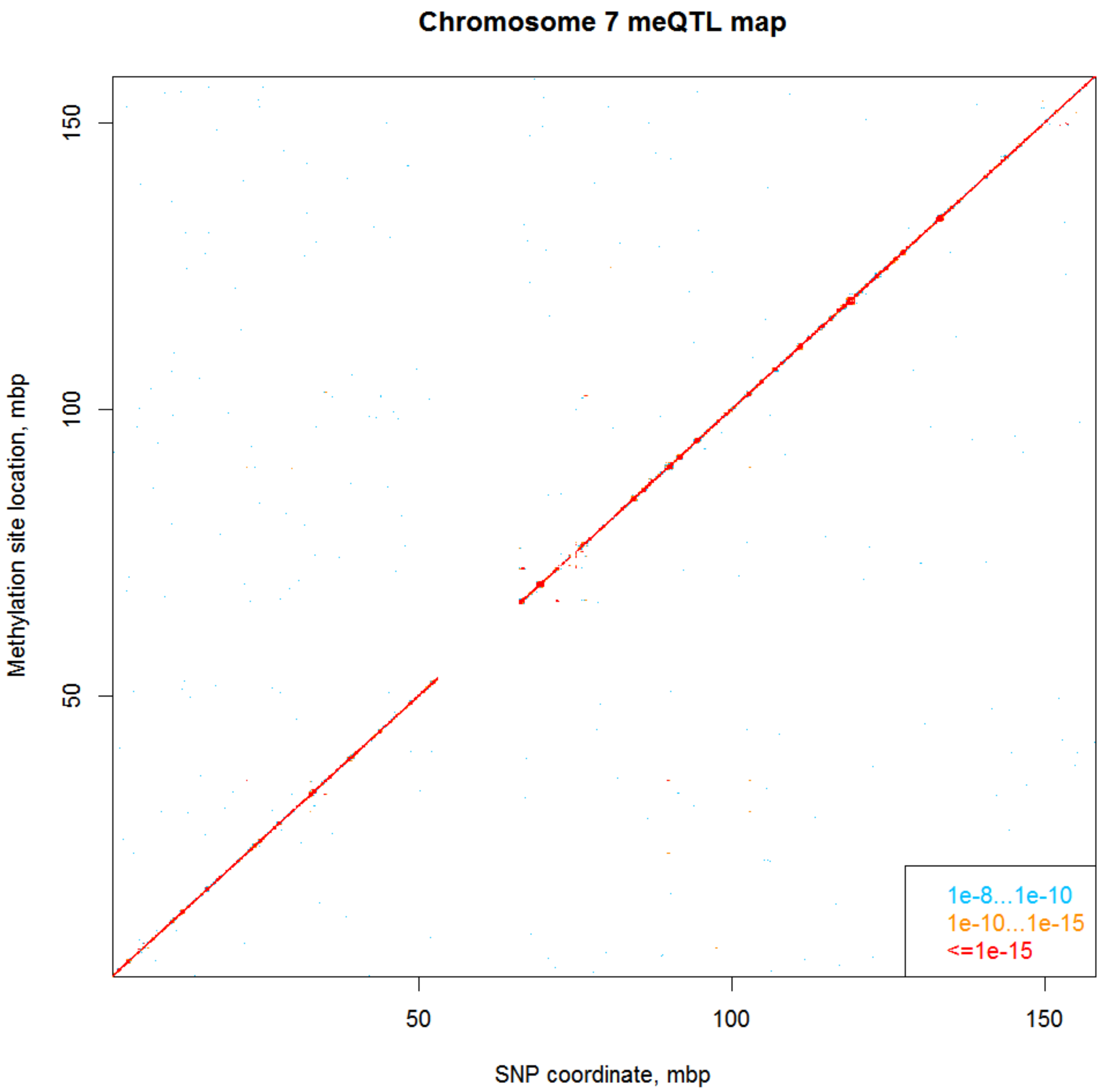
Chromosome 7 meQTL map
Methylation site location, mbp
50
100
150
SNP coordinate, mbp
50
100
150
1e-8...1e-10
1e-10...1e-15
<=1e-15

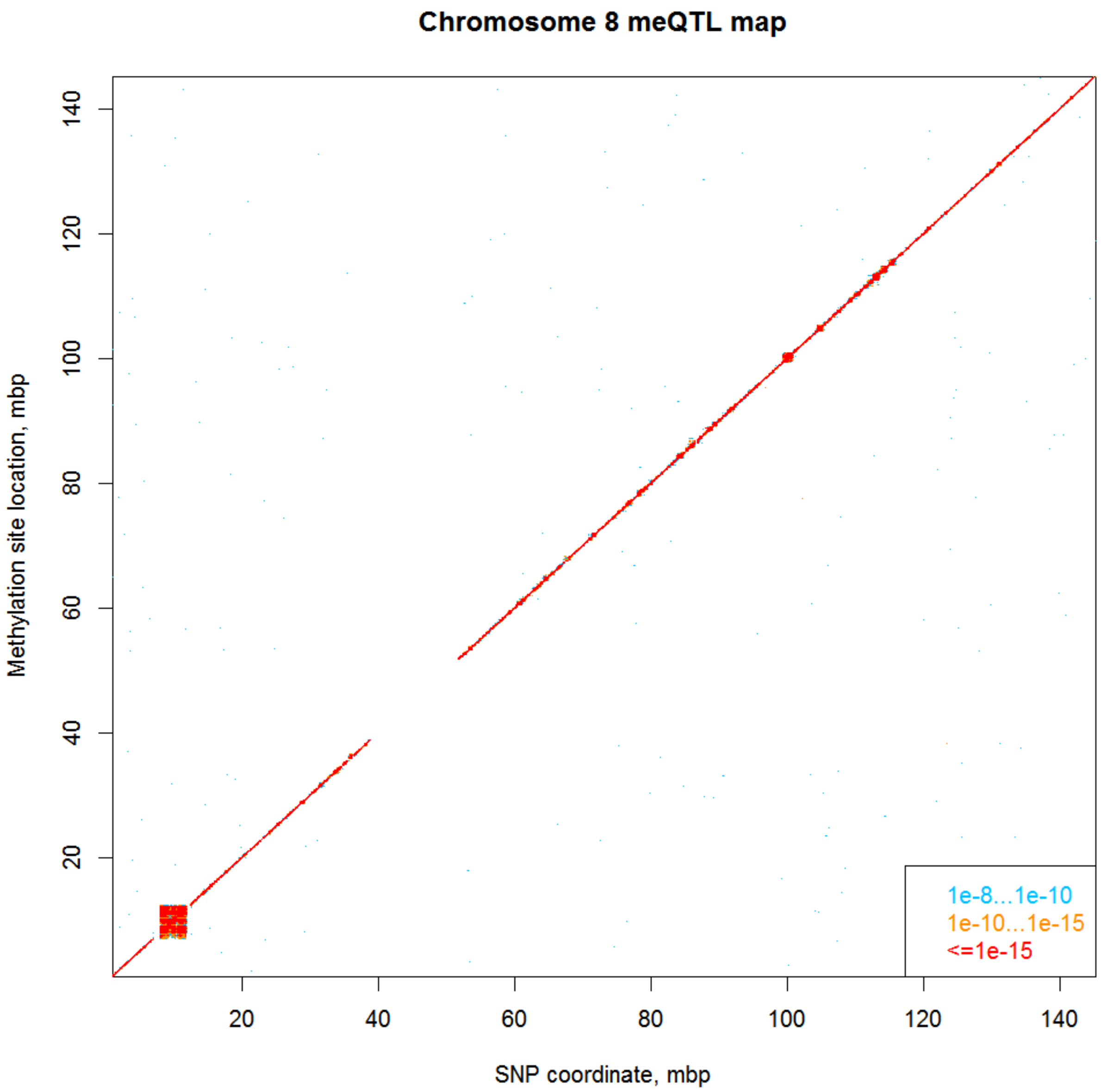

Chromosome 8 meQTL map
Methylation site location, mbp
SNP coordinate, mbp
20
40
60
80
100
120
140
1e-8...1e-10
1e-10...1e-15
<=1e-15

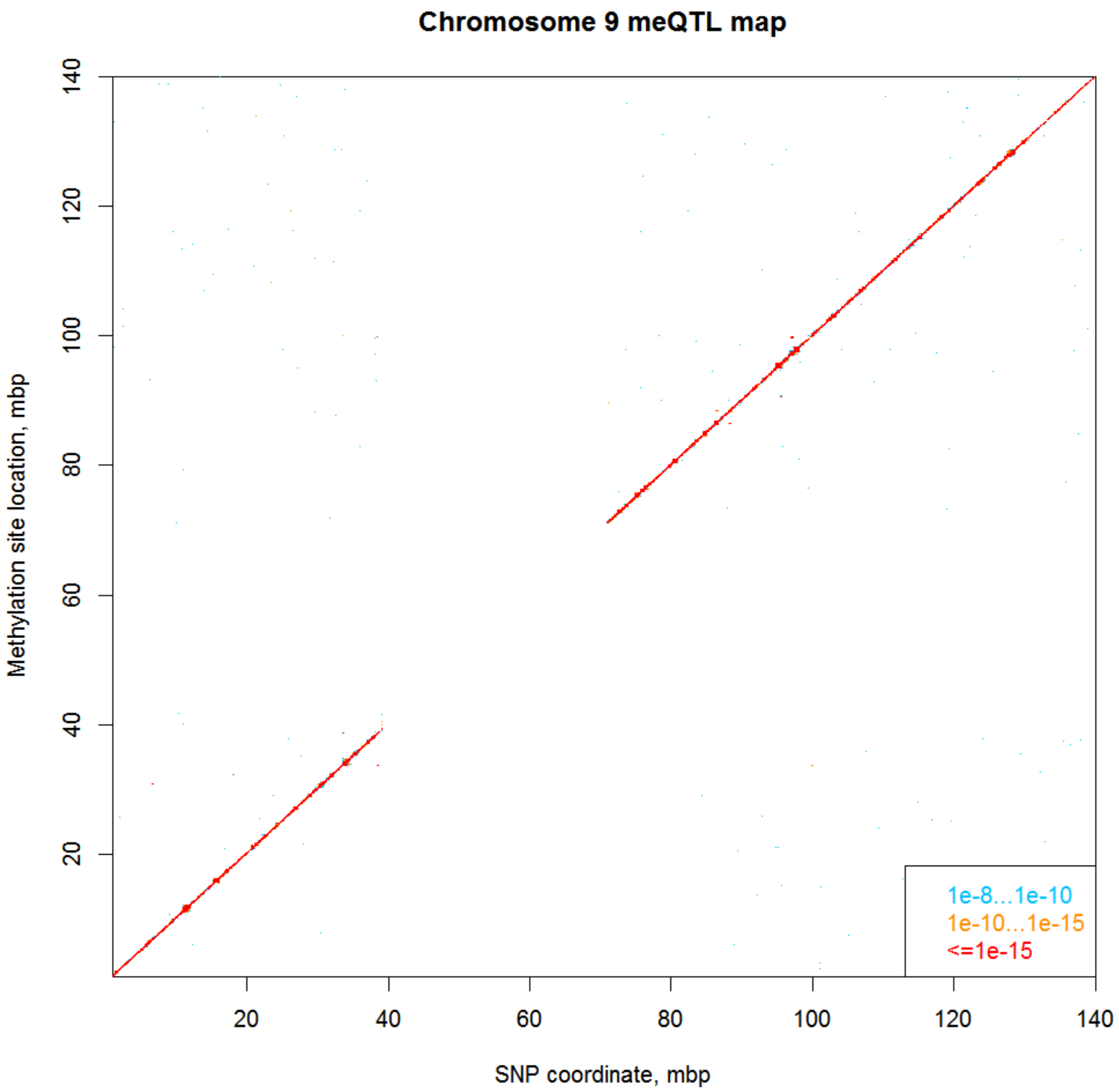
Chromosome 9 meQTL map
Methylation site location, mbp
SNP coordinate, mbp
20
40
60
80
100
120
140
1e-8...1e-10
1e-10...1e-15
<=1e-15

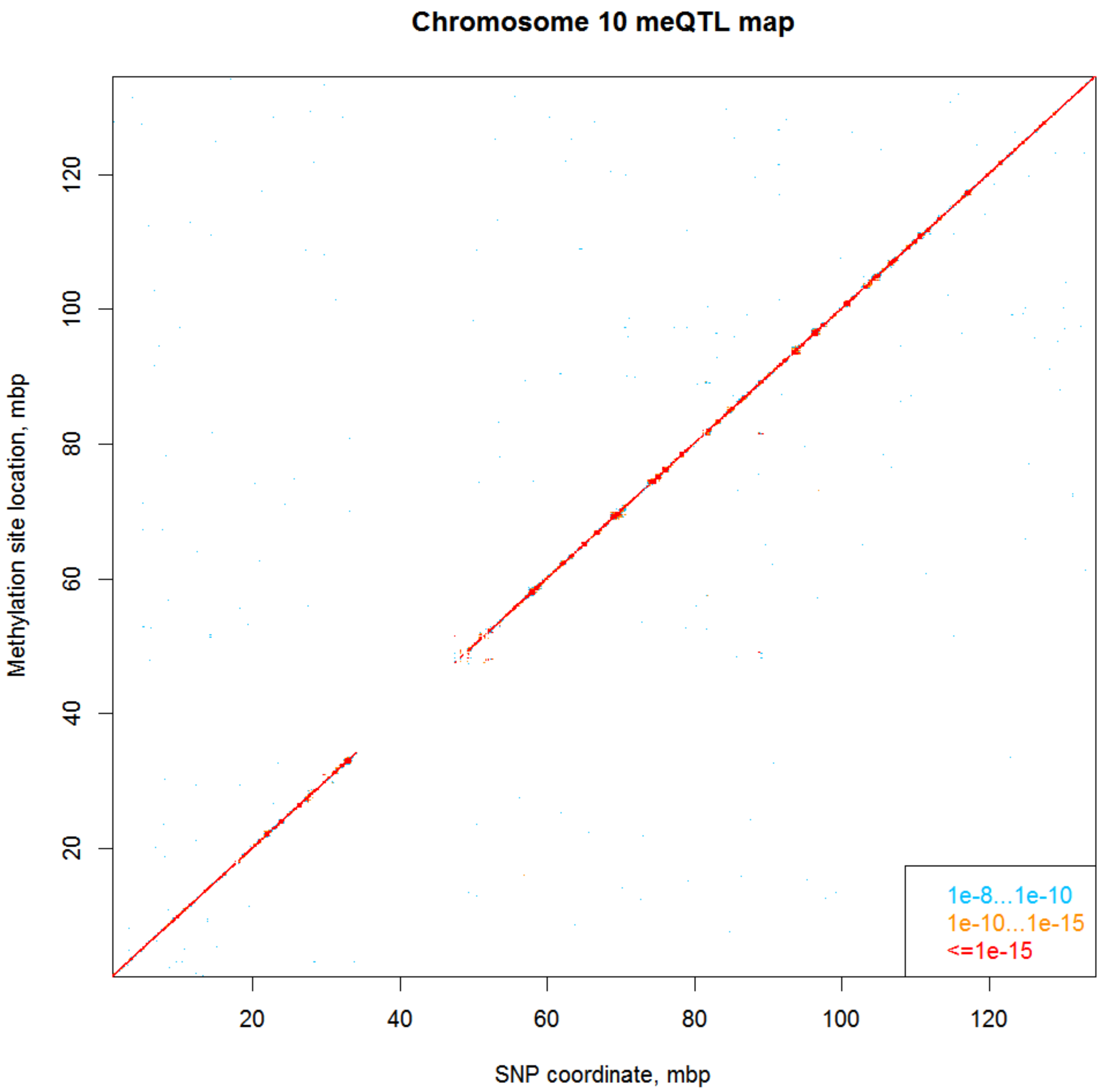
Chromosome 10 meQTL map
Methylation site location, mbp
SNP coordinate, mbp
20
40
60
80
100
120
1e-8...1e-10
1e-10...1e-15
<=1e-15

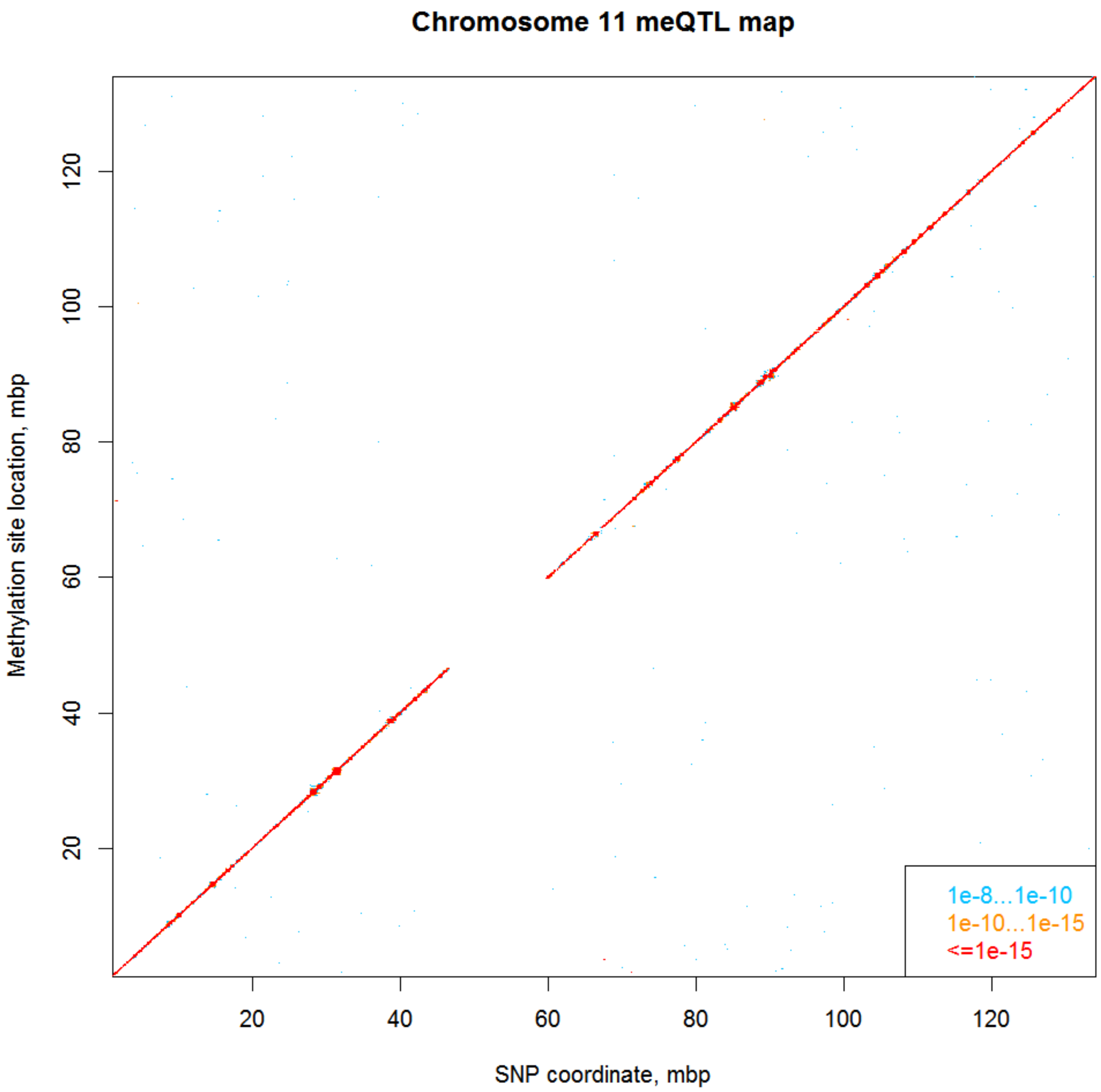
Chromosome 11 meQTL map
Methylation site location, mbp
SNP coordinate, mbp
20
40
60
80
100
120
1e-8...1e-10
1e-10...1e-15
<=1e-15

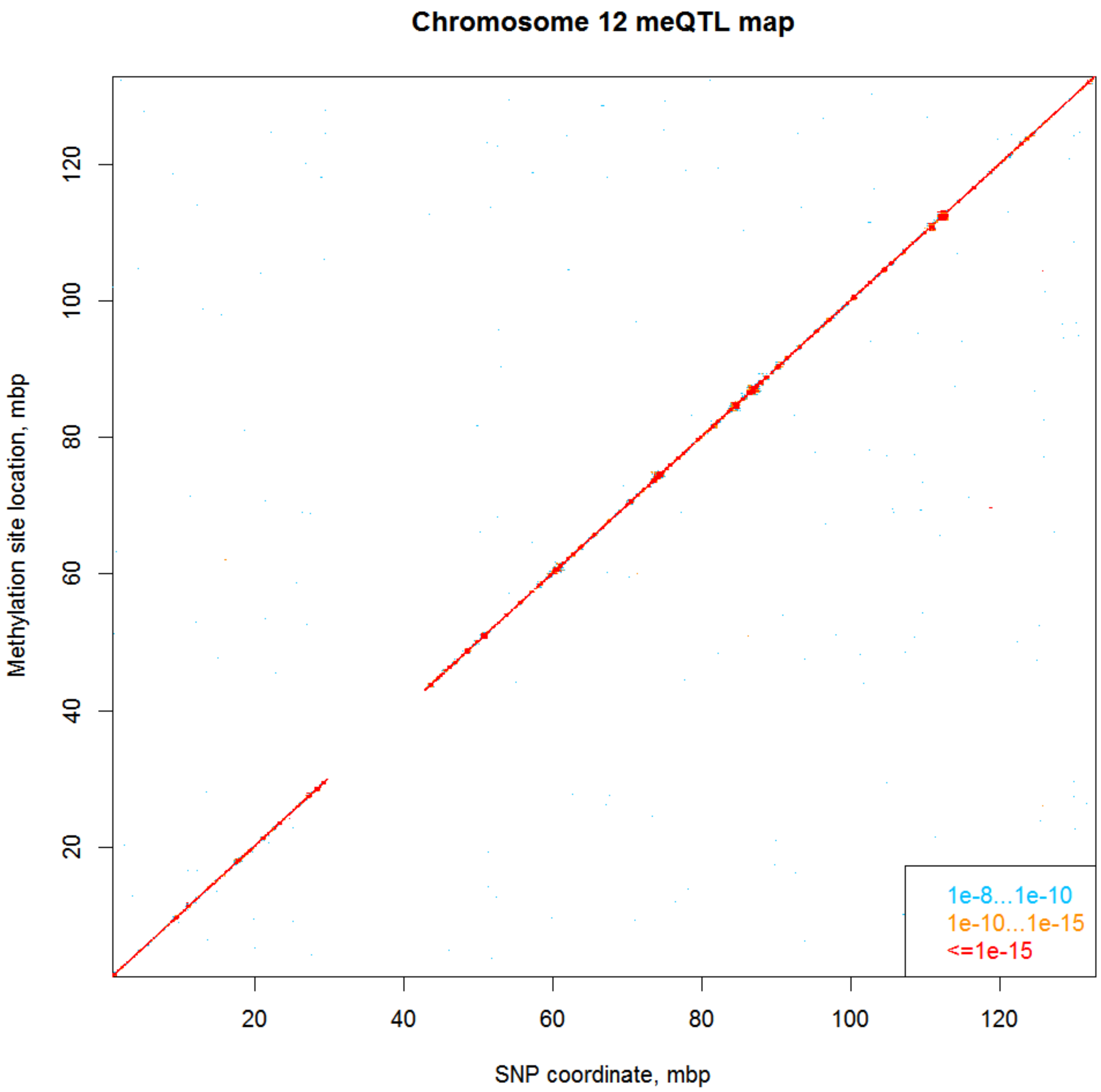
Chromosome 12 meQTL map
Methylation site location, mbp
SNP coordinate, mbp
20
40
60
80
100
120
1e-8...1e-10
1e-10...1e-15
<=1e-15

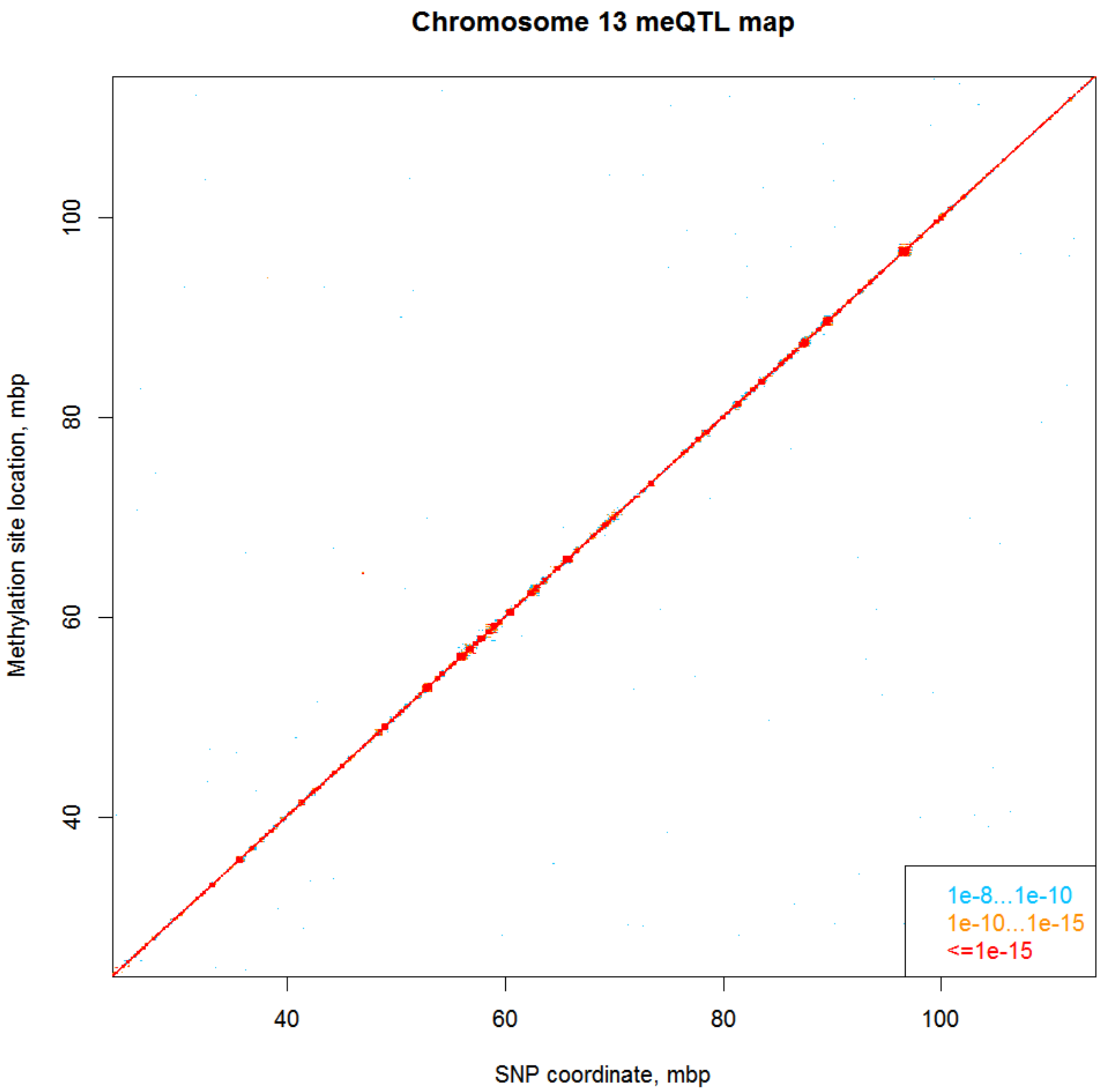
Chromosome 13 meQTL map
Methylation site location, mbp
40
60
80
100
SNP coordinate, mbp
40
60
80
100
1e-8...1e-10
1e-10...1e-15
<=1e-15

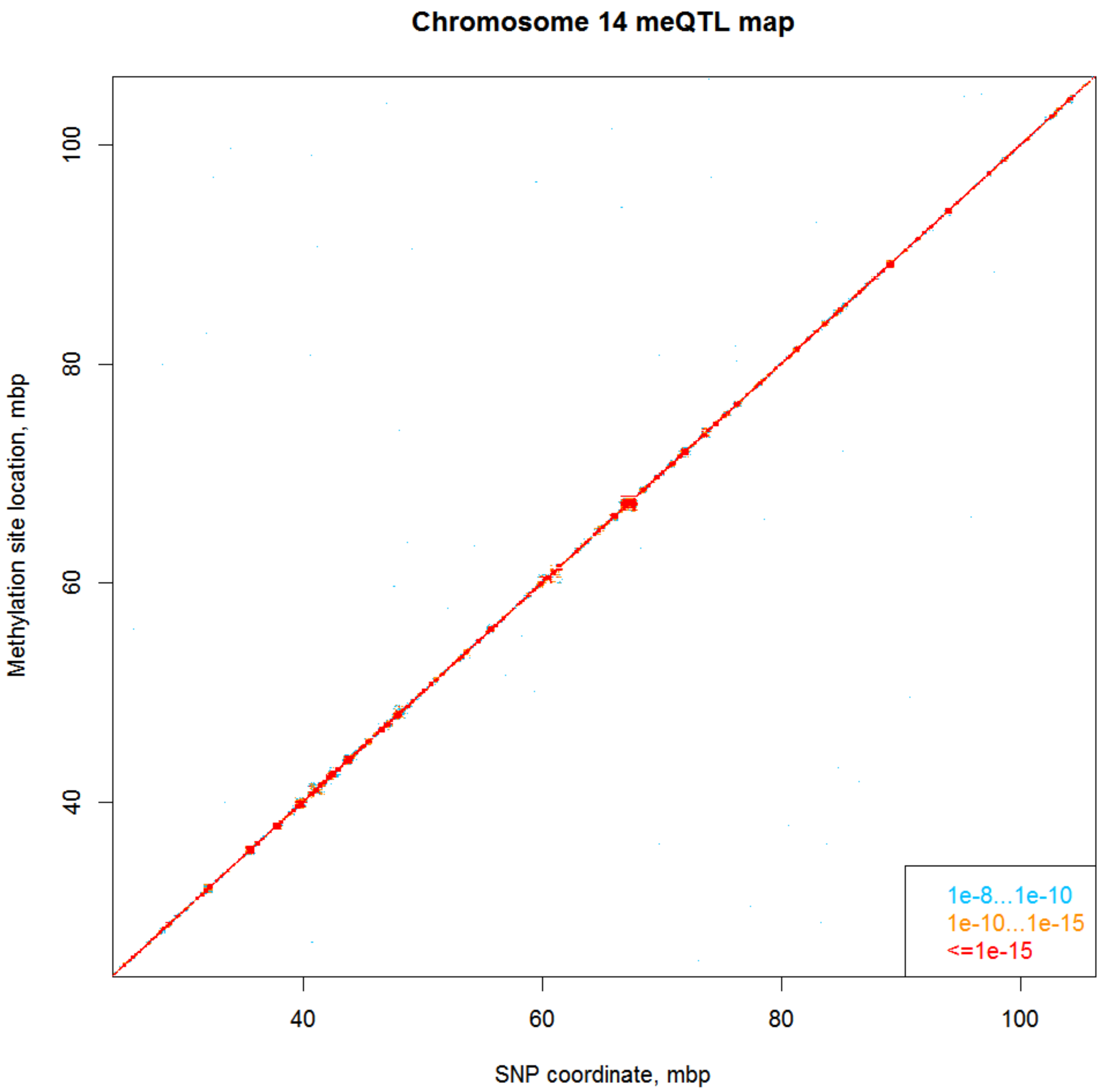
Chromosome 14 meQTL map
Methylation site location, mbp
SNP coordinate, mbp
40
60
80
100
40
60
80
100
1e-8...1e-10
1e-10...1e-15
<=1e-15

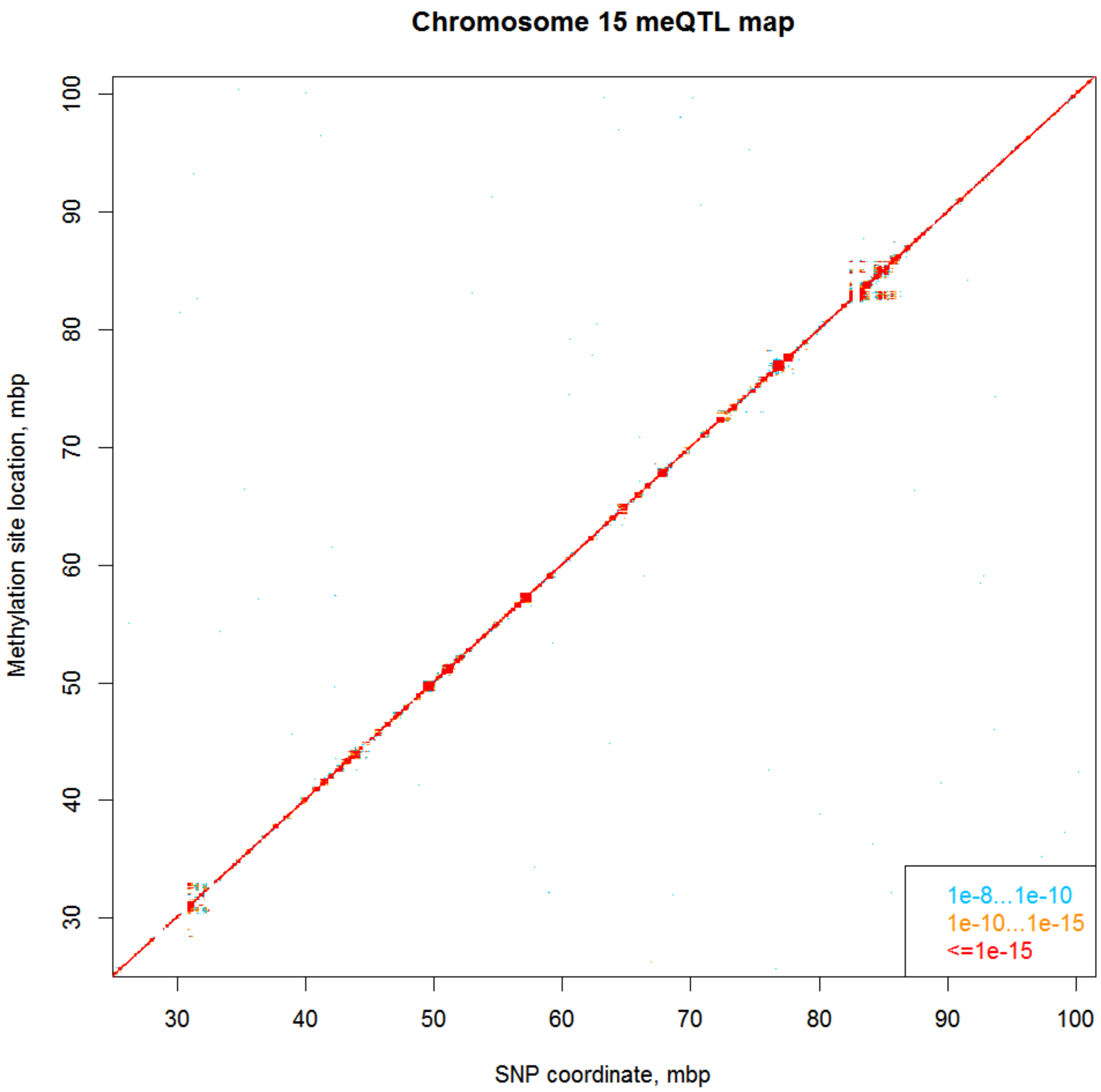
Chromosome 15 meQTL map
Methylation site location, mbp
SNP coordinate, mbp
30
40
50
60
70
80
90
100
1e-8...1e-10
1e-10...1e-15
<=1e-15

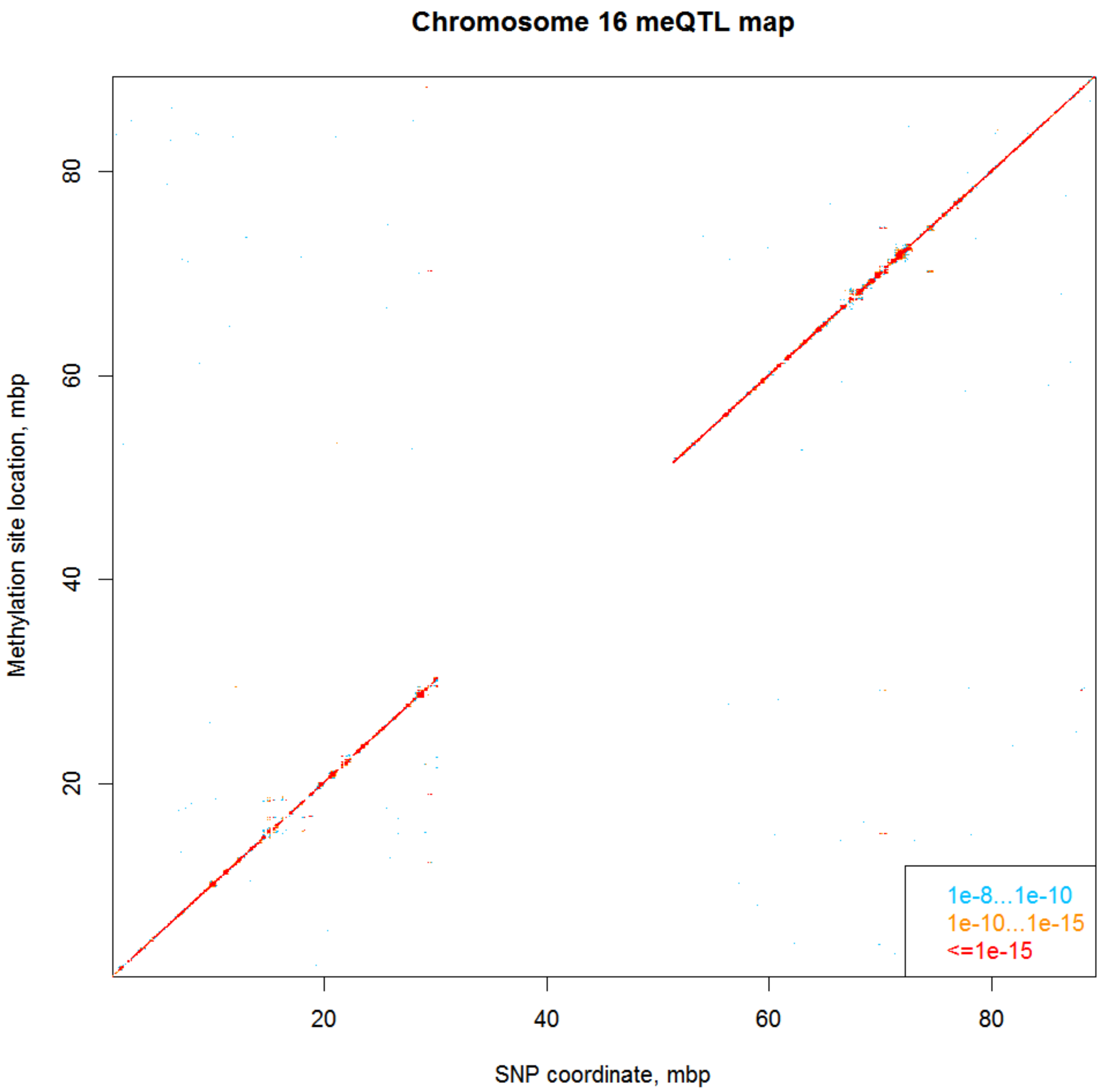
Chromosome 16 meQTL map
Methylation site location, mbp
SNP coordinate, mbp
20
40
60
80
20
40
60
80
1e-8...1e-10
1e-10...1e-15
<=1e-15

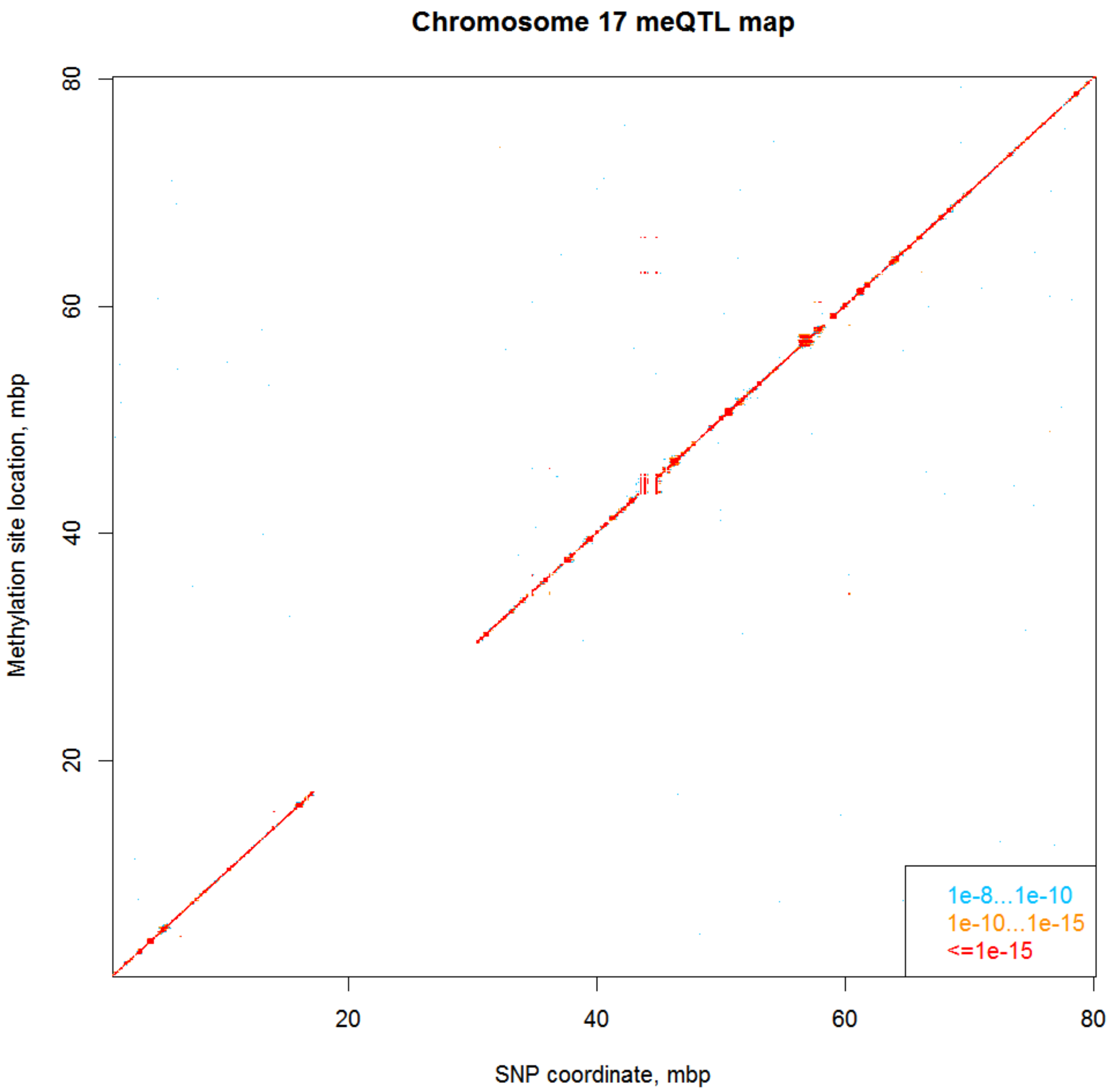
Chromosome 17 meQTL map
Methylation site location, mbp
SNP coordinate, mbp
20
40
60
80
20
40
60
80
1e-8...1e-10
1e-10...1e-15
<=1e-15

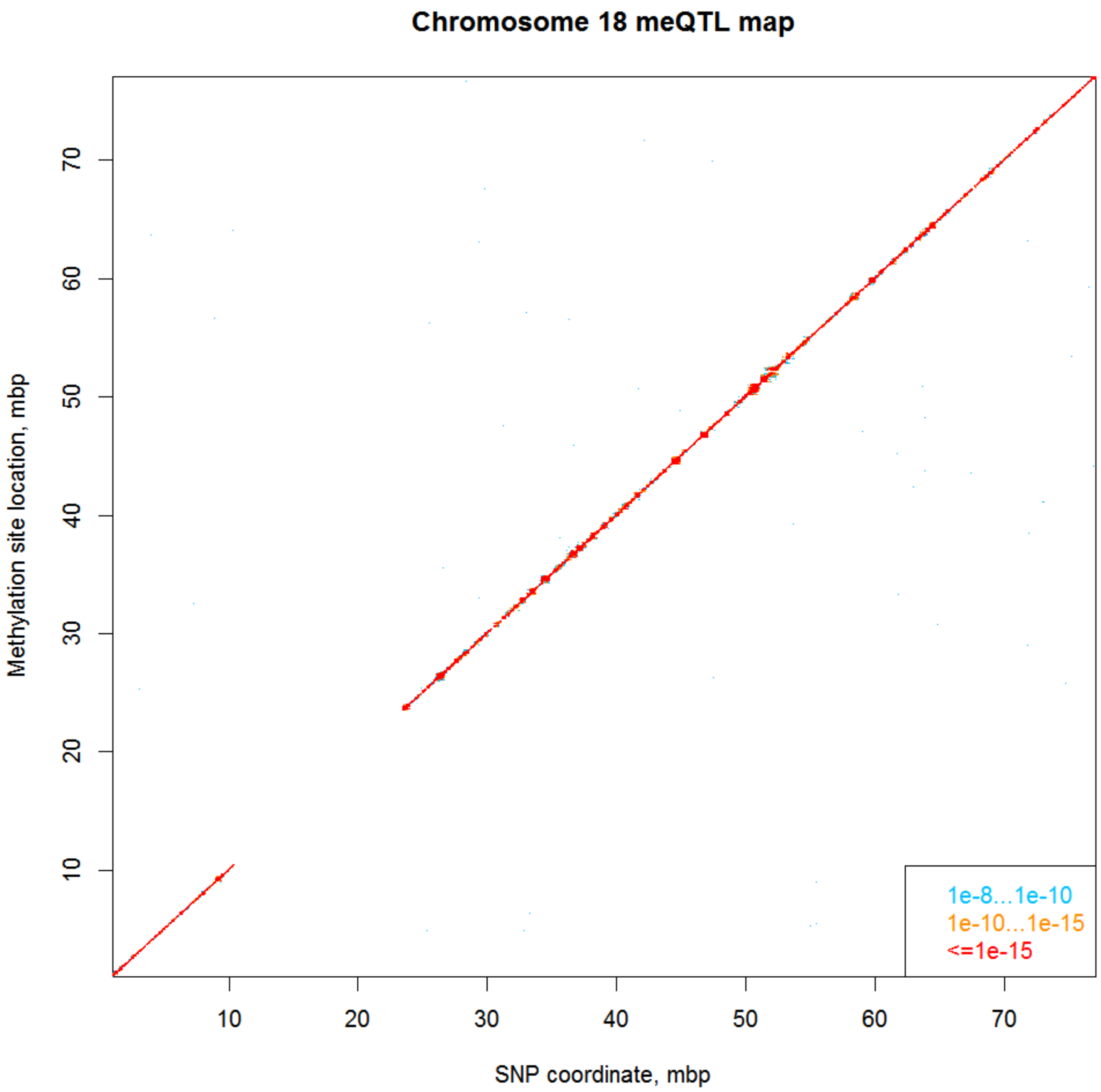
Chromosome 18 meQTL map
Methylation site location, mbp
SNP coordinate, mbp
10
20
30
40
50
60
70
1e-8...1e-10
1e-10...1e-15
<=1e-15

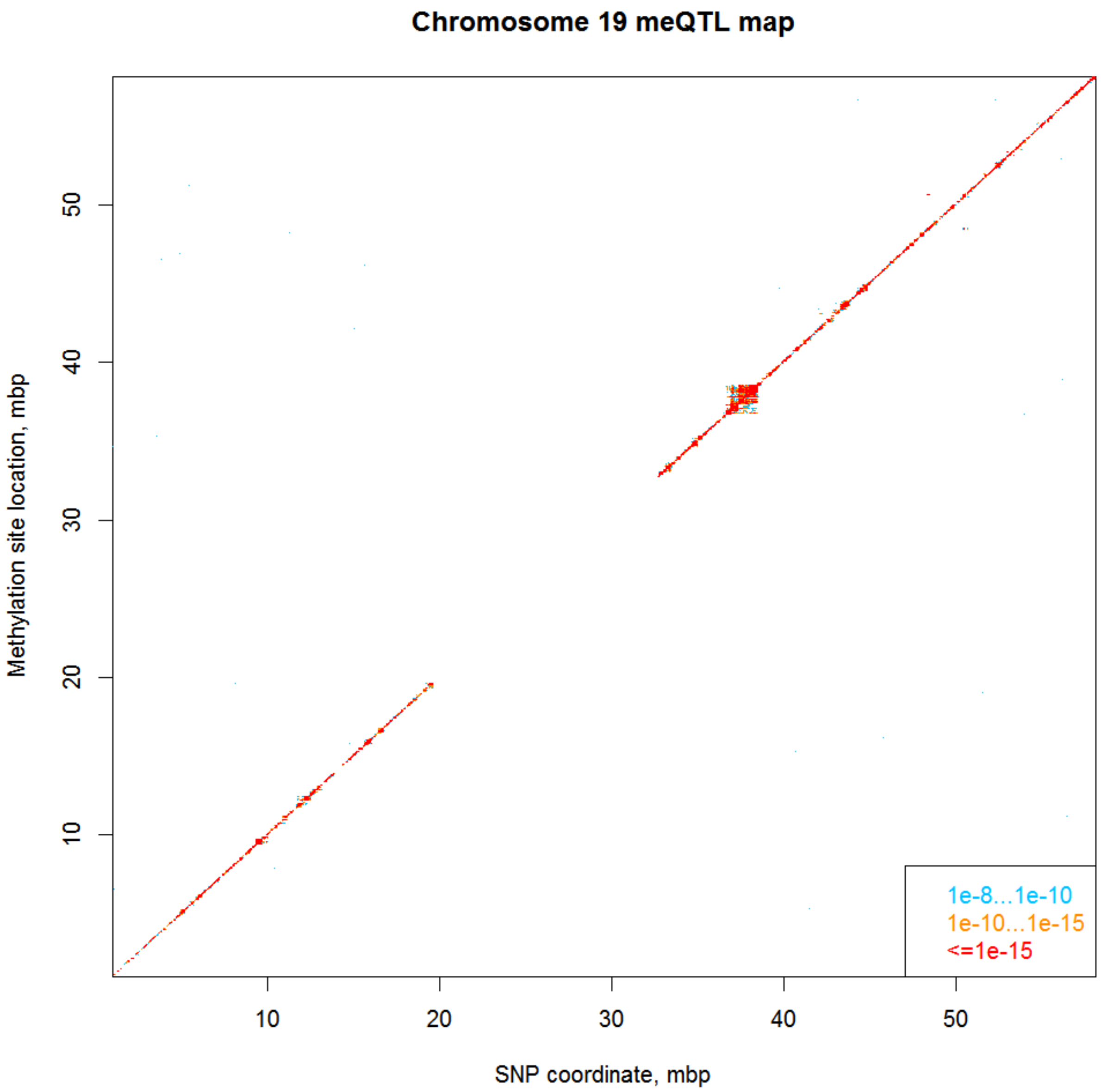
Chromosome 19 meQTL map
Methylation site location, mbp
10
20
30
40
50
SNP coordinate, mbp
10
20
30
40
50
1e-8...1e-10
1e-10...1e-15
<=1e-15

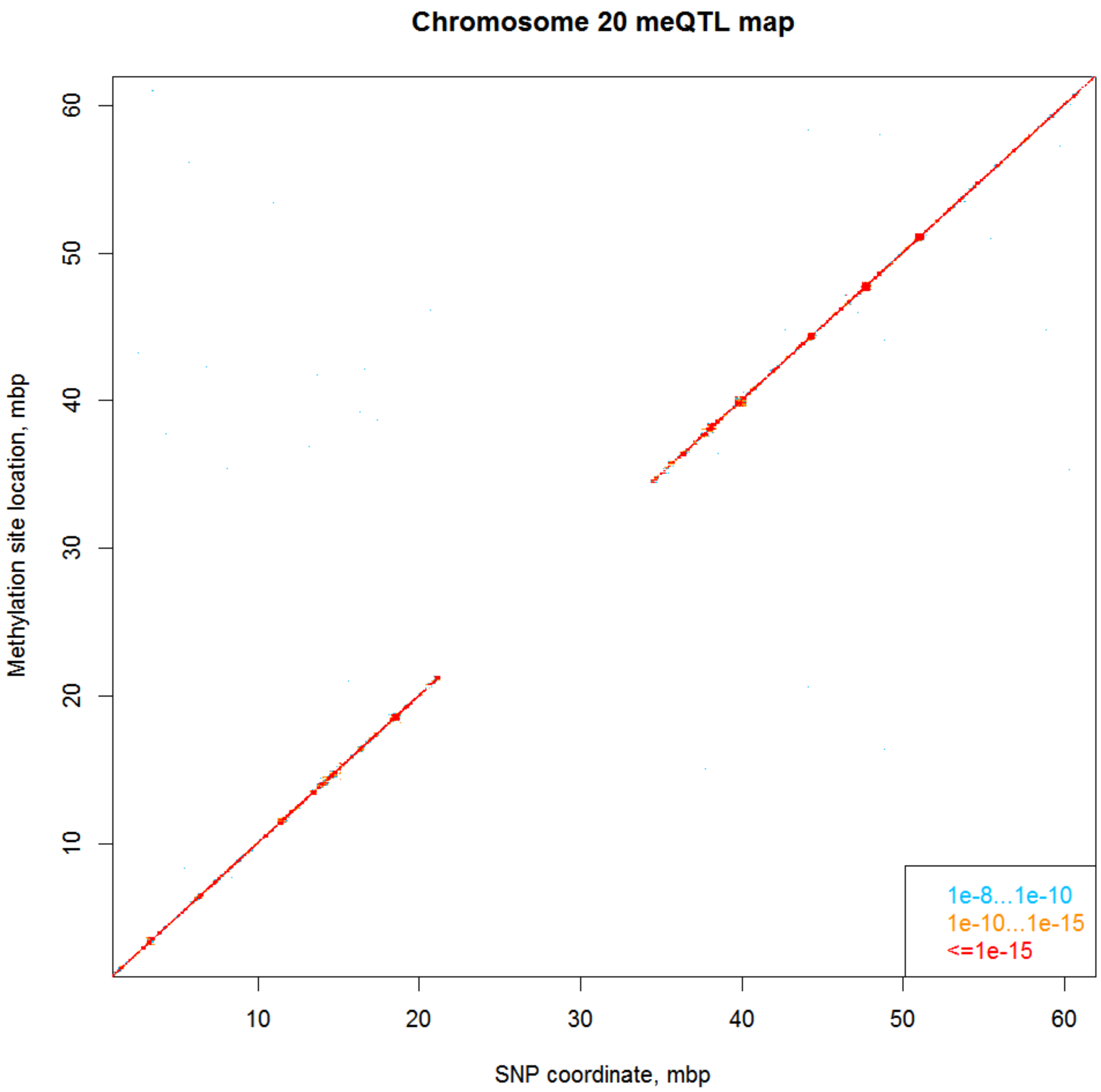
Chromosome 20 meQTL map
Methylation site location, mbp
60
50
40
30
20
10
10
20
30
40
50
60
SNP coordinate, mbp
1e-8...1e-10
1e-10...1e-15
<=1e-15

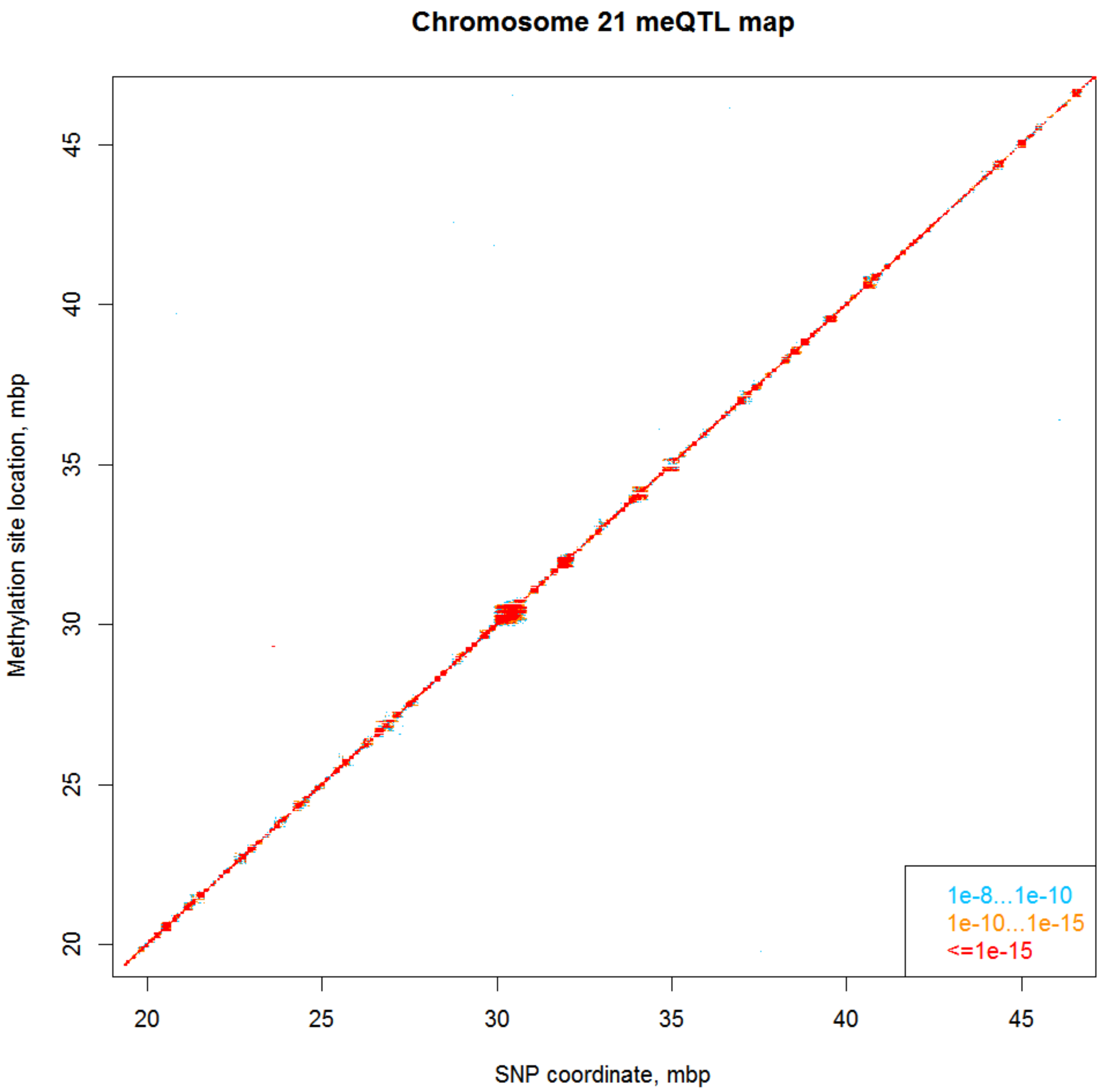
Chromosome 21 meQTL map
Methylation site location, mbp
SNP coordinate, mbp
20
25
30
35
40
45
1e-8...1e-10
1e-10...1e-15
<=1e-15

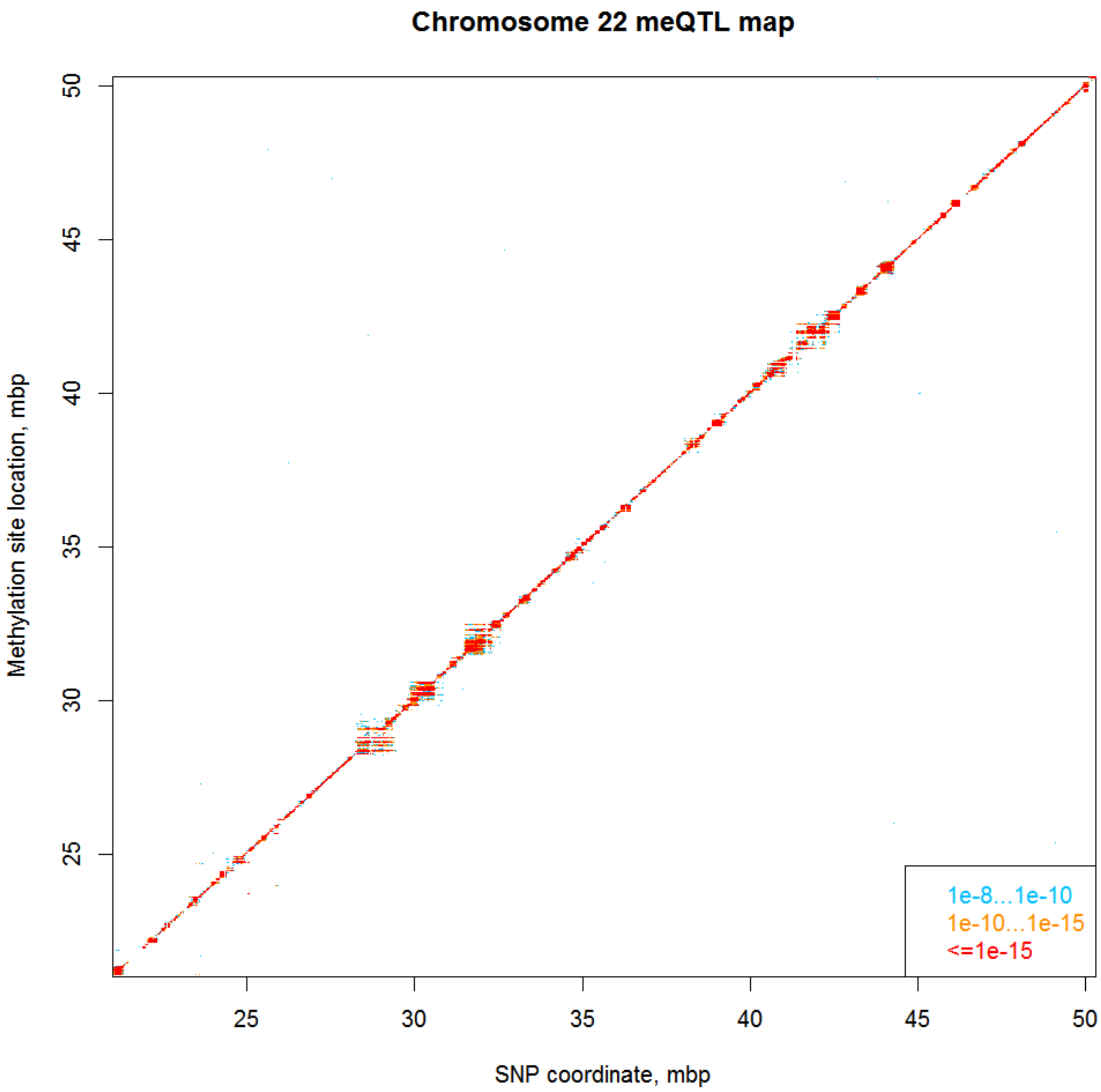
Chromosome 22 meQTL map
Methylation site location, mbp
50
45
40
35
30
25
25
30
35
40
45
50
SNP coordinate, mbp
1e-8...1e-10
1e-10...1e-15
<=1e-15

References

1. Aberg, K.A. et al. Methylome-Wide Association Study of Schizophrenia: Identifying Blood Biomarker Signatures of Environmental Insults. *JAMA Psychiatry* (2014).
2. Bergen, S.E. et al. Genome-wide association study in a Swedish population yields support for greater CNV and MHC involvement in schizophrenia compared with bipolar disorder. *Mol Psychiatry* **17**, 880-886 (2012).
3. Ripke, S. et al. Genome-wide association analysis identifies 13 new risk loci for schizophrenia. *Nat Genet* **45**, 1150-1159 (2013).
4. Alter, O., Brown, P.O. & Botstein, D. Singular value decomposition for genome-wide expression data processing and modeling. *Proc Natl Acad Sci U S A* **97**, 10101-10106 (2000).
5. Price, A.L. et al. Principal components analysis corrects for stratification in genome-wide association studies. *Nat Genet* **38**, 904-909 (2006).
6. Aberg, K.A. et al. MBD-seq as a cost-effective approach for methylome-wide association studies: demonstration in 1500 case--control samples. *Epigenomics* **4**, 605-621 (2012).
7. Bell, J.T. et al. DNA methylation patterns associate with genetic and gene expression variation in HapMap cell lines. *Genome Biol* **12**, R10 (2011).
8. Chen, W. et al. MethylPCA: a toolkit to control for confounders in methylome-wide association studies. *BMC Bioinformatics* **14**, 74 (2013).
9. McClay, J.L. et al. A methylome-wide study of aging using massively parallel sequencing of the methyl-CpG-enriched genomic fraction from blood in over 700 subjects. *Hum Mol Genet* **23**, 1175-1185 (2014).
10. Sun, Y.V. et al. Comparison of the DNA methylation profiles of human peripheral blood cells and transformed B-lymphocytes. *Hum Genet* **127**, 651-658 (2010).
